# Evolution of l‐DOPA 4,5‐dioxygenase activity allows for recurrent specialisation to betalain pigmentation in Caryophyllales

**DOI:** 10.1111/nph.16089

**Published:** 2019-09-29

**Authors:** Hester Sheehan, Tao Feng, Nathanael Walker‐Hale, Samuel Lopez‐Nieves, Boas Pucker, Rui Guo, Won C. Yim, Roshani Badgami, Alfonso Timoneda, Lijun Zhao, Helene Tiley, Dario Copetti, Michael J. Sanderson, John C. Cushman, Michael J. Moore, Stephen A. Smith, Samuel F. Brockington

**Affiliations:** ^1^ Department of Plant Sciences University of Cambridge Tennis Court Road Cambridge CB2 3EA UK; ^2^ CAS Key Laboratory of Plant Germplasm Enhancement and Specialty Agriculture Wuhan Botanical Garden Chinese Academy of Sciences Wuhan 430074 China; ^3^ CeBiTec & Faculty of Biology Bielefeld University Universitaetsstrasse Bielefeld 33615 Germany; ^4^ College of Life Sciences University of Chinese Academy of Sciences Beijing 100049 China; ^5^ Department of Biochemistry and Molecular Biology University of Nevada Reno NV 89577 USA; ^6^ Department of Ecology and Evolutionary Biology University of Michigan Ann Arbor MI 48109 USA; ^7^ Department of Biology Oberlin College Science Center K111 Oberlin OH 44074 USA; ^8^ Arizona Genomics Institute, School of Plant Sciences, University of Arizona Tucson AZ 85721 USA; ^9^ Molecular Plant Breeding Institute of Agricultural Sciences ETH Zurich, Universitaetstrasse 2 8092 Zurich Switzerland; ^10^ Department of Evolutionary Biology and Environmental Studies University of Zurich Winterthurerstrasse 190 8057 Zurich Switzerland; ^11^ Department of Ecology and Evolutionary Biology University of Arizona 1041 E. Lowell St. Tucson AZ 85721 USA

**Keywords:** anthocyanins, betalains, Caryophyllales, convergent evolution, gene duplication, l‐DOPA 4, 5‐dioxygenase (DODA), metabolic operon, plant pigments, specialised metabolism

## Abstract

- The evolution of l‐DOPA 4,5‐dioxygenase activity, encoded by the gene *DODA*, was a key step in the origin of betalain biosynthesis in Caryophyllales. We previously proposed that l‐DOPA 4,5‐dioxygenase activity evolved via a single Caryophyllales‐specific neofunctionalisation event within the *DODA* gene lineage. However, this neofunctionalisation event has not been confirmed and the *DODA* gene lineage exhibits numerous gene duplication events, whose evolutionary significance is unclear.
- To address this, we functionally characterised 23 distinct DODA proteins for l‐DOPA 4,5‐dioxygenase activity, from four betalain‐pigmented and five anthocyanin‐pigmented species, representing key evolutionary transitions across Caryophyllales. By mapping these functional data to an updated DODA phylogeny, we then explored the evolution of l‐DOPA 4,5‐dioxygenase activity.
- We find that low l‐DOPA 4,5‐dioxygenase activity is distributed across the *DODA* gene lineage. In this context, repeated gene duplication events within the *DODA* gene lineage give rise to polyphyletic occurrences of elevated l‐DOPA 4,5‐dioxygenase activity, accompanied by convergent shifts in key functional residues and distinct genomic patterns of micro‐synteny.
- In the context of an updated organismal phylogeny and newly inferred pigment reconstructions, we argue that repeated convergent acquisition of elevated l‐DOPA 4,5‐dioxygenase activity is consistent with recurrent specialisation to betalain synthesis in Caryophyllales.

## Abstract 2

See also the Commentary on this article by Gandía‐Herrero & García‐Carmona, 227: 664–666.

## Introduction

As sessile organisms, plants have exploited metabolic systems to produce a plethora of diverse specialised metabolites (Weng, 2014). Specialised metabolites are critical for survival in particular ecological niches and are often taxonomically restricted (Weng, 2014). In flowering plants, one remarkable example of a taxonomically restricted specialised metabolite occurs in the angiosperm order Caryophyllales (Brockington *et al*., 2011; Timoneda *et al*., 2019). Here, tyrosine‐derived betalain pigments have evolved to replace anthocyanins, which are otherwise ubiquitous across flowering plants (Bischoff, 1876; Clement & Mabry, 1996). Betalains are also present in the fungal lineage Basidiomycota (Musso, 1979) and the bacterial species *Gluconacetobacter diazotrophicus* (Contreras‐Llano *et al*., 2019).

The phylogenetic distribution of betalain pigmentation within Caryophyllales is complex (Brockington *et al*., 2011). In betalain‐producing families within the core Caryophyllales, anthocyanins have not been found (Bate‐Smith, 1962; Clement & Mabry, 1996), although earlier substrates and associated enzymes in the flavonoid pathway have been detected (Shimada *et al*., 2004, 2005; Polturak *et al*., 2018). However, anthocyanins have been reported in six core Caryophyllales families, namely Caryophyllaceae, Molluginaceae, Kewaceae, Limeaceae, Macarthuriaceae and Simmondsiaceae (Clement & Mabry, 1996; Thulin *et al*., 2016). In these six anthocyanic lineages, betalains have not been detected, indicating that anthocyanins and betalains are mutually exclusive (Stafford, 1994; Clement & Mabry, 1996). The six anthocyanic lineages are intercalated with betalain‐pigmented lineages resulting in a homoplastic distribution of these two pigments (Brockington *et al*., 2011). The distribution of anthocyanin and betalain‐pigmented lineages is consistent with multiple origins of betalain pigmentation, a single origin of betalain pigmentation with multiple reversals to anthocyanin, or a combination of these scenarios (Brockington *et al*., 2011).

**Figure 1:**
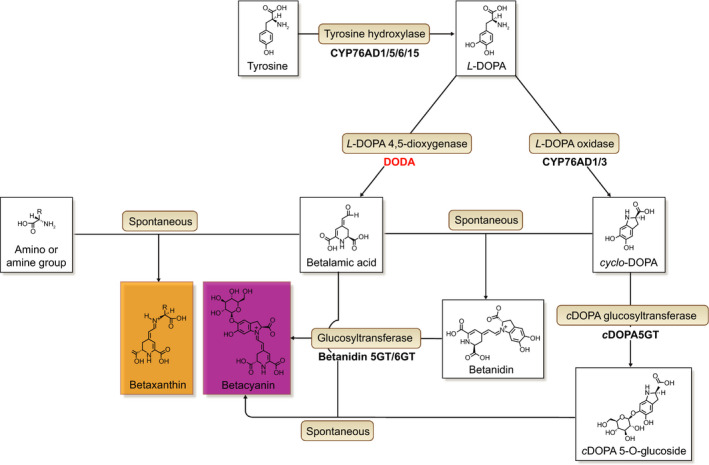
The betalain biosynthetic pathway. A schematic showing the enzymatic and spontaneous reactions that form the specialised metabolites, betalains. The focus of this study is l‐DOPA 4,5‐dioxygenase (DODA; highlighted in red) which catalyses the formation of betalamic acid, the core chromophore of betalain pigments, from l *‐*3,4‐dihydroxyphenylalanine (l‐DOPA). This figure was adapted from Timoneda *et al*. (2019).

In contrast to the anthocyanin pathway, the betalain biosynthetic pathway is relatively simple, involving as few as four enzymatic steps to proceed from tyrosine to stable betalain pigments: yellow betaxanthins and violet betacyanins (Fig. 1). The core genes encoding betalain synthesis enzymes have been elucidated, primarily through heterologous assays in *Saccharomyces cerevisiae* and *Nicotiana benthamiana*, which has emerged as an essential tool for betalain research *in planta* (Polturak *et al*., 2016; Timoneda *et al*., 2018). The key enzymatic step in betalain biosynthesis involves conversion of l‐3,4‐dihydroxyphenylalanine (l‐DOPA) to betalamic acid, the central chromophore of betalain pigments. l‐DOPA 4,5‐dioxygenase is encoded by the gene *DODA*, a member of the *LigB* gene family (Christinet *et al*., 2004). Within Caryophyllales, a gene duplication in the *LigB*/*DODA* gene lineage gave rise to the DODAα and DODAβ clades, with l‐DOPA 4,5‐dioxygenase activity previously inferred to have evolved at the base of the DODAα lineage (Brockington *et al*., 2015). On the basis of this Caryophyllales‐specific DODAα/DODAβ duplication, and subsequent losses of *DODA* *α* loci in anthocyanic lineages, we previously argued for a single origin of betalain pigmentation, with multiple reversals to anthocyanin pigmentation (Brockington *et al*., 2015).

However, evolutionary patterns within the DODAα lineage are complex (Brockington *et al*., 2015). In addition to the DODAα/DODAβ duplication, there have been at least nine duplications in the DODAα lineage resulting in all betalain‐pigmented lineages of Caryophyllales containing at least three *DODA* genes – at least one homologue from the DODAβ lineage and at least two paralogues from the DODAα lineage, with *Beta vulgaris* containing five copies of *DODA* *α*. In *B. vulgaris*, only two DODAα paralogues have been studied (Sasaki *et al*., 2009; Gandía‐Herrero & García‐Carmona, 2012; Hatlestad *et al*., 2012; Chung *et al*., 2015; Bean *et al*., 2018), with one paralogue, BvDODA1 (hereafter termed BvDODAα1), found to exhibit high levels of l‐DOPA 4,5‐dioxygenase activity and the other paralogue, BvDODA2 (hereafter termed BvDODAα2), exhibiting no or only marginal l‐DOPA 4,5‐dioxygenase activity. Bean *et al*. (2018) compared BvDODAα1 and BvDODAα2 and identified seven divergent residues that, when altered in BvDODAα2, were sufficient to allow BvDODAα2 to convert l‐DOPA to betalamic acid in yeast. Like BvDODAα2, two DODAα paralogues from other species (*Parakeelya mirabilis* and a *Ptilotus *hybrid) have also been shown to have limited or no capacity to produce betalamic acid (Chung *et al*., 2015). Extensive gene duplication within the DODAα lineage and the conserved presence of paralogues exhibiting no or only marginal l‐DOPA 4,5‐dioxygenase activity suggests further sub‐ and/or neofunctionalisation events occurring within the DODAα clade, although the evolutionary significance of this is unclear.

In the current study, we explore the evolution of l‐DOPA 4,5‐dioxygenase activity in Caryophyllales, focusing on paralogy within the DODA lineage. In the context of an updated organismal phylogeny and new pigment data, we select species representing key inferred transitions in pigment gain and loss. We then assess their DODA paralogues for levels of l‐DOPA 4,5‐dioxygenase activity using an established heterologous assay in *N. benthamiana*. We use the production of betacyanin in this heterologous assay as a proxy for l‐DOPA 4,5‐dioxygenase activity, with the relative strength of betacyanin production indicating the relative strength of l‐DOPA 4,5‐dioxygenase activity between paralogues. By mapping activity to a comprehensive DODA phylogeny, we reveal that multiple acquisitions of high l‐DOPA 4,5‐dioxygenase activity are linked with repeated gene duplication events within the DODAα lineage. Furthermore, we find that marginal levels of l‐DOPA 4,5‐dioxygenase activity are distributed across the DODAα lineage, implying that marginal levels of activity were present prior to multiple origins of high activity. Recurrent origins of high activity are accompanied by convergent shifts in residues known to be sufficient to confer l‐DOPA 4,5‐dioxygenase activity. We reconcile these data with inferred patterns of pigment evolution and argue that recurrent acquisition of high l‐DOPA 4,5‐dioxygenase activity underlies polyphyletic patterns of betalain pigmentation in Caryophyllales.

## Materials and Methods

### Plant materials and growth conditions

*Beta vulgaris* subsp. *vulgaris* ‘Bolivar’ (referred to as *B. vulgaris*) was obtained from Thompson & Morgan (Ipswich, UK), and *B. vulgaris* subsp. *vulgaris* ‘YTiBv’ was obtained from Syngenta (Basel, Switzerland). Beet plants were grown at the Cambridge University Botanic Garden under natural light and temperature conditions. The seeds of *Limeum aethiopicum* Burm. f., *Kewa bowkeriana* (Sond.) Christenh., *Macarthuria australis* Hügel ex Endl., *Spergularia marina* (L.) Besser, *Cardionema ramosissimum* Weinm. A. Nelson & J.F. Macbr., *Telephium imperati* L., *Pollichia campestris* Aiton, *Corrigiola litoralis* L., *Spergula arvensis* L. and *Simmondsia chinensis* Link C.K. were grown at the Cambridge University Botanic Garden under natural conditions. Fresh tissue of *Polycarpon tetraphyllum* (L.) L. was collected in Florida (Lake Wauburg Recreation Area, Alachua County, FL, USA). *N. benthamiana* is a standard laboratory line that is maintained by selfing and plants were grown in controlled growth rooms with the following conditions: long‐day (16 h : 8 h, light : dark), 20 °C and 60% humidity.

### DNA/RNA extraction and cDNA synthesis

Tissue sampled for extraction was snap frozen and ground frozen using a Tissue Lyser II homogeniser (Qiagen). DNA was extracted using the Qiagen DNeasy Plant Mini Kit and RNA was removed by the Qiagen DNase‐Free RNase Set. RNA extraction was carried out using PureLink Plant RNA Reagent (Invitrogen) and a TURBO DNA‐free kit (Ambion). Both DNA and RNA quantity and quality were assessed by NanoDrop (Thermo Fisher Scientific, Waltham, MA, USA) and agarose gel electrophoresis. An Agilent Technologies Bioanalyzer (Santa Clara, CA, USA) was used to assess the quantity and quality of RNA for transcriptome sequencing. First‐strand cDNA synthesis was performed using BioScript Reverse Transcriptase (Bioline Reagents, London, UK) and an oligo dT primer. All protocols were carried out according to the manufacturers’ specifications unless otherwise specified.

### Transcriptome and genome sequencing and assembly

Transcriptomes of fresh young leaves of *S. chinensis* and fresh young leaves and flowers of *K. bowkeriana* were sequenced at BGI using BGISEQ (Hong Kong, China). Downstream processing and assembly optimisation were performed following Haak *et al*. (2018). Genome sequencing of *C. litoralis*,* L. aethiopicum*,* S. chinensis* and *S. arvensis* was performed on a HiSeq X‐Ten with one sample per lane and assembled following Pucker *et al*. (2019). Full details can be found in Supporting Information Methods S1.

### Isolating DODA genes for functional analysis

For species with sequenced genomes (*B. vulgaris*,* Mesembryanthemum crystallinum*,* Carnegiea gigantea*,* Kewa caespitosa*), *DODA* sequences were identified by blast searches of genomes and annotated gene files. Annotated gene sequences were checked manually and adjusted if necessary (see Methods S2). For species for which no published genome was available, diverse strategies were used to obtain the full‐length coding sequences. For *Stegnosperma halimifolium*,* M. australis*,* P. tetraphyllum* and *L. aethiopicum*, full‐length coding sequences were recovered from transcriptomes. For *C. ramosissimum*, a partial coding sequence was recovered from RNAseq, and then RACE PCR was performed using the RACE System for Rapid Amplification of cDNA Ends Kit (Thermo Fisher Scientific) according to the manufacturer's instructions in order to amplify the 3′ end of *CrDODAa*. For *P. campestris*,* S. marina* and *T. imperati*, degenerate PCR primers were designed based on known *DODA* *α* sequences in order to amplify a partial sequence, then inverse PCR was used to obtain the full‐length coding sequences, following the protocol described by Ren *et al*. (2005). The full‐length coding sequences for *DODA* genes were isolated from cDNA or gDNA by PCR using Phusion High‐Fidelity DNA polymerase (Thermo Fisher Scientific), and then cloned into pBlueScript SK (New England Biolabs, Hitchin, UK) and verified by Sanger sequencing (Source BioScience, Nottingham, UK); for *M. crystallinum*,* C. gigantea* and *S. halimifolium*,* DODA* genes were synthesised by BioMatik (Cambridge, Canada), Twist Bioscience (San Francisco, CA, USA) and Integrated DNA Technologies (Iowa, IA, USA), respectively. Oligonucleotides are listed in Table S1 and the sequences have been deposited in GenBank (Table S2).

### Species phylogeny and pigment reconstruction

To enable trait reconstruction across the order Caryophyllales, we generated a comprehensive genus‐level species tree using publicly available sequence data compiled by pyphlawd (Smith & Walker, 2019), with constraints of the backbone topology from Walker *et al*. (2018) and Thulin *et al*. (2016, 2018). We calibrated branch lengths to time using treepl (Smith & O'Meara, 2012), which implements the penalised likelihood approach of Sanderson (2002). Full details can be found in Methods S3. Pigment data at genus resolution were used to reconstruct the evolution of betalain pigmentation on the time‐calibrated genus‐level phylogeny of Caryophyllales. We surveyed the literature for pigment data and determined the pigmentation status of 174 genera, classifying them as anthocyanin‐pigmented, betalain‐pigmented or unknown (Table S3). We reconstructed ancestral states using maximum likelihood (Pagel, 1994, 1999) and Bayesian inference via stochastic mapping (Huelsenbeck *et al*., 2003; Bollback, 2006), using the R packages ape v.5.0 and phytools v.0.6‐70, respectively in r v.3.6.0 (Revell, 2012; Paradis & Schliep, 2019; R Core Team, 2019) under an equal rate and an asymmetric rate model. For stochastic mapping, we enforced a prior that the root of Caryophyllales was anthocyanin‐pigmented. Full details can be found in Methods S4.

### DODA gene phylogeny and ancestral sequence reconstruction

We compiled a dataset of publicly available and early release genome and transcriptome assemblies (Table S4) and used a baited search approach with iterative refinement (Lopez‐Nieves *et al*., 2018) to infer a gene tree of *DODA* sequences in Caryophyllales. Full details can be found in Methods S5. To create a sequence dataset computationally and numerically tractable for ancestral sequence reconstruction, we used a custom python script to subsample the *DODA* *α* gene tree (Fig. S1), using a strategy designed to maintain within‐paralogue diversity. We created a final dataset of 198 sequences (indicated in Table S5), ensuring that a representative of all functionally characterised *DODA* *α* loci was included. Ancestral sequence reconstructions were conducted for codons and amino acids in iq‐tree v.1.6.10 (Nguyen *et al*., 2015; Kalyaanamoorthy *et al*., 2017). All scripts, alignments and trees are available on GitHub (https://github.com/NatJWalker-Hale/DODA). Full details can be found in Methods S6.

### Vector generation and transient expression assay

Construction of the multigene vectors containing the genes of the betalain biosynthetic pathway (*DODA*,* BvCYP76AD1*,* MjcDOPA‐5GT*) was carried out using MoClo GoldenGate cloning following the protocol described (Engler *et al*., 2014; Timoneda *et al*., 2018) in order to produce level 2 binary vectors (Fig. S2). *DODA* genes were cloned into level 0 vectors using their coding sequence, except *PtDODAa* for which the gDNA sequence was used. Level 1 vectors were verified by sequencing and level 2 vectors were verified by restriction digests. Transient expression using agroinfiltration of *N. benthamiana* was performed as described previously (Timoneda *et al*., 2018). The following were used as controls in every experiment: positive, pBC‐BvDODAα1; negative, pLUC (Fig. S2). Upon transient transformation in *N. benthamiana*,* DODA* genes encoding enzymes that carry out the l‐DOPA 4,5‐dioxgenase reaction necessary for betalamic acid production will produce betalains. For instance, the previously characterised *B. vulgaris* DODA, BvDODAα1 (Hatlestad *et al*., 2012), exhibits high levels of l‐DOPA 4,5‐dioxygenase activity as inferred by a high level of betacyanin production under heterologous expression (Polturak *et al*., 2016; Timoneda *et al*., 2018). Accordingly, we use the production of betacyanin in this heterologous assay as a proxy for l‐DOPA 4,5‐dioxygenase activity, with the relative strength of betacyanin production indicating the relative strength of l‐DOPA 4,5‐dioxygenase activity between loci. The assay is designed to give a clear comparative measure of biologically relevant levels of l‐DOPA 4,5‐dioxygenase activity *in planta*. We categorise the levels as high l‐DOPA 4,5‐dioxygenase activity (hereafter also referred to as ‘high activity’), low or marginal l‐DOPA 4,5‐dioxygenase activity (hereafter also referred to simply as ‘marginal activity’), or no l‐DOPA 4,5‐dioxygenase activity (hereafter also referred to as ‘no activity’).

### Betalain quantification using HPLC

A single sample was taken from each infiltration spot 4 d post‐infiltration, snap frozen in liquid nitrogen and stored at −80 °C until needed. Samples were homogenised frozen using a single 5 mm glass bead in a Tissue Lyser II homogeniser (Qiagen). Betalains were extracted overnight at 4°C in 80% aqueous methanol containing 50 mM ascorbic acid with a volume of 1 ml extraction buffer per 50 mg fresh weight of leaf tissue. After extraction, the samples were clarified twice by centrifugation at 21 130 ***g*** for 10 min. HPLC analysis was performed using a Thermo Fisher Scientific Accela HPLC autosampler and pump system incorporating a photodiode array detector. Betalains were separated using a Luna Omega column (100 Å, 5 μm, 4.6 × 150 mm) from Phenomenex (Torrance, CA, USA) under the following conditions: 3 min, 0% B; 3–19 min, 0–75% B; 7 min, 0% B where mobile phase A was 0.1% formic acid in 1% acetonitrile and solvent B was 100% acetonitrile, and at a flow rate of 500 μl min^−1^. We quantified the betacyanin compound, betanin, because it has been shown to be the predominant pigment arising from the transient expression assay (Timoneda *et al*., 2018). Betanin was detected by UV/VIS absorbance at a wavelength of 540 nm. Identification and quantification of betanin was carried out using a commercially available *B. vulgaris* extract (Tokyo Chemical Industry UK Ltd, Oxford, UK) and a pure betanin standard (provided by F. Gandía‐Herrero, Universidad de Murcia, Spain). Negative controls (uninfiltrated tissue or pLUC) were set as background and removed from all other samples.

### Synteny analysis of gene cluster

Synteny from sequenced genomes was evaluated to explore the conservation of clustering of *BvDODA* *α* *1* and *BvCYP76AD1* as observed in *B. vulgaris* (Brockington *et al*., 2015). *BvDODA* *α* *1* (Hatlestad *et al*., 2012) and *BvCYP76AD1* (DeLoache *et al*., 2015; Polturak *et al*., 2016; Sunnadeniya *et al*., 2016) were identified from the *B. vulgaris* genome and the related microsynteny was visualised by mcscanx (Wang *et al*., 2012). Pairs of homologous genes from the genomes of *Amaranthus hypochondriacus*,* Chenopodium quinoa*,* B. vulgaris* and *M. crystallinum* were identified using last with default parameters (Kiełbasa *et al*., 2011). Only restricted syntenic regions containing collinear genes along with their neighbouring genes were evaluated.

## Results

### Ancestral state reconstruction of pigmentation in Caryophyllales suggests at least four origins of betalain pigmentation

**Figure 2:**
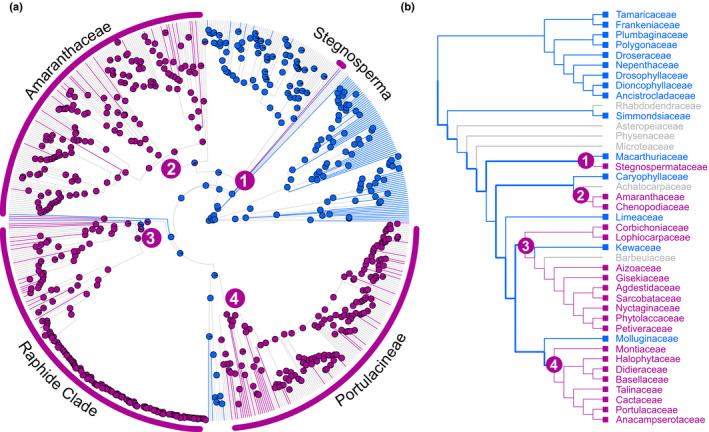
Reconstruction of pigment state across the Caryophyllales supports four origins of betalain pigmentation. (a) Bayesian ancestral state reconstructions of pigmentation on a time‐calibrated, genus‐level maximum likelihood species tree of Caryophyllales, inferred from seven nuclear and plastid markers. Tips are coloured according to pigmentation state: anthocyanin (blue), betalain (pink) and unknown (grey). Pie charts at nodes give posterior probabilities at nodes for anthocyanin (blue) and betalain (pink), inferred from *n *=* *1000 stochastic mapping simulations under the asymmetric (ARD) model of character evolution. (b) The reconstruction shown in (a) simplified to show family‐level relationships. Numbers represent the four inferred origins of betalain pigmentation. Branches in bold indicate relationships that were constrained during tree inference.

We inferred a time‐calibrated, genus‐level maximum likelihood species tree for 640 genera of Caryophyllales, constraining our inference to match the most recent phylogenomic hypotheses (Walker *et al*., 2018). Our inferred topology and divergence times agree well with the current understanding of Caryophyllales (Walker *et al*., 2018), with some minor or unsupported incongruences (Figs 2, S3). Our updated pigmentation dataset contains data for 174 genera or 27% of genera represented in the tree topology. Maximum likelihood reconstruction under a symmetric (ER) and an asymmetric (ARD) model produced the same inferences with four transitions from anthocyanins to betalains predicted – in Stegnospermataceae, Amaranthaceae, the raphide clade (*sensu* Stevens, 2017) and the Portulacineae – and one reversal from betalains to anthocyanins in Kewaceae (Fig. S4). The ER model generated slightly more equivocal reconstructions along the backbone of Caryophyllales than the ARD model but statistical support for each model is nearly equivalent (ΔAIC_ARD‐ER_ = 0.23). Posterior probabilities of node states from Bayesian reconstruction under both models similarly suggested four transitions from anthocyanins to betalains along the backbone of the tree and one reversal (Figs 2, S4).

### Betalain‐pigmented species are inferred to contain a minimum of three *DODA* genes with at least two *DODAα* and one *DODAβ*

We used a baited search approach to populate an expanded *DODA* gene tree containing 318 Caryophyllales species from 34 families (increased from the 95 species and 26 families previously analysed; Brockington *et al*., 2015). The phylogeny includes denser sampling from the anthocyanin‐pigmented lineages Caryophyllaceae and Molluginaceae, and additionally samples the anthocyanin‐pigmented lineages Macarthuriaceae, Kewaceae and Limeaceae, and the betalain‐pigmented lineage Stegnospermataceae. The phylogeny is mostly congruent with earlier analyses, revealing a gene duplication after the divergence of Physenaceae, resulting in two well‐supported paralogues corresponding to DODAα and DODAβ clades in Brockington *et al*. (2015) (Figs S5, S6). Further duplications have occurred, particularly in the DODAα lineage, so that betalain‐pigmented species are inferred to contain at least three genes predicted to encode a full‐length DODA protein, with at least two DODAα and one DODAβ (Figs S5, S6).

### DODAα homologues are present in a wide range of anthocyanic lineages across the Caryophyllales

We performed a search for *DODA* loci across all anthocyanic lineages (Macarthuriaceae, Limeaceae, Kewaceae, Caryophyllaceae and Molluginaceae). Here, we recovered single *DODA* *α* genes from seven species representing four early diverging lineages of Caryophyllaceae: *P. tetraphyllum* (Polycarpaeae), *C. ramosissimum* and *P. campestris* (Paronychieae), *T. imperati* and *C. litoralis* (Corrigioleae), and *S. marina* and *S. arvensis* (Sperguleae; Figs S5, S6). The *DODA* *α* genes from two of these species, *C. litoralis* and *P. campestris*, were found to have mutations causing premature stop codons (Fig. S7). We then searched for *DODA* genes in anthocyanic *M. australis* (Macarthuriaceae), *L. aethiopicum* (Limeaceae), *K. caespitosa* (Kewaceae) and *P. exiguum* (Molluginaceae) using trancriptome and genome sequencing. We detected a *DODA*β gene in all four species (Figs S5, S6). Single *DODA* *α* genes were recovered from *M. australis* and *L. aethiopicum*, and two *DODA* *α* homologues were recovered from *K. caespitosa*. A *DODA* *α* gene could not be detected in *P. exiguum* despite *c*. 206× short read coverage based on its estimated genome size (Pucker *et al*., 2019).

### DODAβ homologues exhibit no or negligible amounts of l‐DOPA 4,5‐dioxygenase activity

**Figure 3:**
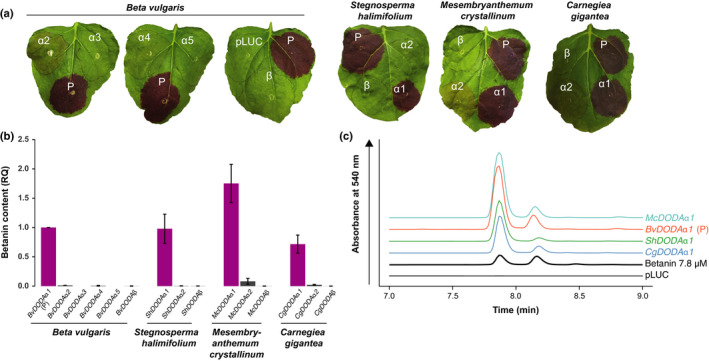
Betalain‐pigmented species have a single DODA that has high activity when heterologously expressed in *Nicotiana benthamiana* and other DODA that have no or marginal activity. (a) A representative leaf is shown from the agroinfiltration of *l‐DOPA 4,5‐dioxygenase* (*DODA)* genes from *Stegnosperma halimifolium*,* Beta vulgaris*,* Carnegiea gigantea* and *Mesembryanthemum crystallinum*. The *DODA* coding sequences were cloned into multigene constructs containing the other structural genes necessary for betacyanin production (*BvCYP76AD1*,* MjcDOPA‐5GT*). The infiltration spots are labelled according to the DODA variant. For all species, the pBC‐BvDODAα1 multigene vector is included as a positive control (P). (b) The betanin content of the infiltration spots was measured using HPLC and data were represented relative to the BvDODAα1 spot present in each biological replicate. The data were combined after calculating relative amounts (RQ) for each species from individual species‐specific experiments (Supporting Information Figs S8–S11). Bars show means ± SD;* n *=* *5 for *S. halimifolium*,* B. vulgaris*,* C. gigantea* and *M. crystallinum*, except for BvDODAα2 (*n *=* *4) and BvDODAα3 (*n *=* *4). (c) A representative HPLC trace for the *DODA* *α* *1* gene from each species. The left peak is betanin and the right peak is its isomer, isobetanin. The traces are offset for presentation. ‘pLUC’ is a negative control plasmid carrying the firefly luciferase gene and ‘Betanin’ is a commercially available extract from beet hypocotyl which was used to validate the retention time of betanin in these samples.

We selected four betalain‐pigmented species that represent each of the inferred origins of betalain pigmentation, three of which are represented by complete annotated genomes: *S. halimifolium* (Stegnospermataceae), *B. vulgaris* (Amaranthaceae; Dohm *et al*., 2014), *M. crystallinum* (Aizoaceae; W. C. Yim, unpublished) and *C. gigantea* (Cactaceae; Copetti *et al*., 2017). *S. halimifolium*,* M. crystallinum* and *C. gigantea* each contain one *DODA*β and two *DODA* *α* genes, and *B. vulgaris* contains one *DODA*β and five *DODA* *α* genes (Figs S5, S6). The *DODA* genes for all four species were separately cloned into multigene vectors containing all necessary genes for betalain biosynthesis and these vectors were transiently transformed into *N. benthamiana* to test their heterologous expression (Fig. S1; Timoneda *et al*., 2018). We used the production of betacyanin in this heterologous assay as a proxy for l‐DOPA 4,5‐dioxygenase activity, with the relative strength of betacyanin production indicating the relative strength of l‐DOPA 4,5‐dioxygenase activity between loci. Upon heterologous expression, none of the *DODA*β genes from *S. halimifolium*,* B. vulgaris*,* M. crystallinum* and *C. gigantea* produced visible betacyanin pigmentation (Fig. 3a; Timoneda *et al*., 2018). Traces of betanin were detected for all loci by HPLC, but amounts were extremely low compared to the positive control, BvDODAα1 (< 0.1%; Fig. 3b).

### A single DODAα homologue in each betalain‐pigmented species exhibits high levels of l‐DOPA 4,5‐dioxygenase activity

Using the same procedure as that used to test the DODAβ enzymes, we found that only a single DODAα from each study species showed a strong production of betalain pigmentation including BvDODAα1 (Figs 3, S8–S11). All paralogues exhibiting high production of betanin in the heterologous assay (which we term as having high levels of l‐DOPA 4,5‐dioxygenase activity) are hereafter named “α1”. The amount of betanin produced by the different orthologues of DODAα1 varies relative to BvDODAα1, indicating that there may be differences in the effectiveness of these DODAα enzymes to convert l‐DOPA to betalamic acid (Fig. 3b). For example, ShDODAα1 produced a comparable amount of pigment to BvDODAα1, CgDODAα1 produced *c*. 60% as much as BvDODAα1, and McDODAα1 produced *c*. 80% more than BvDODAα1.

### Numerous DODAα homologues in both betalain‐pigmented and anthocyanin‐pigmented lineages exhibit marginal levels of l‐DOPA 4,5‐dioxygenase activity

**Figure 4:**
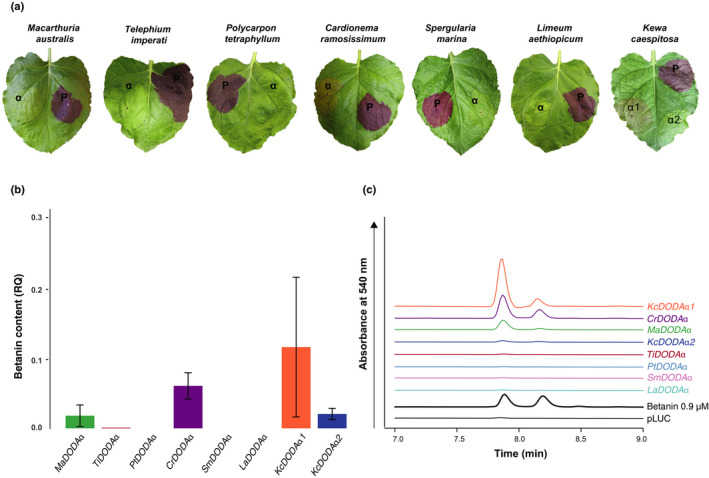
l‐DOPA 4,5‐dioxygenase activity is marginal or absent in DODA from anthocyanin‐producing taxa when heterologously expressed in *Nicotiana benthamiana*. (a) A representative leaf is shown from the agroinfiltration of *l‐DOPA 4,5‐dioxygenase* (*DODA*) genes from *Macarthuria australis* (*MaDODA* *α*), *Telephium imperati* (*TiDODA* *α*), *Polycarpon tetraphyllum* (*PtDODA* *α*), *Cardionema ramosissimum* (*CrDODA* *α*), *Spergularia marina* (*SmDODA* *α*), *Limeum aethiopicum* (*LaDODA* *α*) and *Kewa caespitosa* (*KcDODA* *α* *1*,* KcDODA* *α* *2*). The *DODA* coding sequences were cloned into multigene constructs with the other structural genes necessary for betacyanin production (*BvCYP76AD1*,* MjcDOPA‐5GT*). The pBC‐BvDODAα1 multigene vector was included as a positive control (P). (b) Betanin content of the infiltration spots was measured using HPLC. Amounts of betanin were calculated relative (RQ) to the average amount of betanin present in BvDODAα1 infiltration spots for each species and the data combined into one graph (see Supporting Information Fig. S12 for species‐specific data). Bars show means ± SD;* n* ≥ 3. (c) A representative HPLC trace for each *DODA* *α* gene from each species is shown. The left peak is betanin and the right peak is its isomer, isobetanin. The traces are offset for presentation. ‘pLUC’ is a negative control plasmid carrying the firefly luciferase gene and ‘Betanin’ is a commercially available extract from beet hypocotyl, which was used to validate the retention time of betanin in these samples.

We found that in each betalain‐pigmented species there is at least one DODAα paralogue that exhibits marginal activity (consistent with Chung *et al*., 2015 and Bean *et al*., 2018). For example, in *M. crystallinum*, pigmentation was also observed for McDODAα2, albeit at a much lower level than DODAα1 from *M. crystallinum* or any other species (Figs 3a,b, S8–S11). Depending on the strength of transient expression in particular leaves, faint pigment was also sometimes observed for BvDODAα2 and CgDODAα2, from *B. vulgaris* and *C. gigantea* respectively (Fig. 3a). Quantification of betanin in these infiltration spots showed that McDODAα2 produced *c*. 5% the amount of betanin as BvDODAα1, whereas BvDODAα2 and CgDODAα2 showed below 3% the amount of BvDODAα1 (Figs 3b, S9–S11). Despite being visually undetectable, betanin could also be detected by HPLC in ShDODAα2 and BvDODAα4 (Figs 3b, S8, S9). For two *B. vulgaris* paralogues, BvDODAα3 and BvDODAα5, betanin was undetectable (Figs 3b, S9). DODAα enzymes from anthocyanic species *M. australis* and *K. caespitosa* were also found to produce a small amount of betanin, as detected by HPLC (Figs 4, S12). MaDODAα produces *c*. 2.5% the amount of BvDODAα1, and KcDODAα1 and KcDODAα2 produce *c*. 12% and 2% of BvDODAα1, respectively. Within Caryophyllaceae, DODAα from *C. ramosissimum* and *T. imperati* produced a small amount of betanin (6% and 2.5% of BvDODAα1, respectively; Figs 4b,c, S12).

### Betalain biosynthetic genes, *DODAα1* and *CYP76AD1*, , are colocalised in Amaranthaceae but not in the representative Aizoaceae genome

**Figure 5:**
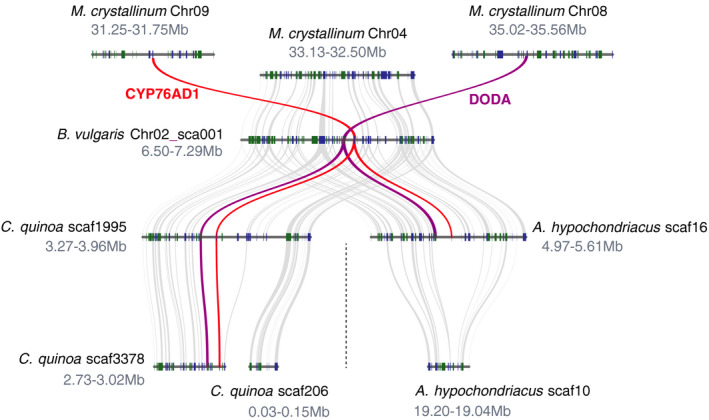
Betalain biosynthesis genes are colocalised in the genomes of three Amaranthaceae species but not in the Aizoaceae species, *Mesembryanthemum crystallinum*. Shown are the genomic regions containing the betalain biosynthetic genes, *DODA* *α* *1* and *CYP76AD1*, from *Amaranthus hypochondriacus*,* Beta vulgaris*,* Chenopodium quinoa* and *M. crystallinum*. Rectangles show predicted gene models, and blue (+ strand) and green (− strand) colours indicate relative orientations. Matching gene pairs are displayed as grey connections, and purple and red connections indicate *DODA* *α* *1* and *CYP76AD1* correspondence, respectively. Whole genome duplication is marked with a dotted line.

We previously showed that *BvDODA* *α* *1* and *BvCYP76AD1* are part of a putative gene cluster, being located close to one another (< 50 kb) and also in close linkage with the MYB that regulates both of these genes (Keller, 1936; Goldman & Austin, 2000; Hatlestad *et al*., 2014; Brockington *et al*., 2015). In *C. quinoa* and *A. hypochondriacus*, other Amaranthaceae species for which there is a genome sequence available (Yasui *et al*., 2016; Lightfoot *et al*., 2017), these genes are also colocalised (Fig. 5). However, in *M. crystallinum* in the family Aizoaceae, the locus encoding the high‐activity DODAα, *McDODA* *α* *1*, is located on a different chromosome from the *McCYP76AD1* orthologue, and the genes are not colocalised despite considerable conservation of synteny between putatively homologous chromosomes (Fig. 5). This analysis is limited to two of the four betalain‐pigmented study species because they are currently the only species for which genome assemblies are of sufficient quality to allow syntenic analysis.

### Homologues exhibiting high levels of l‐DOPA 4,5‐dioxgenase activity are not monophyletic

**Figure 6:**
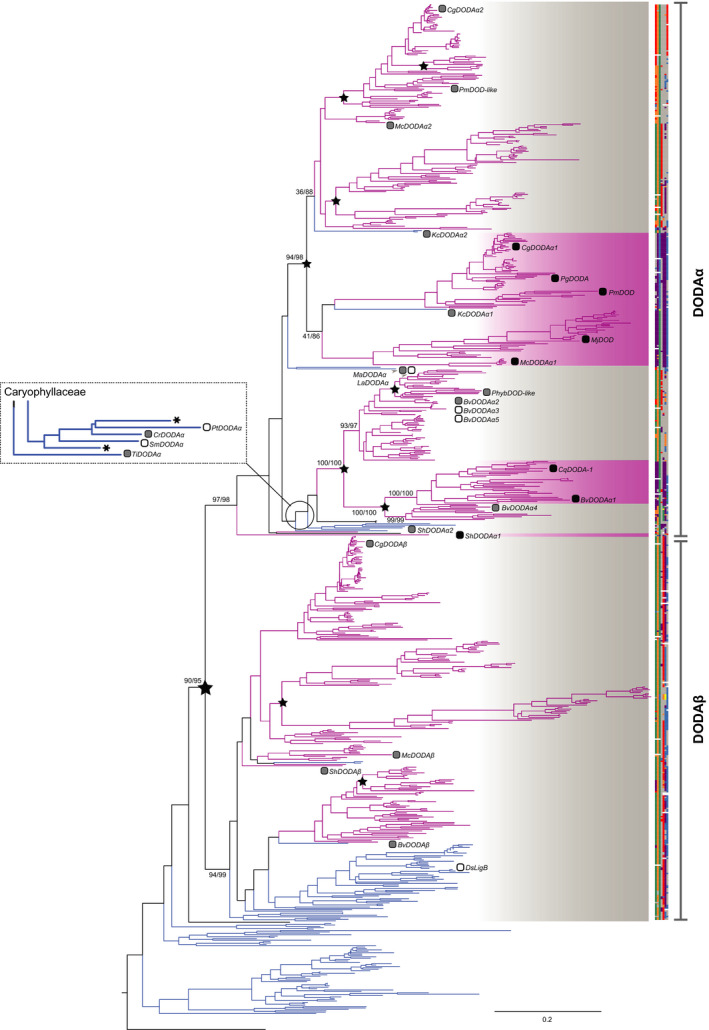
The *DODA* gene tree shows a homoplasious distribution of functionally characterised *DODA* genes that produce a high level of betalain pigments. The maximum likelihood phylogeny of Caryophyllales *l‐DOPA 4,5‐dioxygenase* (*DODA*) genes was inferred from coding sequences derived from genomes and transcriptomes. Branch lengths are expected number of substitutions per site. Scale bar gives 0.2 expected substitutions per site. Branch labels are support values for major paralogous clades from rapid bootstrapping and the SH‐like Approximate Likelihood Ratio Test, respectively, given as RBS/SH‐aLRT. Putative major duplication nodes are highlighted with stars. Branches are coloured according to the putative pigmentation state of the taxa (blue, anthocyanin; pink, betalain). Labelled tips show functionally characterised DODAs and shaded squares correspond to DODA activity (white, no activity; grey, marginal activity; black, high activity). Asterisks indicate putative pseudogenes. Annotated at tips is a colour‐coded alignment of the seven residues conferring high activity reported in Bean *et al*. (2018). *XxDODA*:* Bv*,* Beta vulgaris*;* Cg*,* Carnegiea gigantea*;* Cr*,* Cardionema ramosissimum*;* Ds*,* Dianthus superbus*;* Kc*,* Kewa caespitosa*;* La*,* Limeum aethiopicum*;* Mc*,* Mesembryanthemum crystallinum*;* Ma*,* Macarthuria australis*;* Mj*,* Mirabilis jalapa*;* Pg*,* Portulaca grandiflora*;* Phyb*,* Ptilotus* hybrid; *Pm*,* Parakeelya mirabilis*;* Pt*,* Polycarpon tetraphyllum*;* Sh*,* Stegnosperma halimifolium*;* Sm*,* Spergularia marina*;* Ti*,* Telephium imperati*.

To understand the evolution of l‐DOPA 4,5‐dioxgenase activity, we mapped our functional data to the *DODA* gene tree (Fig. 6), and also mapped functional data from studies for which DODA activity has been tested *in planta*, either in heterologous transient assays or stable transgenics, or using recombinant expression in *Escherichia coli* or yeast (Figs 3, 4; Table S6). Mapping the functional data to the tree reveals that DODAα homologues exhibiting l‐DOPA 4,5‐dioxgenase activity have a homoplastic distribution across the DODA phylogeny (Fig. 6). In total, there are three polyphyletic gene lineages containing high levels of l‐DOPA 4,5‐dioxygenase activity (*DODAα1*): a lineage specific to *Stegnosperma* singly represented by *ShDODA*α*1*, a clade arising by duplication within Amaranthaceae containing *BvDODAα1* and *CqDODA‐1*, and a clade representing the remaining betalain‐pigmented lineages, and containing *CgDODA1*,* MjDODA* *α* *1*,* McDODA* *α* *1*,* PmDOD* and *PgDODA* (Table S6). Each clade containing high‐activity DODAα paralogues is sister to clades containing marginal activity DODAα paralogues, and in the case of Amaranthaceae, the DODAα1 clade is nested within clades exhibiting no or only marginal levels of l‐DOPA 4,5‐dioxgenase activity (Fig. 6).

### Ancestral sequence reconstruction indicates that high l‐DOPA 4,5‐dioxygenase activity is a derived state within the DODAα lineage

**Figure 7:**
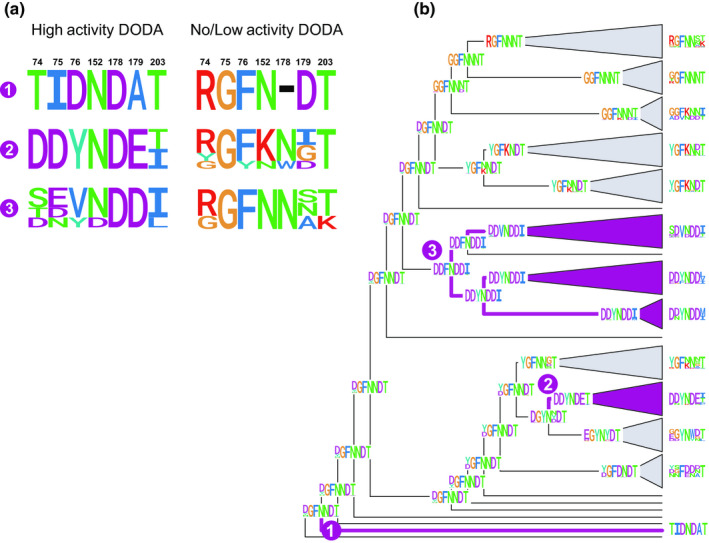
Reconstruction of seven functionally important amino acids on the l‐DOPA 4,5‐dioxygenase (DODA) phylogeny showing a shift in patterns associated with *DODA* genes of high l‐DOPA 4,5‐dioxygenase activity. (a) Proportional logo plots of the seven residues identified by Bean *et al*. (2018) across functionally characterised high activity (left) and no or only marginal activity (right) paralogues included in this study. 1: ShDODAα1 vs ShDODAα2 (Stegnospermataceae); 2: CqDODA‐1, BvDODAα1 vs PhybDODAα, BvDODAα2‐5 (Amaranthaceae); 3: McDODAα1, PmDODAα1, CgDODAα1, PgDODAα1 vs McDODAα2, PmDODAα2, CgDODAα2 (Portulacineae and Aizoaceae). Residue numbering is according to Bean *et al*. (2018). (b) Ancestral sequence reconstructions of codons by empirical Bayesian inference on a maximum likelihood phylogeny of a reduced *DODA* *α* sequence alignment. Logo plots at tips give the proportional representation of a given amino acid across all sequences in each collapsed clade for each of the sites identified by Bean *et al*. (2018). Numbers at nodes match the ancestor of the high‐activity paralogues summarised in (a).

A recent study characterised seven residues that are important for l‐DOPA 4,5‐dioxygenase activity (Bean *et al*., 2018). We carried out ancestral sequence reconstruction using coding sequences on a reduced *DODA* *α* gene tree (Figs S13, S14) and observed that the three putative origins of highly active DODAα are marked by three convergent shifts in these seven residues (Figs 6, 7), which occur post‐gene duplication. For the residues inferred for the clade containing the *M. crystallinum* and *C. gigantea* highly active DODAα (DDFNDDI; Fig. 7), four out of seven of the predicted residues are identical to those inferred for the Amaranthaceae highly active DODAα clade (DDYNDET). For *Stegnosperma*, the motif is more divergent, with three residues identical to the Amaranthaceae betalain‐producing DODAα clade and two residues identical to the clade containing the *M. crystallinum* and *C. gigantea* highly active DODAα. The highly active DODAα are derived from ancestral nodes with inferred motifs that share more similarity with the marginal or no activity DODAα lineages (XGFNN[N/D]T), and this motif is highly conserved across the backbone and represented at almost all ancestral nodes (Figs 6, 7). Amino acid reconstruction gave similar results (Figs S15, S16).

## Discussion

### Polyphyletic patterns of elevated l‐DOPA 4,5‐dioxygenase activity support the recurrent specialisation to betalain pigmentation

Since our earlier ancestral state pigment reconstructions (Brockington *et al*., 2011, 2015), phylogenomic data have advanced new hypotheses for relationships within Caryophyllales (Walker *et al*., 2018) and the previously uncharacterised Limeaceae and Simmondsiaceae have been shown to be anthocyanic (Thulin *et al*., 2016). Here, we explicitly account for the large number of taxa for which pigmentation status is unknown and constrain our phylogenetic hypothesis to match the latest phylogenomic study of Walker *et al*. (2018), which places betalain‐pigmented Stegnospermataceae as sister to anthocyanic Macarthuriaceae. In the context of these new data (Fig. 2), our reconstructions do not infer a single evolution of betalains, as previously suggested (Brockington *et al*., 2015), but rather imply up to four separate origins of betalain pigmentation in Caryophyllales.

To explore the hypothesis of multiple origins of betalain pigmentation suggested by our reconstructions, we then selected four species that represent each of the putative origins: *S. halimifolium*,* B. vulgaris*,* M. crystallinum* and *C. gigantea*. Using heterologous transient assays, we inferred relative levels of l‐DOPA 4,5‐dioxygenase activity based on the proxy of betanin production (Fig. 3). Our data show that activity is barely detectable in *DODA*β loci from betalain‐pigmented species, supporting our original hypothesis that the high levels of l‐DOPA 4,5‐dioxygenase activity evolved exclusively within the DODAα lineage (Brockington *et al*., 2015). However, although betalain‐pigmented species have multiple paralogues of *DODA*α genes, *only one* of these DODAα in each species encodes a protein which exhibits high levels of l‐DOPA 4,5‐dioxygenase activity. All betalain‐pigmented species also exhibit DODAα paralogues with no or only marginal l‐DOPA 4,5‐dioxygenase activity and we also detected marginal activity in several anthocyanic taxa (Fig. 4). Thus, marginal activity is widespread among Caryophyllales DODAα enzymes, suggesting broader underlying catalytic promiscuity in the DODAα lineage. Catalytic promiscuity is well documented in the broader protocatechuate dioxygenase gene family of which the LigB/DODA lineage is a member (Burroughs *et al*., 2019). Such promiscuity is an important feature of metabolic evolution, potentially conferring evolvability (Weng & Noel, 2012; Leong & Last, 2017), and may have significant implications for the recurrent evolution of betalain pigmentation, as discussed below.

Previous analysis of the genome of *B. vulgaris* revealed a putative ‘gene cluster’ (*sensu* Osbourn, 2010) in which *DODA*α and *CYP76AD1* are colocalised in chromosome 2 (Brockington *et al*., 2015). The *B. vulgaris* locus with high levels of l‐DOPA 4,5‐dioxygenase activity falls within this operon, while the paralogues with no or only marginal l‐DOPA 4,5‐dioxygenase activity occur outside of the gene cluster, supporting the concept of a betalain gene cluster in *B. vulgaris*. Our analysis, which describes the relative synteny of homologous loci between genomes, also shows that the betalain gene cluster appears to be conserved in the genomes of *C. quinoa* and *A. hypochondriacus* (Fig. 5). These divergent species all belong to the family Amaranthaceae and represent one putative origin of betalain pigmentation (Fig. 2, origin no. 2). However, *CYP76AD1* and *DODA*α*1* are not clustered in the genome of *M. crystallinum*, which represents a different putative origin (Fig. 2, origin no. 3). Therefore, the absence of colocalisation in *M. crystallinum* highlights interesting structural genomic differences among putative betalain origins.

Mapping loci encoding functionally characterised DODA proteins to a comprehensively taxon‐sampled *DODA* gene tree, we found that within the DODAα lineage, loci encoding proteins with high levels of l‐DOPA 4,5‐dioxygenase activity are not monophyletic (Fig. 6). Specifically, each clade containing high‐activity DODAα paralogues is sister to clades containing marginal activity DODAα, suggesting polyphyletic origins of high activity, associated with gene duplication *within* the DODAα lineage. Previous research identified residues in seven sites which were necessary and sufficient to confer higher levels of l‐DOPA 4,5‐dioxygenase activity (Bean *et al*., 2018). Phylogenetic reconstruction of these seven residues across the DODAα clade showed that polyphyletic clades containing proteins with high activity have distinctive motifs for these seven residues (Figs 6, 7). Furthermore, motifs associated with high activity evolved at least three times from a background of motifs more similar to those proteins with no or marginal l‐DOPA 4,5‐dioxygenase activity. The diversity we recognise at these motifs in high‐activity DODAα sequences (e.g. between BvDODAα1 and ShDODAα1) indicates that high activity may arise from divergent sequence motifs and represent molecular convergence at key functional residues.

**Figure 8:**
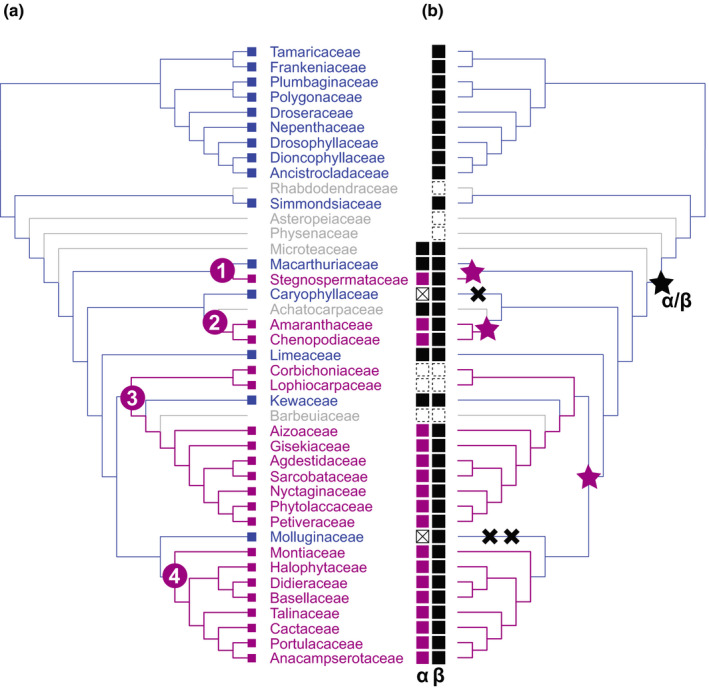
Summary of the major evolutionary changes inferred with respect to transitions in pigment type and corresponding transitions in l‐DOPA 4,5‐dioxygenase activity. (a) Simplified pigment reconstruction shows family‐level relationships with numbers in purple circles representing the four inferred origins of betalain pigmentation. Tips are coloured according to pigmentation state: anthocyanin (blue), betalain (pink) and unknown (grey). (b) Mirror image of pigment reconstruction topology. A black star marks the initial *DODA* *α*/*DODA* *β* duplication. Purple stars indicate inferred phylogenetic locations of *DODA* *α* gene duplications giving rise to paralogues with high l‐DOPA 4,5‐dioxygenase activity. Black crosses indicate loss of a DODAα paralogue. Tips show inferred presence or absence of *DODA* *α*/*DODA* *β*: black squares indicate presence of *DODA* *α*/*DODA* *β*, white squares with a cross indicate loss of *DODA* *α*, and white squares with a dashed boundary indicate missing data; DODAα squares coloured purple are inferred to have high *L*‐DOPA 4,5‐dioxygenase activity.

Intriguingly, the origins of high l‐DOPA 4,5‐dioxygenase activity following gene duplication, and associated residue shifts, are congruent with at least three of the four origins of betalain pigmentation inferred from our pigment reconstructions (Fig. 8). On the basis of these integrated observations we argue that betalain biosynthesis evolved multiple times in concert with recurrent gene duplication and neofunctionalisation *within* the DODAα clade, rather than as a single event at *the base* of the DODAα clade. Specifically, data in support of this model include: a background of marginal levels of l‐DOPA 4,5‐dioxygenase activity implying inherent evolvability of the ancestral enzyme (see following paragraph); polyphyletic origins of high l‐DOPA 4,5‐dioxygenase activity coincident with multiple inferred origins of betalain pigmentation; and derived and convergent shifts in key residues underpinning high l‐DOPA 4,5‐dioxygenase activity. Together, this model explains and conceptually links the homoplastic distribution of betalain lineages and the high levels of gene duplication observed in the DODAα clade.

Polyphyletic origins of betalain pigmentation occur only within Caryophyllales (Fig. 2), while polyphyletic origins of high l‐DOPA 4,5‐dioxygenase activity are constrained to the Caryophyllales‐specific DODAα clade (Fig. 6). Both patterns link to the concept of evolutionary precursors in which underlying evolutionary state(s) potentiate recurrent evolution of subsequent complex traits (*sensu* Marazzi *et al*., 2012). In this scenario, we propose that an initial precursor step was the evolution of tyrosine hydroxylase activity by duplication in the CYP76AD lineage, leading to an abundance of l‐DOPA (DeLoache *et al*., 2015; Polturak *et al*., 2016; Sunnadeniya *et al*., 2016), the necessary substrate for betalamic acid biosynthesis. Given an abundance of l‐DOPA, a subsequent precursor step was duplication in the DODA lineage that gave rise to a clade of DODAα enzymes, whose ancestral function is currently unknown, but presumably with some promiscuous ability to act on l‐DOPA to produce trace betalamic acid. Subsequently, further repeated duplication within the DODAα lineage led to recurrent neofunctionalisation towards high levels of l‐DOPA 4,5‐dioxygenase activity and the production of betalamic acid.

Such a model, in which similar enzymatic function has arisen repeatedly from homologous but nonorthologous enzymes, is not unprecedented. Many studies indicate that this form of convergent evolution is widespread in specialised plant metabolism (Pichersky & Lewinsohn, 2011). For example, the enzymes that methylate purine intermediates in caffeine biosynthesis in *Coffea* vs *Thea* evolved from different branches of the SABATH carboxyl methyltransferase family (Yoneyama *et al*., 2006); and disparate origins of pyrrolizidine alkaloids have arisen through recurrent evolution of homospermidine synthase from the ubiquitous enzyme deoxyhypusine synthase (Reimann *et al*., 2004). However, the detection of this same phenomenon in the evolution of betalains is perhaps surprising, because the retention of enzymes for anthocyanin biosynthesis in betalain‐pigmented species (Shimada *et al*., 2004, 2005) has encouraged the assumption that reversals to anthocyanin are more likely than multiple shifts to betalain pigmentation (Brockington *et al*., 2011, 2015).

### Reconciling polyphyletic origins of high l‐DOPA 4,5‐dioxygenase activity with *DODAα* gene loss

It has always been striking that each major betalain‐pigmentated clade is subtended at, or towards, its base by an anthocyanic lineage (Brockington *et al*., 2011). Given this essential pattern, our trait reconstructions suggest multiple origins of betalain pigmentation. In turn, this implies that many lineages are anthocyanic through retention of an ancestral state, rather than through reversal from betalain pigmentation (Brockington *et al*., 2011; Fig. 2). Yet, we earlier reported that *DODA* *α* are lost or down‐regulated in anthocyanic Caryophyllaceae and Molluginaceae, and previously argued that loss of *DODA* *α* in these anthocyanic lineages is consistent with reversals from betalain pigmentation to anthocyanin pigmentation (Brockington *et al*., 2015). However, the emerging picture is more complex, and with new data presented here, we find: evidence of *DODA* *α* loci in all but one of the anthocyanic lineages in Caryophyllales; evidence of *DODA* *α* gene loss in Caryophyllaceae and Molluginaceae, but no evidence of *DODA* *α* gene loss in the anthocyanin‐pigmented lineages, *K. caespitosa*,* M. australis* and *L. aethiopicum*; and evidence of *DODA* *α* loci with marginal l‐DOPA 4,5‐dioxygenase activity within anthocyanic *M. australis*,* K. caespitosa*,* C. ramosissimum* and *T. imperati*. Clearly, evolutionarily disparate anthocyanic lineages show different patterns of molecular evolution with respect to *DODA* *α*. Given this complex evolutionary milieu, and in the face of compelling evidence for polyphyletic origins of elevated l‐DOPA 4,5‐dioxygenase activity, below we propose two alternative hypotheses to explain loss of *DODA* *α* loci in anthocyanic lineages.

1. The patterns we detect may suggest lability in early stages of betalain evolution. Anthocyanins and betalains have never been found to co‐occur, but it is possible that the two classes of pigments did co‐occur in early evolutionary stages. In this scenario, repeated evolution of increased l‐DOPA 4,5‐dioxygenase activity allowed for betalain pigmentation, initially co‐occurring with anthocyanins. However, evolution of an integrated betalain pathway requires more than biosynthetic enzymes (e.g. the recruitment of the MYB transcriptional regulators; Hatlestad *et al*., 2014). Therefore, establishment and enhancement of the betalain pathway was only achieved in certain lineages, those which specialised to betalain pigmentation. By contrast, other lineages arising close to the origins of elevated l‐DOPA 4,5‐dioxygenase activity specialised to anthocyanins rather than betalains, ultimately losing the DODAα paralogues with elevated l‐DOPA 4,5‐dioxygenase activity. In these cases, anthocyanic lineages may have retained anthocyanins from ancestors in which both pigments coexisted, and inferences of reversals to anthocyanins based on *DODA* *α* loss are misleading. This scenario is appealing because we do not see any anthocyanin lineages that are nested deeply within the betalain‐pigmented clades that stem from each inferred origin (Fig. 2), and so shifts to anthocyanin pigmentation seem less likely with evolutionary distance from inferred origins of betalain pigmentation.
2. Previously, Lopez‐Nieves *et al*. (2018) speculated that the evolution of tyrosine‐derived betalains occurred in a metabolic environment enriched for tyrosine. The arogenate dehydrogenase enzyme (ADHα) responsible for increased tyrosine availability is also lost and/or down‐regulated in the anthocyanic Caryophyllaceae and Molluginaceae lineages (Lopez‐Nieves *et al*., 2018). Therefore, the shift to anthocyanins in these taxa may indicate deeper shifts away from a tyrosine‐rich metabolism towards a metabolism in which phenylalanine plays a canonical role. The primary function of the DODAα paralogues with marginal l‐DOPA 4,5‐dioxygenase activity is unknown, but may catalyse production of tyrosine‐derived metabolites, other than betalains. In this scenario, the duplication that gave rise to the DODAβ and DODAα lineages led to neofunctionalisation in the DODAα lineage towards an unknown but tyrosine‐derived enzymatic activity, with marginal l‐DOPA 4,5‐dioxygenase activity. Loss of *DODA* *α* in Caryophyllaceae and Molluginaceae could instead reflect loss of the unknown tyrosine‐derived enzymatic activity, in the context of shifts towards more phenylalanine‐biased metabolism, independent of origins of high l‐DOPA 4,5‐dioxygenase activity.

### Conclusion

The evolutionary origin of betalain pigments in Caryophyllales and the processes that led to their homoplastic distribution have been the subject of much debate. Our new data and analyses offer compelling evidence for recurrent specialisation to betalain biosynthesis. Specifically: a background of marginal levels of l‐DOPA 4,5‐dioxygenase activity implying inherent evolvability; polyphyletic origins of high l‐DOPA 4,5‐dioxygenase activity coincident with multiple inferred origins of betalain pigmentation; derived and convergent shifts in key residues underpinning high l‐DOPA 4,5‐dioxygenase activity; and a lack of conservation of the betalain metabolic gene cluster between putative origins of betalain pigmentation. However, our hypothesis requires future experimentation. First, it will be important to identify the primary function of those DODAα proteins that only exhibit marginal l‐DOPA 4,5‐dioxygenase activity. Elucidation of this unknown function will further inform the inferences made in this study and direct future hypotheses. Further validation could also emerge by considering other aspects of the betalain biosynthesis pathway. For example, as exemplified by our syntenic analyses, it may be possible to discern the signal of multiple origins at different hierarchical levels of the betalain pathway, including: patterns of gene clustering by genomic colocalisation; patterns of co‐option of transcriptional regulators and other genes; and the dissection of the molecular convergence signal at key residues. It is fortunate here that three of the putative origins of betalain biosynthesis are represented by well‐resourced experimental systems: *Portulaca grandiflora*,* Mirabilis jalapa* and *B. vulgaris*, which together promise rapid progress in this era of betalain renaissance.

## Author contributions

SFB, HS, TF and NWH planned and designed the research; SFB, HS and NWH wrote the manuscript; HS, TF, NWH, SLN, BP, RG, WCY, AT, RB, MJS and SAS performed experiments and analysed the data; MJM, HT, LZ, SAS, WCY, MJS, DC and JCC provided sequence data. All authors read and approved the manuscript. HS, TF and NW‐H contributed equally to this work.

## Supporting information

Please note: Wiley Blackwell are not responsible for the content or functionality of any Supporting Information supplied by the authors. Any queries (other than missing material) should be directed to the *New Phytologist* Central Office.

**Fig. S1** Taxon‐labelled *DODA* *α* gene tree with nucleotide branch lengths and support values.
**Fig. S2** Schematic of binary vectors used in this study.
**Fig. S3** Maximum likelihood, time‐calibrated genus‐level phylogeny of Caryophyllales, inferred from seven nuclear and plastid markers.
**Fig. S4** Ancestral state reconstruction of pigments by maximum likelihood and Bayesian inference via stochastic mapping.
**Fig. S5** A simplified representation of the *DODA* gene tree.
**Fig. S6** Taxon‐labelled maximum likelihood gene tree of *DODA* in Caryophyllales with support values.
**Fig. S7** The *Corrigiola litoralis* and *Pollichia campestris DODA* *α* genes are putative pseudogenes.
**Fig. S8** Functional characterisation of the full complement of *DODA* genes from *Stegnosperma halimifolium*.
**Fig. S9** Functional characterisation of the full complement of *DODA* genes from *Beta vulgaris*.
**Fig. S10** Functional characterisation of the full complement of *DODA* genes from *Mesembryanthemum crystallinum*.
**Fig. S11** Functional characterisation of the full complement of *DODA* genes from *Carnegiea gigantea*.
**Fig. S12** Functional characterisation of *DODA* *α* genes from anthocyanic Caryophyllales taxa.
**Fig. S13** Taxon‐labelled subsampled maximum likelihood *DODA* *α* gene tree with nucleotide branch lengths and support values.
**Fig. S14** Taxon‐labelled subsampled maximum likelihood *DODA* *α* gene tree with codon branch lengths.
**Fig. S15** Taxon‐labelled subsampled maximum likelihood *DODA* *α* gene tree with amino acid branch lengths.
**Fig. S16** Ancestral amino acid sequence reconstruction for seven residues on subsampled *DODA* *α* gene tree.
**Methods S1** Genome assembly.
**Methods S2** Identification of full complements of *DODA* genes from betalain‐pigmented species for functional analysis.
**Methods S3** Phylogeny of Caryophyllales.
**Methods S4** Pigment reconstruction.
**Methods S5** Gene tree of Caryophyllales *DODA* genes.
**Methods S6** Ancestral sequence reconstruction.
**Table S1** Oligonucleotide primers used in this study.
**Table S2** Details of *DODA* genes functionally characterised in this study.

**Table S3** Pigmentation status of 174 genera of Caryophyllales gathered from the literature.
**Table S4** Genome and transcriptome assemblies used in this study.
**Table S5** *DODA* sequences used in *DODA* phylogeny.
**Table S6** Details of *DODA* genes that have been functionally characterised from Caryophyllales species and mapped to the *DODA* gene tree.
