# Supplementary material for: Evolution of l‐DOPA 4,5‐dioxygenase activity allows for recurrent specialisation to betalain pigmentation in Caryophyllales
Source: New Phytol. 2019 Sep 29;227(3):914–29. doi: 10.1111/nph.16089 (PMC7384185; doi:10.1111/nph.16089)
Supplement: Supplementary file 1 — Fig. S1 Taxon‐labelled DODA α gene tree with nucleotide branch lengths and support values. Fig. S2 Schematic of binary vectors used in this study. Fig. S3 Maximum likelihood, time‐calibrated genus‐level phylogeny of Caryophyllales, inferred from seven nuclear and plastid markers. Fig. S4 Ancestral state reconstruction of pigments by maximum likelihood and Bayesian inference via stochastic mapping. Fig. S5 A simplified representation of the DODA gene tree. Fig. S6 Taxon‐labelled maximum likelihood gene tree of DODA in Caryophyllales with support values. Fig. S7 The Corrigiola litoralis and Pollichia campestris DODA α genes are putative pseudogenes. Fig. S8 Functional characterisation of the full complement of DODA genes from Stegnosperma halimifolium. Fig. S9 Functional characterisation of the full complement of DODA genes from Beta vulgaris. Fig. S10 Functional characterisation of the full complement of DODA genes from Mesembryanthemum crystallinum. Fig. S11 Functional characterisation of the full complement of DODA genes from Carnegiea gigantea. Fig. S12 Functional characterisation of DODA α genes from anthocyanic Caryophyllales taxa. Fig. S13 Taxon‐labelled subsampled maximum likelihood DODA α gene tree with nucleotide branch lengths and support values. Fig. S14 Taxon‐labelled subsampled maximum likelihood DODA α gene tree with codon branch lengths. Fig. S15 Taxon‐labelled subsampled maximum likelihood DODA α gene tree with amino acid branch lengths. Fig. S16 Ancestral amino acid sequence reconstruction for seven residues on subsampled DODA α gene tree. Methods S1 Genome assembly. Methods S2 Identification of full complements of DODA genes from betalain‐pigmented species for functional analysis. Methods S3 Phylogeny of Caryophyllales. Methods S4 Pigment reconstruction. Methods S5 Gene tree of Caryophyllales DODA genes. Methods S6 Ancestral sequence reconstruction. Table S1 Oligonucleotide primers used in this study. Table S2 Details of DODA genes functionally char [file NPH-227-914-s001.pdf]

***New Phytologist* Supporting Information**

Article title: Evolution of *L*-DOPA 4,5-dioxygenase activity allows for recurrent specialisation to betalain pigmentation in Caryophyllales

Authors: Hester Sheehan*, Tao Feng*, Nathanael Walker-Hale*, Samuel Lopez-Nieves, Boas Pucker, Rui Guo, Won C. Yim, Roshani Badgami, Alfonso Timoneda, Lijun Zhao, Helene Tiley, Dario Copetti, Michael J. Sanderson, John Cushman, Michael J. Moore, Stephen A. Smith, Samuel F. Brockington

Article acceptance date: 22 July 2019

The following Supporting Information is available for this article:

**Fig. S1** Taxon-labeled *DODAα* gene tree with nucleotide branch lengths and support values (**pdf**)
**Fig. S2** Schematic of binary vectors used in this study
**Fig. S3** Maximum likelihood, time-calibrated genus-level phylogeny of Caryophyllales, inferred from seven nuclear and plastid markers (**pdf**)
**Fig. S4** Ancestral state reconstruction of pigments by maximum likelihood and Bayesian inference via stochastic mapping.
**Fig. S5** A simplified representation of the *DODA* gene tree
**Fig. S6** Taxon-labeled maximum likelihood gene tree of *DODA* in Caryophyllales with support values (**pdf**)
**Fig. S7** The *Corrigiola litoralis* and *Pollichia campestris DODAα* genes are putative pseudogenes
**Fig. S8** Functional characterisation of the full complement of *DODA* genes from *Stegnosperma halimifolium*
**Fig. S9** Functional characterisation of the full complement of *DODA* genes from *Beta vulgaris*
**Fig. S10** Functional characterisation of the full complement of *DODA* genes from *Mesembryanthemum crystallinum*
**Fig. S11** Functional characterisation of the full complement of *DODA* genes from *Carnegiea gigantea*
**Fig. S12** Functional characterisation of *DODAα* genes from anthocyanic Caryophyllales taxa
**Fig. S13** Taxon-labeled subsampled maximum likelihood *DODAα* gene tree with nucleotide branch lengths and support values (**pdf**)
**Fig. S14** Taxon-labeled subsampled maximum likelihood *DODAα* gene tree with codon branch lengths (**pdf**)
**Fig. S15** Taxon-labeled subsampled maximum likelihood *DODAα* gene tree with amino acid branch lengths (**pdf**)
**Fig. S16** Ancestral amino acid sequence reconstruction for seven residues on subsampled *DODAα* gene tree

**Table S1** Oligonucleotide primers used in this study
**Table S2** Details of *DODA* genes functionally characterised in this study
**Table S3** Pigmentation status of 174 genera of Caryophyllales gathered from the literature (**xls**)
**Table S4** Genome and transcriptome assemblies used in this study (**xls**)
**Table S5** *DODA* sequences used in *DODA* phylogeny (**xls**)
**Table S6** Details of *DODA* genes that have been functionally characterised from Caryophyllales species and mapped to the *DODA* gene tree

**Methods S1** Genome assembly
**Methods S2** Identification of full complements of *DODA* genes from betalain-pigmented species for functional analysis
**Methods S3** Phylogeny of Caryophyllales
**Methods S4** Pigment reconstruction
**Methods S5** Gene tree of Caryophyllales *DODA* genes
**Methods S6** Ancestral Sequence Reconstruction

**Figure S1** Taxon-labeled maximum likelihood *DODAα* gene tree with nucleotide branch lengths and support values. Sequences in a strongly supported *DODAα* clade from the full gene tree (Figs. **6**, **S6**) were extracted and a tree with support values was inferred with RAxML. Branch values are support from 200 rapid bootstrap replicates and the SH-aLRT, respectively (RBS/SH-aLRT). Branch lengths are expected substitutions per site (**pdf**).

**Figure S2** Schematic of multi-gene binary vectors used in this study. *DODA* (pink text) was situated in second position and was the only element that varied between vectors. 35S, Cauliflower mosaic virus 35S promoter/terminator; act2, *Arabidopsis thaliana actin2* terminator; cDOPA-5GT, *Mirabilis jalapa cyclo-DOPA 5-O-glucosyltransferase*; CYP76AD1, *Beta vulgaris CYP76AD1*; DODA, *L*-DOPA 4,5-dioxygenase; Luciferase, *Photinus pyralis luciferase*; nos, *nopaline synthase* promoter/terminator; Ub10, *A. thaliana ubiquitin10* promoter.

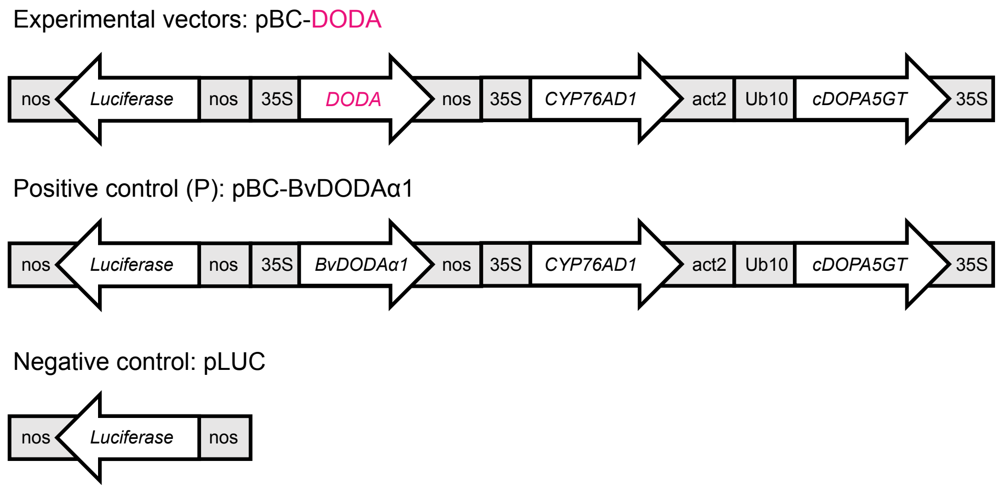


**Figure S3** Maximum likelihood, time-calibrated genus-level phylogeny of Caryophyllales, inferred from seven nuclear and plastid markers. Sequences were collected using PyPHLAWD and a tree was inferred using RAxML. Branch lengths were scaled to time using treePL with fossil and secondary constraints. Branch labels give the rapid bootstrap support over 100 replicates, while node labels give the inferred node age in millions of years (**pdf**).

**Figure S4** Ancestral state reconstruction of pigments by maximum likelihood and Bayesian inference via stochastic mapping. Tips are coloured according to pigmentation state: anthocyanin (blue), betalain (pink) and unknown (grey). Numbered arrows between states (A: anthocyanin, B: betalain) give the inferred rate of transition (myr-1) for maximum likelihood inference or the number of transitions between states on average across $n$ = 1000 stochastic maps. Pie charts at nodes give the marginal scaled likelihood of each state for maximum likelihood inference or the posterior probability for Bayesian inference: **(a)** ancestral state reconstruction by Bayesian inference under the ER model; **(b)** ancestral state reconstruction by Bayesian inference under the ARD model; **(c)** ancestral state reconstruction by maximum likelihood under the (asymmetric) ER model; **(d)** ancestral state reconstruction by maximum likelihood under the (asymmetric) ARD model.

a)

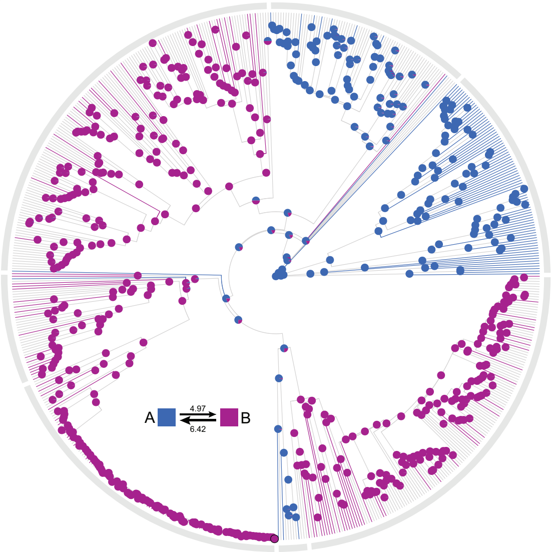


b)

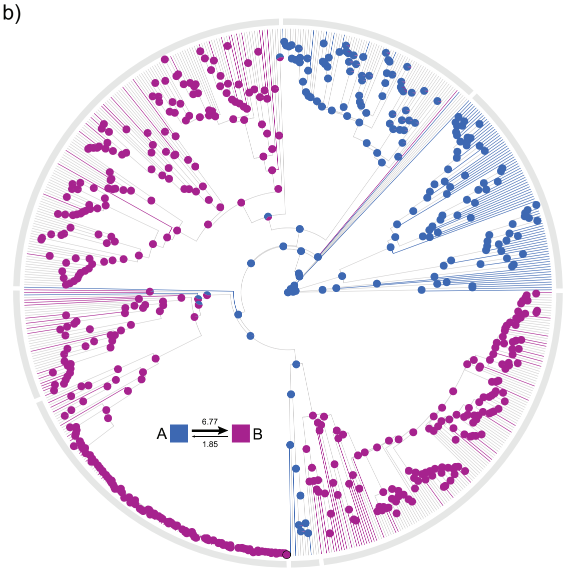


c)

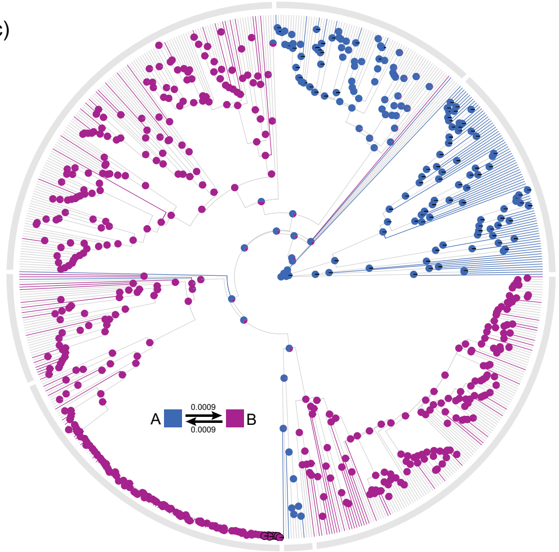


d)

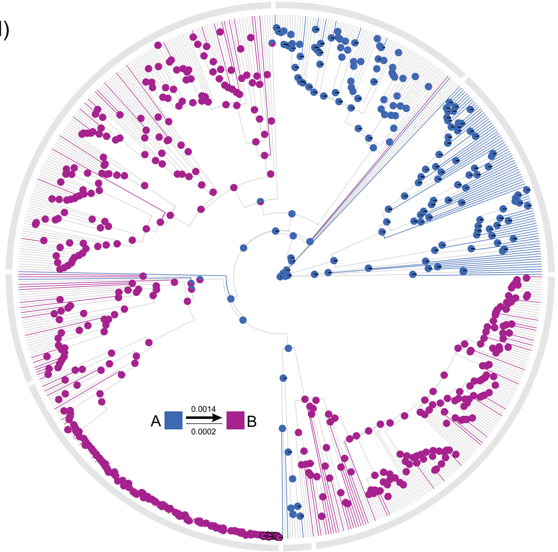

**Figure S5** A simplified representation of the *DODA* gene tree. A maximum likelihood phylogeny of Caryophyllales *DODA* genes inferred from coding sequences derived from genome and transcriptome assemblies. Branch lengths are expected number of substitutions per site. Scale bar gives 0.2 expected substitutions per site. Branch labels are support values for major paralogous clades from rapid bootstrapping and the SH-like Approximate Likelihood Ratio Test, respectively, given as RBS/SH-aLRT. Putative major duplication nodes are highlighted with stars. Branches are coloured according to the putative pigmentation state of the taxa (blue = anthocyanin, pink = betalain).

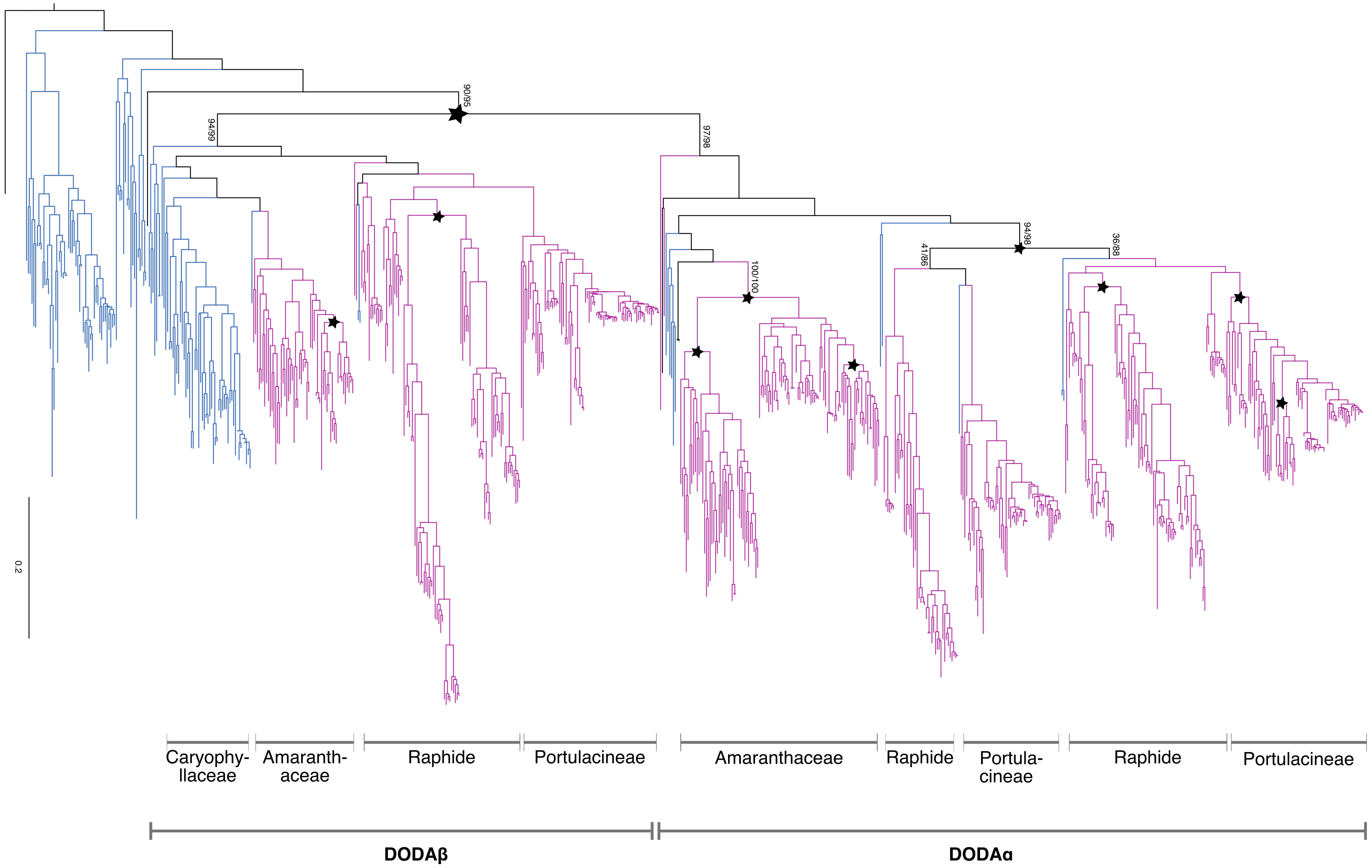
90/95
94/99
97/98
100/100
94/98
41/86
36/88
0.2
Caryophy-llaceae
Amaranth-aceae
Raphide
Portulacineae
Amaranthaceae
Raphide
Portula-cineae
Raphide
Portulacineae
DODAβ
DODAα

**Figure S6** Taxon-labeled maximum likelihood gene tree of *DODA* in Caryophyllales with support values. Sequences were collected from genome and transcriptome assemblies using a baited search approach and a tree with support values was inferred using RAxML. Branch labels are support from 200 rapid bootstrap replicates and SH-aLRT, respectively (RBS/SH-aLRT). Branch lengths are expected substitutions per site (**pdf**).

**Figure S7** The *Corrigiola litoralis* and *Pollichia campestris DODAα* genes are putative pseudogenes. A schematic representation of the *DODAα* genes in *Corrigiola litoralis* and *Pollichia campestris* showing the mutations that affect their reading frame and introduce premature stop codons. *BvDODAα1* is included for comparison.

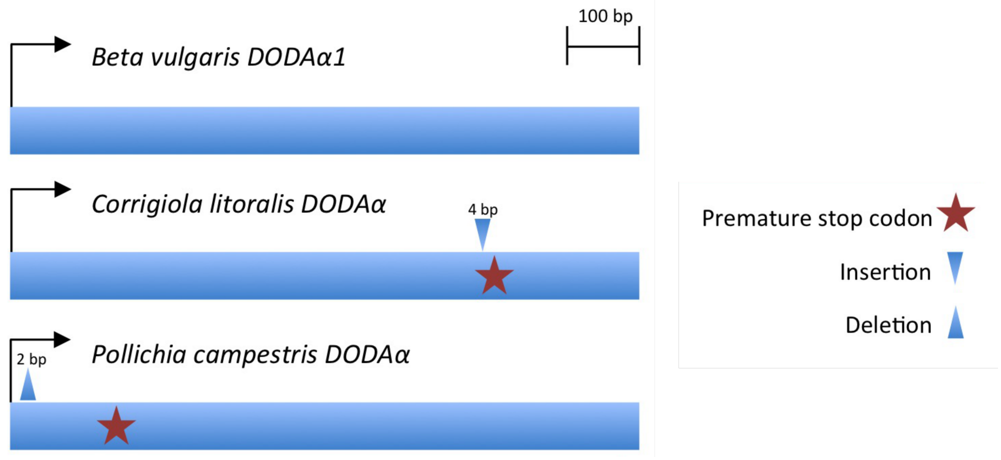

**Figure S8** Functional characterisation of the full complement of *DODA* genes from *Stegnosperma halimifolium*. **(a)** Representative *Nicotiana bethamiana* leaves are shown from the agrobacterium infiltration of *DODA* genes from *S. halimifolium*. The *DODA* coding sequences were cloned into multi-gene constructs containing the other structural genes necessary for betacyanin production (*BvCYP76AD1*, *MjcDOPA-5GT*). The infiltration spots are labelled according to the DODA variant. The vector, pBC-BvDODAα1, was included as a positive control (P). pLUC was used as a negative control. **(b)** Betanin quantification in each of the leaves infiltrated with the *S. halimifolium DODA* genes and the pBC-BvDODAα1 positive control. "ShA3" corresponds to the leaf shown in **(a)**. **(c)** A representative HPLC trace is shown for each spot from the "ShA3" replicate. An external betanin standard (Betanin) was used to validate the retention time of betanin in these samples and calculate absolute amounts of betanin. The left peak is betanin and the right peak is its isomer, isobetanin.

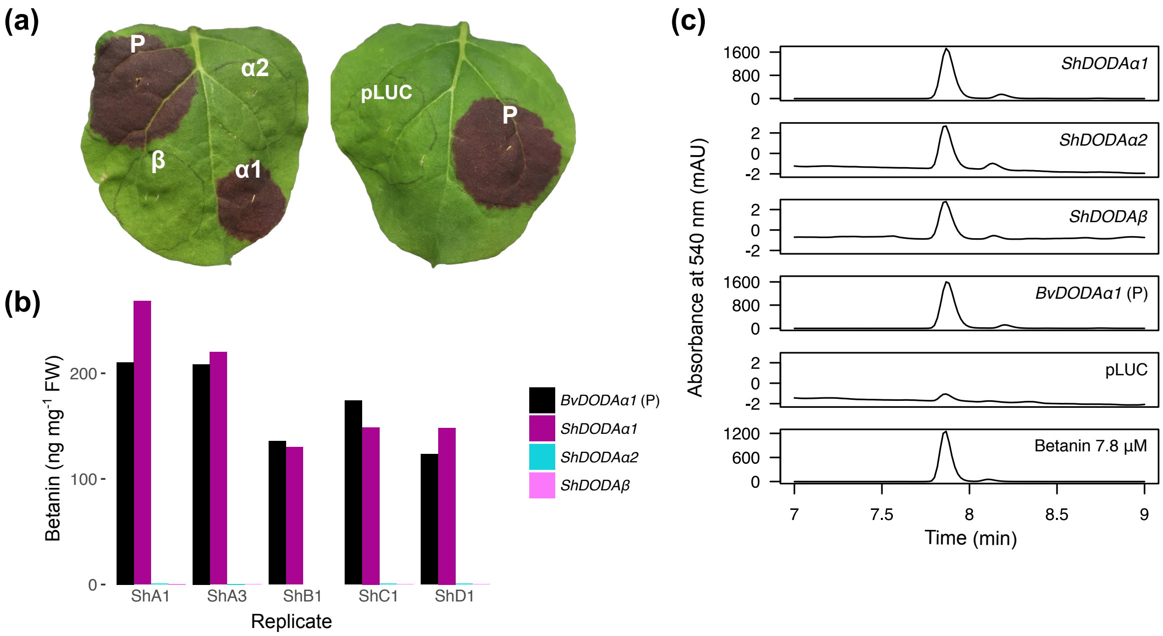

**Figure S9** Functional characterisation of the full complement of *DODA* genes from *Beta vulgaris*.
**(a)** Representative *Nicotiana bethamiana* leaves are shown from the agrobacterium infiltration of *DODA* genes from *B. vulgaris*. The *DODA* coding sequences were cloned into multi-gene constructs containing the other structural genes necessary for betacyanin production (*BvCYP76AD1*, *MjcDOPA-5GT*). The infiltration spots are labelled according to the DODA variant. BvDODAα1 is represented by (P) for positive control which it served as throughout the study. pLUC was used as a negative control. nd, no data. **(b)** Betanin quantification in each of the leaves infiltrated with the *B. vulgaris DODA* genes. “25c2”, “45c2” and “Bb1” correspond to the leaves shown in **(a)**. **(c)** A representative HPLC trace is shown for each spot of the samples shown in **(a)**. An external betanin standard (Betanin) was used to validate the retention time of betanin in these samples and calculate absolute amounts of betanin. The left peak is betanin and the right peak is its isomer, isobetanin.

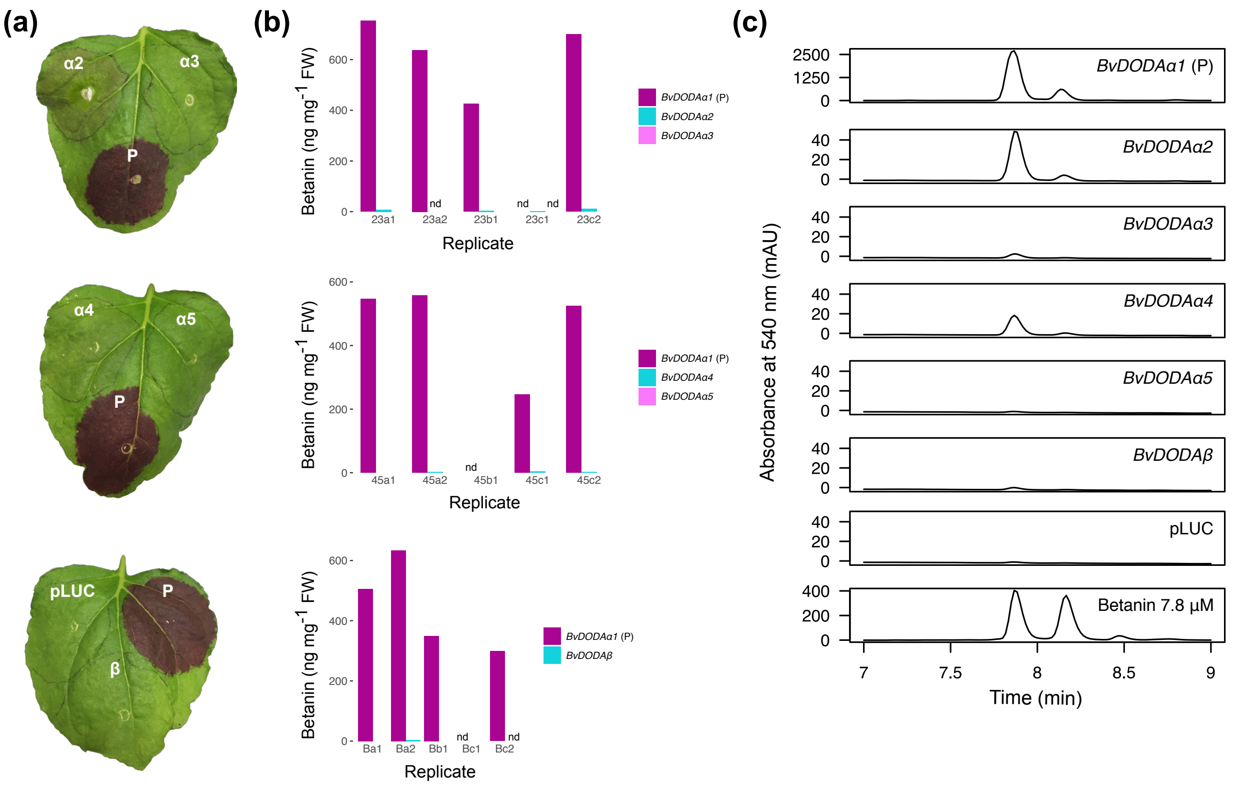

**Figure S10** Functional characterisation of the full complement of *DODA* genes from *Mesembryanthemum crystallinum*. **(a)** Representative *Nicotiana bethamiana* leaves are shown from the agrobacterium infiltration of *DODA* genes from *M. crystallinum*. The *DODA* coding sequences were cloned into multi-gene constructs containing the other structural genes necessary for betacyanin production (*BvCYP76AD1*, *MjcDOPA-5GT*). The infiltration spots are labelled according to the DODA variant. The vector, pBC-BvDODAα1, was included as a positive control (P). pLUC was used as a negative control. **(b)** Betanin quantification in each of the leaves infiltrated with the *M. crystallinum DODA* genes and the pBC-BvDODAα1 positive control. "CB2" corresponds to the leaf shown in **(a)**. Unlike Figures S7, S8 and S10, for which the data were sampled at 4 days post-infiltration, these samples were taken at 5 days post-infiltration. **(c)** A representative HPLC trace is shown for each spot from the "CB2" replicate. An external betanin standard (Betanin) was used to validate the retention time of betanin in these samples and calculate absolute amounts of betanin. The left peak is betanin and the right peak is its isomer, isobetanin.

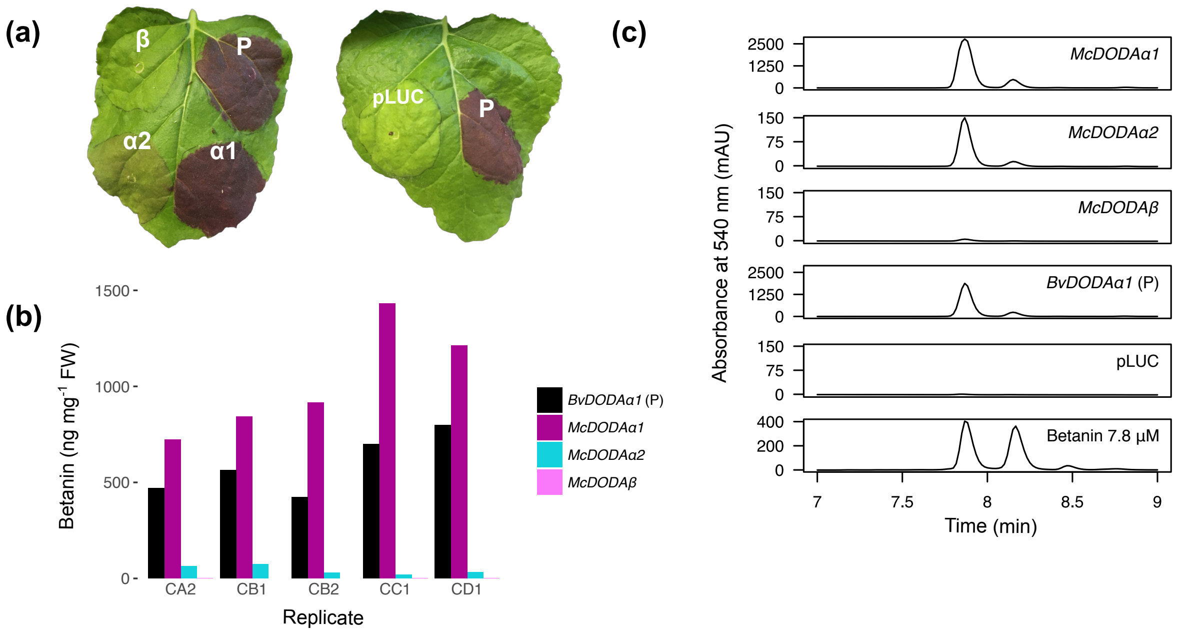

**Figure S11** Functional characterisation of the full complement of *DODA* genes from *Carnegiea gigantea*.

**(a)** Representative *Nicotiana bethamiana* leaves are shown from the agrobacterium infiltration of *DODA* genes from *C. gigantea*. The *DODA* coding sequences were cloned into multi-gene constructs containing the other structural genes necessary for betacyanin production (*BvCYP76AD1*, *MjcDOPA-5GT*). The infiltration spots are labelled according to the DODA variant. The vector, pBC-BvDODAα1, was included as a positive control (P). pLUC was used as a negative control. **(b)** Betanin quantification in each of the leaves infiltrated with the *C. gigantea DODA* genes and the pBC-BvDODAα1 positive control. "Cg2B1" corresponds to the leaf shown in **(a)**. **(c)** A representative HPLC trace is shown for each spot from the "Cg2B1" replicate. An external betanin standard (Betanin) was used to validate the retention time of betanin in these samples and calculate absolute amounts of betanin. The left peak is betanin and the right peak is its isomer, isobetanin.

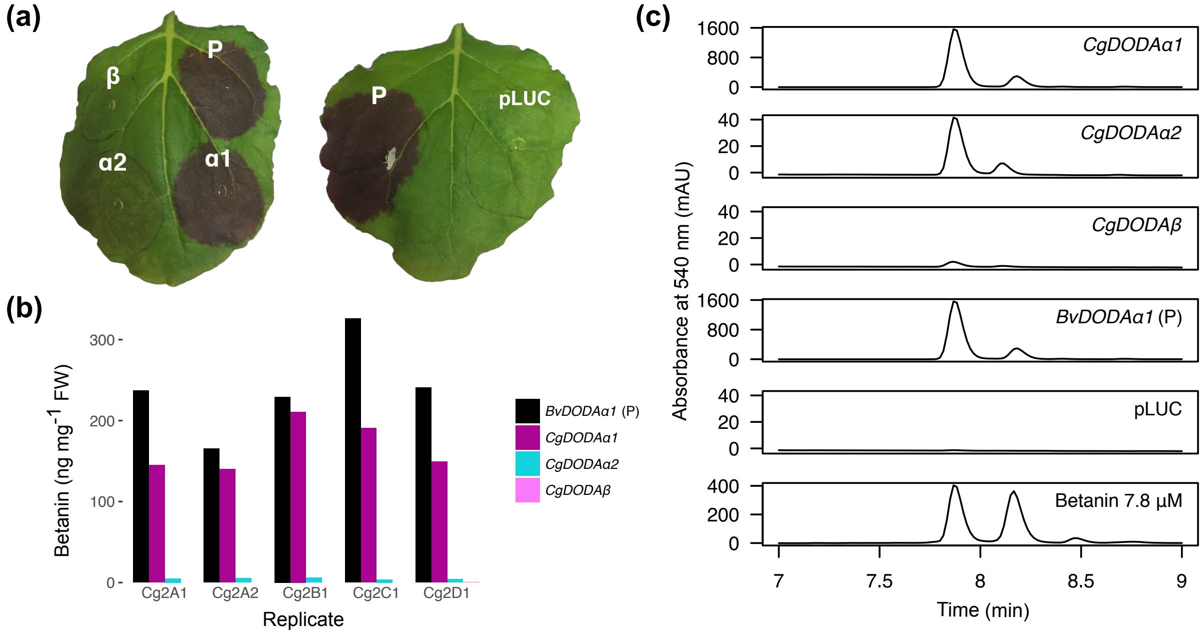

**Figure S12** Functional characterisation of *DODAα* genes from anthocyanic Caryophyllales taxa. **(a)** *DODAα* genes from anthocyanic Caryophyllales taxa were cloned into multi-gene constructs containing the other structural genes necessary for betacyanin production (*BvCYP76AD1*, *MjcDOPA-5GT*) and agroinfiltrated into *Nicotiana benthamiana* leaves. Betanin quantification of agrobacterium-infiltrated *N. benthamiana* leaves was carried out using HPLC according to an external betanin standard. Each graph shows the quantification of betanin from each taxa's *DODAα* gene/s compared to the positive control, *BvDODAα1*. nd, no data. **(b)** A representative HPLC trace is shown for each infiltration spot. An external betanin standard (Betanin) was used to validate the retention time of betanin in these samples. The left peak is betanin and the right peak is its isomer, isobetanin.

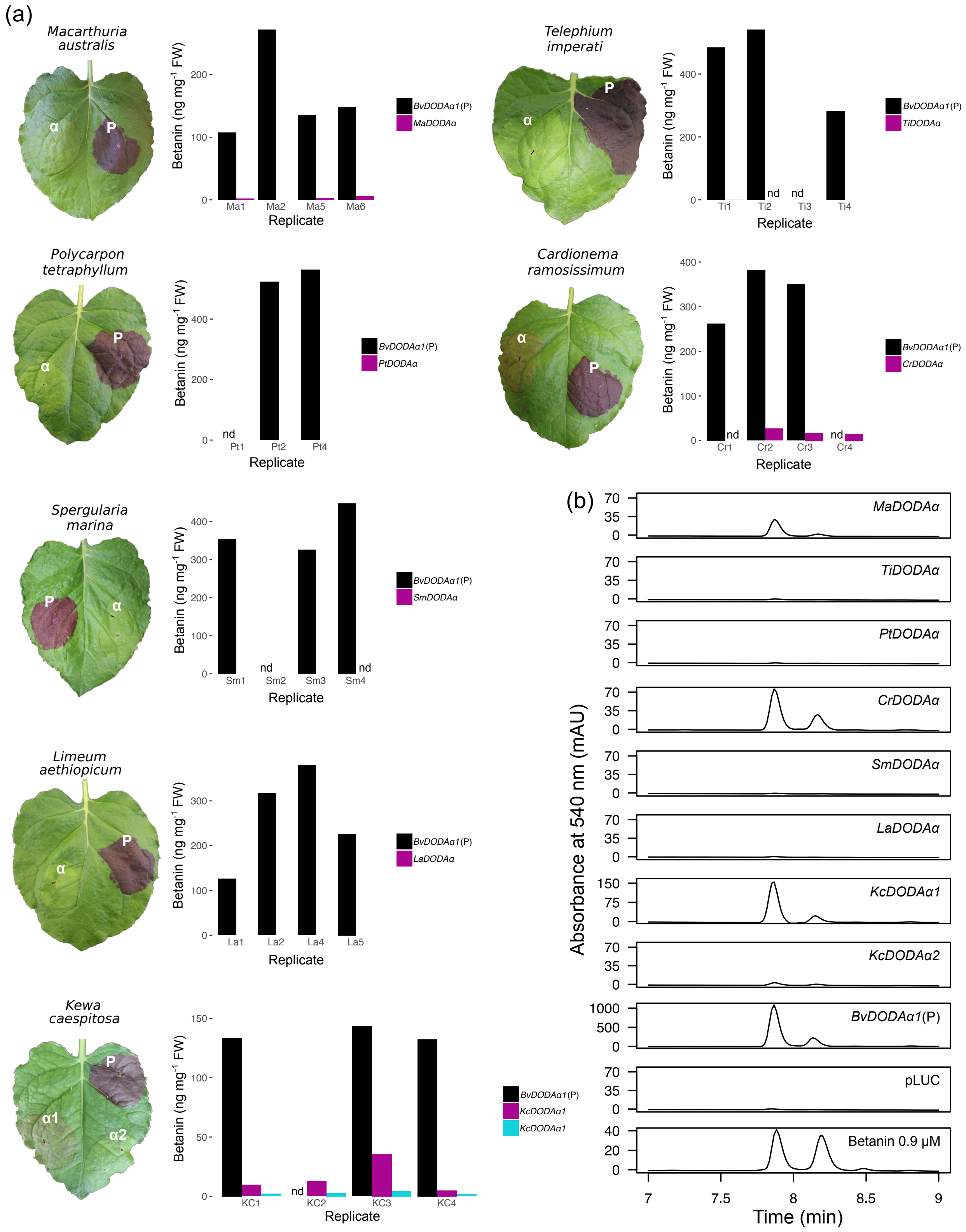
(a)
Macarthuria australis
Betanin (ng mg-1 FW)
Ma1
Ma2
Ma5
Ma6
Replicate
BvDODAα1(P)
MaDODAα
Telephium imperati
nd
Ti1
Ti2
Ti3
Ti4
TiDODAα
Polycarpon tetraphyllum
Pt1
Pt2
Pt4
PtDODAα
Cardionema ramosissimum
Cr1
Cr2
Cr3
Cr4
CrDODAα
Spergularia marina
Sm1
Sm2
Sm3
Sm4
SmDODAα
Limeum aethiopicum
La1
La2
La4
La5
LaDODAα
Kewa caespitosa
KC1
KC2
KC3
KC4
KcDODAα1
KcDODAα1
(b)
Absorbance at 540 nm (mAU)
MaDODAα
TiDODAα
PtDODAα
CrDODAα
SmDODAα
LaDODAα
KcDODAα1
KcDODAα2
BvDODAα1(P)
pLUC
Betanin 0.9 μM
Time (min)

**Figure S13** Taxon-labeled subsampled maximum likelihood *DODAα* gene tree with nucleotide branch lengths and support values. Sequences were subsampled from the *DODAα* gene tree using a custom script, and a tree with support values was inferred with RAxML. Branch labels are support from 200 rapid bootstrap replicates and the SH-aLRT, respectively (RBS/SH-aLRT). Branch lengths are expected substitutions per site (**pdf**).

**Figure S14** Taxon-labeled subsampled maximum likelihood *DODAα* gene tree with codon branch lengths. The subsampled *DODAα* gene tree (Fig. **S13**) was used to optimize codon branch lengths under the best-fitting model of codon substitution (GY+F1X4+Γ(4)) and infer ancestral sequences using IQ-TREE. Branch lengths are expected substitutions per codon (**pdf**).

**Figure S15** Taxon-labeled subsampled maximum likelihood *DODAα* gene tree with amino acid branch lengths**.** The subsampled *DODAα* gene tree (Fig. **S13**) was used to optimize amino acid branch lengths under the best-fitting model of amino acid substitution (JTT+Γ(4)) and infer ancestral sequences using IQ-TREE. Branch lengths are expected substitutions per site (**pdf**).

**Figure S16** Ancestral amino acid sequence reconstruction for seven residues on subsampled *DODAα* gene tree. Ancestral sequences were inferred from the amino acid alignment of a subsample of *DODAα* sequences under the best-fitting model of amino acid substitution (JTT+Γ(4)) with IQ-TREE. Logo plots at nodes are the posterior probabilities of different states for the seven residues identified by Bean *et al.* (2018). Numbers mark the three putative origins of high levels of DODA activity.

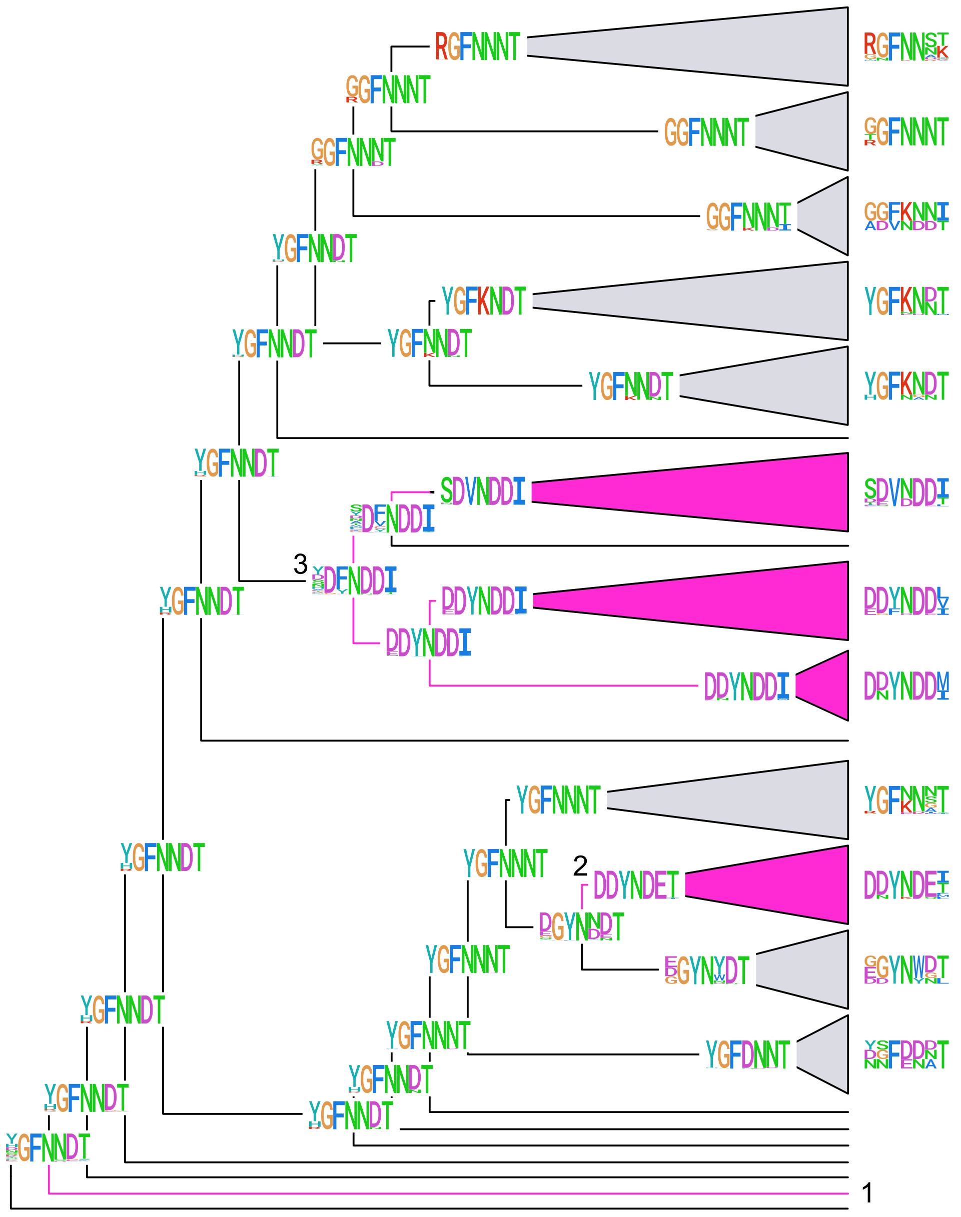

**Table S1** Oligonucleotide primers used in this study

| Name | Sequence (5' to 3') | Gene | Purpose |
|---|---|---|---|
| Bv_DODA_A_variant1F | ATGAAAATGATGAATGGAGAAG | *BvDODAα1* | Gene isolation |
| Bv_DODA_A_variant1R | CTAGGCTGAAGTGAACTTGTAG | *BvDODAα1* | Gene isolation |
| BvDODAα1_lvl0_F | TGAAGACATAATGAAAATGATGAATGGAGAAG | *BvDODAα1* | Goldengate cloning |
| BvDODAα1_lvl0_inR | TGAAGACATTGTATCTTCTACTGTTAATATTGG | *BvDODAα1* | Goldengate cloning |
| BvDODAα1_lvl0_inF | TGAAGACATTACACATCCATTAAGACCTTTC | *BvDODAα1* | Goldengate cloning |
| BvDODAα1_lvl0_R | TGAAGACATAAGCCTAGGCTGAAGTGAACTTGTAGGA | *BvDODAα1* | Goldengate cloning |
| Bv_DODA_A_variant2F | ATGGGTAGTGAAGATAACATCA | *BvDODAα2* | Gene isolation |
| Bv_DODA_A_variant2R | TTAGGTGGAAGTGAACTTGTAG | *BvDODAα2* | Gene isolation |
| BvDODAα2_lvl0_F | TTGAAGACATAATGGGTAGTGAAGATAACATC | *BvDODAα2* | Goldengate cloning |
| BvDODAα2_lvl0_R | TTGAAGACAAAAGCTTAGGTGGAAGTGAACTTGTAG | *BvDODAα2* | Goldengate cloning |
| Bv_DODA_A_variant3F | ATGGCACTTGATGATACAAAG | *BvDODAα3* | Gene isolation |
| Bv_DODA_A_variant3R | TTAGGTGGAAGTGAACTTATAGG | *BvDODAα3* | Gene isolation |
| BvDODAα3_lvl0_F | TTGAAGACATAATGGCACTTGATGATACAAAG | *BvDODAα3* | Goldengate cloning |
| BvDODAα3_lvl0_R | TTGAAGACAAAAGCTTAGGTGGAAGTGAACTTATAG | *BvDODAα3* | Goldengate cloning |
| Bv_DODA_B_variant2F | ATGGGTGGAGAGAAAATGAT | *BvDODAα4/ YTiBvDODAα4* | Gene isolation |
| Bv_DODA_B_variant2R | TCAGGTGGAAGTGAACTTGTA | *BvDODAα4/ YTiBvDODAα4* | Gene isolation |
| YTiBvDODAα4_lvl0_F | TTGAAGACATAATGGATGGAGAGAAAATGATC | *BvDODAα4/ YTiBvDODAα4* | Goldengate cloning |
| YTiBvDODAα4_lvl0_inR | TGAAGACATTGTGACCCAGGAGCCGGATAC | *BvDODAα4/ YTiBvDODAα4* | Goldengate cloning |
| YTiBvDODAα4_lvl0_inF | TGAAGACATTCACCAGAATTGGCAAAAAAG | *BvDODAα4/ YTiBvDODAα4* | Goldengate cloning |
| YTiBvDODAα4_lvl0_R | TTGAAGACAAAAGCTTAGGTGGAAGTGAACTTG | *BvDODAα4/ YTiBvDODAα4* | Goldengate cloning |
| HS3 | TGGAACTCGTACTATGGCACT | *BvDODAα5* | Gene isolation |
| Bv_DODA_A_variant3R | TTAGGTGGAAGTGAACTTATAGG | *BvDODAα5* | Gene isolation |
| BvDODAα5_lvl0_F | TTGAAGACATAATGGCACTTGATGATTCAAAG | *BvDODAα5* | Goldengate cloning |
| BvDODAα5_lvl0_R | TTGAAGACAAAAGCTTAGGTGGAAGTGAACTTATAG | *BvDODAα5* | Goldengate cloning |
| Bv_DODA_B_variant1F | ATGAGTTATAATAGTGGTGCAAAAATTATG | *BvDODAβ* | Gene isolation |
| Bv_DODA_B_variant1R | TTAGTTCGGGGAGGTGAACT | *BvDODAβ* | Gene isolation |
| BvDODAβ_lvl0_F | TGAAGACATAATGAGTTATAATAGTGGTGCA | *BvDODAβ* | Goldengate cloning |
| BvDODAβ_lvl0_R | TGAAGACATAAGCTTAGTTCGGGGAGGTGAACTTGTAG | *BvDODAβ* | Goldengate cloning |
| MaDODAα_F1 | ATGGATGGAACAGAGGAAAC | *MaDODAα* | Gene isolation |
| MaDODAα_R1 | TCAAGTGGAAGTGAACTTGTAG | *MaDODAα* | Gene isolation |
| MaDODAα_lvl0_F | TGAAGACATAATGGATGGAACAGAGGAAAC | *MaDODAα* | Goldengate cloning |
| MaDODAα_lvl0_inF | TGAAGACATGGTTCTCACGATTTAGCGA | *MaDODAα* | Goldengate cloning |
| MaDODAα_lvl0_inR | TGAAGACATAACCAGGTGCCGGATACT | *MaDODAα* | Goldengate cloning |
| MaDODAα_lvl0_R | TGAAGACATAAGCTCAAGTGGAAGTGAACTTGT | *MaDODAα* | Goldengate cloning |
| LaDODAα_F1 | ATGACTAGCGAGACGTTCTAC | *LaDODAα* | Gene isolation |
| LaDODAα_R1 | TCAGATGGAAGTGAACTTGTAG | *LaDODAα* | Gene isolation |
| LaDODAα_lvl0_F | TGAAGACATAATGACTAGCGAGACGTTCTA | *LaDODAα* | Goldengate cloning |
| LaDODAα_lvl0_inF | TGAAGACGAGGACGTTAATCATTACA | *LaDODAα* | Goldengate cloning |
| LaDODAα_lvl0_inR | TGAAGACACGTCCTCATACCTTCCACTGA | *LaDODAα* | Goldengate cloning |
| LaDODAα_lvl0_R | TGAAGACATAAGCTCAGATGGAAGTGAAC | *LaDODAα* | Goldengate cloning |
| KcDODAα1_F1 | GAAATCAAAAGGACACCGACA | *KcDODAα1* | Gene isolation |
| KcDODAα1_R1 | CGCAGACAATAACAAAAAGTAACAC | *KcDODAα1* | Gene isolation |
| KcDODAα1_F2 | GGACACCGACAGTCAGTAGCA | *KcDODAα1* | Gene isolation |
| KcDODAα1_R2 | AACTAAACCACACGCAGACAA | *KcDODAα1* | Gene isolation |
| KcDODAα1_lvl0_F | TGAAGACATAATGGGTGCGGAGGACATGCT | *KcDODAα1* | Goldengate cloning |
| KcDODAα1_lvl0_inF1 | TGAAGACATCCAGCTCAAGTATCCGGTTTCTGG | *KcDODAα1* | Goldengate cloning |
| KcDODAα1_lvl0_inF2 | TGAAGACATGAAGGTATGAAGATGTGATTCATT | *KcDODAα1* | Goldengate cloning |
| KcDODAα1_lvl0_inR1 | TGAAGACATCTGGTACATGGAAGCAGGGC | *KcDODAα1* | Goldengate cloning |
| KcDODAα1_lvl0_inR2 | TGAAGACATCTTCCGCTGATCAGAGTTTC | *KcDODAα1* | Goldengate cloning |
| KcDODAα1_lvl0_R | TGAAGACATAAGCCTAGGTGGAGGTGAACTTAT | *KcDODAα1* | Goldengate cloning |
| KcDODAα2_F1 | GCCAAACCCGTACCAAACCT | *KcDODAα2* | Gene isolation |
| KcDODAα2_R1 | CCCCACTAAACAAGCAAAATG | *KcDODAα2* | Gene isolation |
| KcDODAα2_F2 | ATGAATTGTGATTTGTGCCTTCCT | *KcDODAα2* | Gene isolation |
| KcDODAα2_R2 | GCTTTAAGTGGAAGTGAACTTGTAGG | *KcDODAα2* | Gene isolation |
| KcDODAα2_lvl0_F | TGAAGACATAATGGGTGGTGGTGATGTGAT | *KcDODAα2* | Goldengate cloning |
| KcDODAα2_lvl0_inF1 | TGAAGACATCCAGTTCAAGTACCCAGCTCCATG | *KcDODAα2* | Goldengate cloning |
| KcDODAα2_lvl0_inF2 | TGAAGACATTCTTGACCACGCCTCATGGG | *KcDODAα2* | Goldengate cloning |
| KcDODAα2_lvl0_inF3 | TGAAGACATGAAGGTATGAAGATGTGAACAATA | *KcDODAα2* | Goldengate cloning |
| KcDODAα2_lvl0_inR1 | TGAAGACATCTGGTACATTTGAGCAGGGA | *KcDODAα2* | Goldengate cloning |
| KcDODAα2_lvl0_inR2 | TGAAGACATAAGACCACGCTTCTTGTCTA | *KcDODAα2* | Goldengate cloning |
| KcDODAα2_lvl0_inR3 | TGAAGACATCTTCCACTAGTTAGAGCTTC | *KcDODAα2* | Goldengate cloning |
| KcDODAα2_lvl0_R | TGAAGACATAAGCTTAAGTGGAAGTGAACTTGT | *KcDODAα2* | Goldengate cloning |

| **Name** | **Sequence (5' to 3')** | **Gene** | **Purpose** |
|---|---|---|---|
| CarDODAα F14 | GAGACRTTCTTCATATCACAYGG | Caryophyllaceae *DODAα* | Gene isolation |
| CarDODAα R14 | TCARCMGGAAGYGAACTTGTA | | |
| CarDODAα F15 | ATYCTYRTSRTCTCYGCTCA | | |
| CarDODAα R15 | TGYAAYGGRTAGAAGTGCTC | | |
| CarDODAα_Fx | AWCAIYAGITISAAAATGGGC | | |
| CarDODAα_F16 | ATCCCKGTTTGTCAGCTCTCT | | |
| CarDODAα_R16 | AGAGAGCTGACAAACMGGGAT | | |
| CarDODAα_F17 | GAAGGTGTYCTCATMATYGGT | | |
| CarDODAα_R17 | ACCRATKATGAGRACACCTTC | | |
| CarDODAα_R18 | GAAGGGTGIGTGGYACTTCC | | |
| CarDODAα_R19 | CAKTSATCAAACTCIGCAGCCCA | | |
| CarDODAα_R20 | GTTTCCCARTGAGCRGAGA | | |
| Cr3GPS1 | AGGTGCAGGGTGAGGATGGTCGAGATG | *CrDODAα* | Gene isolation |
| Cr3GPS2 | ACCCATATGTTGGGCCGTGTCCTTGA | | |
| CrDODAα_F1 | ATGACCTCTGAAGAAGCGAAAGAGATCA | | |
| CrDODAα_R1 | TCAGGTGGAAGTGAACTTATAGGAGCCA | | |
| CrDODAα_lvl0_F | TGAAGACATAATGACCTCTGAAGAAGCGAA | | Goldengate cloning |
| CrDODAα_lvl0_inF | TGAAGACATGATCTCCACAATTGGCAA | | |
| CrDODAα_lvl0_inR | TGAAGACATGATCCAGGAGCTGGATACT | | |
| CrDODAα_lvl0_R | TGAAGACATAAGCTCAGGTGGAAGTGAACTTAT | | |
| PtDODAα_F1 | ATGCATTGCTTAACTTTTCAACC | *PtDODAα* | Gene isolation |
| PtDODAα_F2 | ATGATGAGAAGATGAACGAAAC | | |
| PtDODAα_R1 | TCAGGTGGAAGTGAATTTATAAG | | |
| PtDODAα_F3 | ATGATGATGCATTGCTTAACTTTTCAACC | | |
| PtDODAα_R3 | TCAGGTGGAAGTGAATTTATAAGAGCCA | | |
| PtDODAα_lvl0_F | TGAAGACATAATGCATTGCTTAACTTTTCA | | Goldengate cloning |
| PtDODAα_lvl0_R | TGAAGACATAAGCTCAGGTGGAAGTGAATT | | |
| TiDODAα_F1 | CCAACTACCAACATAAGCACT | *TiDODAα* | Gene isolation |
| TiDODAα_R1 | TGGACAACAATGCAAAGGAG | | |
| TiDODAα_F2 | ACTGACTGTCTTGATCTGCCA | | |
| TiDODAα_R2 | TTGTTCTTGGCGATGTCAGTA | | |
| TiDODAα_F3 | CCACACTGGAAAAAGGCACAT | | |
| TiDODAα_R3 | GGAAAGCTGTCGAAATCGTAGA | | |
| TiDODAα_R4 | ATCAGTTTCCCAGTGAGC | | |
| TiDODAα_F5 | CACCAAAAACAGGTTTATCTAACG | | |
| TiDODAα_R5 | AAGAGGTCTAATTGCTCACGAC | | |
| TiDODAα_F6 | ATGAGTGACGAGCAAATGATC | | |
| TiDODAα_R6 | GCTGGAAGTGAATTTGTAGGAGG | | |
| TiDODAα_lvl0_F | TGAAGACATAATGAGTGACGAGCAAATGAT | | Goldengate cloning |
| TiDODAα_lvl0_inF1 | TGAAGACATTCAGTTTAAGTACCCAGCTCCTGG | | |
| TiDODAα_lvl0_inR1 | TGAAGACATCTGATACATATAAGCAGGAA | | |
| TiDODAα_lvl0_inF2 | TGAAGACATGAAGGTATGAAGTAGTGAATAATTG | | |
| TiDODAα_lvl0_inR2 | TGAAGACATCTTCCTGTCGTCAGAGCTT | | |
| TiDODAα_lvl0_inR | TGAAGACATAAGCTCAGCTGGAAGTGAATTTGT | | |
| SmDODAα_F11 | GCTACAAAATGGGCTGTCAAGAG | *SmDODAα* | Gene isolation |
| SmDODAα_F11' | ATGGGCTGTCAAGAGATGACT | | |
| SmDODAα_R11 | AGAGGTGGCATTGGAAGGGT | | |
| SmDODAα_F12 | TGGGCTGTCAAGAGATGACT | | |
| SmDODAα_F12-2 | ATGGGCTGTCAAGAGATGACT | | |
| SmDODAα_R12 | TCAACCGGAAGTGAACTTGT | | |
| SmDODAα_lvl0_F | TGAAGACATAATGGGCTGTCAAGAGATGAC | | Goldengate cloning |
| SmDODAα_lvl0_inF | TGAAGACGAGGACGTGAATAACTATG | | |
| SmDODAα_lvl0_inR | TGAAGACACGTCCTCGTACCTTCCATTTG | | |
| SmDODAα_lvl_R | TGAAGACATAAGCTCAACCGGAAGTGAACTTG | | |

**Table S2** GenBank accession numbers for genes functionally characterised in this study

| Species | ***DODA*** **gene** | Genbank ID | Alternative name |
|---|---|---|---|
| *Beta vulgaris* | *BvDODAα1* | MN153194 | BvDODA1 (Hatlestad *et al.*, 2012; HQ656027.1) |
| *Beta vulgaris* | *BvDODAα2* | MN153195 | BvDODA2 (Hatlestad *et al.*, 2012; HQ656022.1); BvDODA (Christinet *et al.*, 2004; AJ583017) |
| *Beta vulgaris* | *BvDODAα3* | MN153196 | *BvDODA2c (*Bean *et al., 2018)* |
| *Beta vulgaris* (YTiBv) | *BvDODAα4* | MN136228 | BvDODA2b (Bean *et al.*, 2018) |
| *Beta vulgaris* | *BvDODAα5* | MN153197 | BvDODA2d (Bean *et al.*, 2018) |
| *Beta vulgaris* | *BvDODAβ* | MN153198 | - |
| *Stegnosperma halimifolium* | *ShDODAα1* | MN136219 | - |
| *Stegnosperma halimifolium* | *ShDODAα2* | MN136220 | - |
| *Stegnosperma halimifolium* | *ShDODAβ* | MN136221 | - |
| *Mesembryanthemum crystallinum* | *McDODAα1* | MN136225 | - |
| *Mesembryanthemum crystallinum* | *McDODAα2* | MN136226 | - |
| *Mesembryanthemum crystallinum* | *McDODAβ* | MN136227 | - |
| *Carnegiea gigantea* | *CgDODAα1* | MN136222 | - |
| *Carnegiea gigantea* | *CgDODAα2* | MN136223 | - |
| *Carnegiea gigantea* | *CgDODAβ* | MN136224 | - |
| *Spergularia marina* | *SmDODAα* | MN153204 | - |
| *Cardionema ramosissimum* | *CrDODAα* | MN153200 | - |
| *Telephium imperati* | *TiDODAα* | MN153212 | - |
| *Polycarpon tetraphyllum* | *PtDODAα* | MN153211 | - |
| *Macarthuria australis* | *MaDODAα* | MN153205 | - |
| *Kewa caespitosa* | *KcDODAα1* | MN153209 | - |
| *Kewa caespitosa* | *KcDODAα2* | MN153210 | - |
| *Limeum aethiopicum* | *LaDODAα* | MN153203 | - |
| *Pollichia campestris* | *PcDODAα* | MN153206 | - |
| *Corrigiola litoralis* | *ClDODAα* | MN153199 | - |

**Table S3** Pigmentation status of 174 genera of Caryophyllales gathered from the literature. Pigments (anthocyanins: 0, betalains: 1) and literature reference (**xls**).

**Table S4** Genome and transcriptome assemblies used in this study. Genome and transcriptome assemblies used both for individual *DODA* searches and automated baited homology searches. Species: family, genus and species, or genus and species for outgroup taxa; Code: the code used to identify this assembly in this study; Genome (transcriptome: 0, genome:1); Version: the assembly and/or annotation version; Source: the data depository or online database used to access the assembly; Reference: the literature reference for this assembly or underlying data; Notes: miscellaneous notes regarding this assembly; Reads accession: GenBank accession for raw reads; Assembly accession: accession number for GenBank-sourced assemblies; URL: a URL for quick access to assembly details (**xls**).

**Table S5** *DODA* sequences used in *DODA* phylogeny. The *DODA* sequences included in the final gene tree are presented here (homologous sequence dataset, trimmed of sequences containing 50% or more gaps in the cleaned alignment). Taxon: the family, genus and species, or genus and species, of the taxa from which the sequence originated. Asterisks demarcate outgroup species; Sequence Identifier: the unique code used to identify this sequence in this study; Accession: accession for single sequences that are available on GenBank; Bait: was the sequence included in the initial bait set used to search genome and transcriptome assemblies (no: 0, yes: 1); in_ASR: was the sequence included in the subset of sequences used to infer ancestral *DODAα* sequences (no: 0, yes: 1) (**xls**).

**Table S6** Details of *DODA* genes that have been functionally characterised from Caryophyllales species and mapped to the *DODA* gene tree

| Species | Name | Accession | Publication | Method/Assay of functional characterisation | Corresponding phenotype |
|---|---|---|---|---|---|
| *Stegnosperma halimifolium* | *ShDODAα1* | MN136219 | This study | Agroinfiltration in *Nicotiana benthamiana* | High levels of betanin |
| *Stegnosperma halimifolium* | *ShDODAα2* | MN136220 | This study | Agroinfiltration in *N. benthamiana* | Marginal levels of betanin |
| *Stegnosperma halimifolium* | *ShDODAβ* | MN136221 | This study | Agroinfiltration in *N. benthamiana* | Marginal levels of betanin |
| *Beta vulgaris* | *BvDODAα1* | MN153194 | This study | Agroinfiltration in *N. benthamiana* | High levels of betanin |
| *Beta vulgaris* | *BvDODAα2* | MN153195 | This study | Agroinfiltration in *N. benthamiana* | Weakly pigmented |
| *Beta vulgaris* | *BvDODAα3* | MN153196 | This study | Agroinfiltration in *N. benthamiana* | No betanin present |
| *Beta vulgaris* | *BvDODAα4* | MN136228 | This study | Agroinfiltration in *N. benthamiana* | Marginal levels of betanin |
| *Beta vulgaris* | *BvDODAα5* | MN153197 | This study | Agroinfiltration in *N. benthamiana* | No betanin present |
| *Beta vulgaris* | *BvDODAβ* | MN153198 | This study | Agroinfiltration in *N. benthamiana* | Marginal levels of betanin |
| *Mesembryanthemum crystallinum* | *McDODAα1* | MN136225 | This study | Agroinfiltration in *N. benthamiana* | High levels of betanin |
| *Mesembryanthemum crystallinum* | *McDODAα2* | MN136226 | This study | Agroinfiltration in *N. benthamiana* | Marginal levels of betanin |
| *Mesembryanthemum crystallinum* | *McDODAβ* | MN136227 | This study | Agroinfiltration in *N. benthamiana* | Marginal levels of betanin |
| *Carnegiea gigantea* | *CgDODAα1* | MN136222 | This study | Agroinfiltration in *N. benthamiana* | High levels of betanin |
| *Carnegiea gigantea* | *CgDODAα2* | MN136223 | This study | Agroinfiltration in *N. benthamiana* | Marginal levels of betanin |
| *Carnegiea gigantea* | *CgDODAβ* | MN136224 | This study | Agroinfiltration in *N. benthamiana* | Marginal levels of betanin |
| *Macarthuria australis* | *MaDODAα* | MN153205 | This study | Agroinfiltration in *N. benthamiana* | Marginal levels of betanin |
| *Limeum aethiopicum* | *LaDODA* | MN153203 | This study | Agroinfiltration in *N. benthamiana* | No betanin present |
| *Kewa caespitosa* | *KcDODAα1* | MN153209 | This study | Agroinfiltration in *N. benthamiana* | Marginal levels of betanin |
| *Kewa caespitosa* | *KcDODAα2* | MN153210 | This study | Agroinfiltration in *N. benthamiana* | Marginal levels of betanin |
| *Polycarpon tetraphyllum* | *PtDODA* | MN153211 | This study | Agroinfiltration in *N. benthamiana* | No betanin present |
| *Cardionema ramosissimum* | *CrDODA* | MN153200 | This study | Agroinfiltration in *N. benthamiana* | Marginal levels of betanin |
| *Spergularia marina* | *SmDODAα* | MN153204 | This study | Agroinfiltration in *N. benthamiana* | No betanin present |
| *Telephium imperati* | *TiDODA* | MN153212 | This study | Agroinfiltration in *N. benthamiana* | Marginal levels of betanin |
| *Beta vulgaris* | *BvDODAα1* | HQ656027.1 | Bean *et al.*, 2018 | Overexpression in acyanic *Beta vulgaris* | Yellow pigment |
| *Beta vulgaris* | *BvDODAα1* | HQ656027.1 | Bean *et al.*, 2018 | VIGS in *B. vulgaris* | Loss of pigmentation |
| *Beta vulgaris* | *BvDODAα1* | HQ656027.1 | Bean *et al.*, 2018 | Expression in *Saccharomyces cerevisiae* | DOPA-betaxanthin produced |
| *Beta vulgaris* | *BvDODAα2* | KM502868 | Bean *et al.*, 2018 | Overexpression in acyanic *B. vulgaris* | No yellow pigment |
| *Beta vulgaris* | *BvDODAα2* | KM502868 | Bean *et al.*, 2018 | VIGS in *B. vulgaris* | No loss of pigmentation |
| *Beta vulgaris* | *BvDODAα2* | KM502868 | Bean *et al.*, 2018 | Expression in *S. cerevisiae* | No DOPA-betaxanthin produced |
| *Chenopodium quinoa* | *CgDODA1* | XP_021769303 | Imamura *et al.*, 2017 | Agroinfiltration in *N. benthamiana* | High levels of betanin |

| **Species** | **Name** | **Accession** | **Publication** | **Method/Assay of functional characterisation** | **Corresponding phenotype** |
|---|---|---|---|---|---|
| *Calandrinia mirabilis (Parakeelya mirabilis)* | *PmDOD* | KF747352.1 | Chung *et al.,* 2015 | Biolistic transformation in *Antirrhinum majus* and *Allium cepa* | Strongly pigmented and autofluorescent |
| *Calandrinia mirabilis (Parakeelya mirabilis)* | *PmDOD-like* | KF747353.1 | Chung *et al.,* 2015 | Biolistic transformation in *A. majus* and *A. cepa* | Weakly pipgmented and autofluorescent |
| *Ptilotus hybrid* | *PhybDOD-like* | KF747354.1 | Chung *et al.,* 2015 | Biolistic transformation in *A. majus* and *A. cepa* | Weakly pipgmented and autofluorescent |
| *Beta vulgaris* | *BvDODAα1* | HQ656027.1 | Chung *et al.,* 2015 | Biolistic transformation in *A. majus* | Strongly pigmented and autofluorescent |
| *Beta vulgaris* | *BvDODA* | AJ583017 | Chung *et al.,* 2015 | Biolistic transformation in *A. majus* | Weakly pipgmented and autofluorescent |
| *Dianthus superbus* | *DsLigB* | JL390264 | Chung *et al.,* 2015 | Biolistic transformation in *A. majus* | No pigmentation |
| *Beta vulgaris* | *BvDODAα2* | AJ583017 | Gandía-Herrero & García-Carmona, 2012 | Recombinant expression in *Escherichia coli* | Forms betalamic acid from *L*-DOPA |
| *Beta vulgaris* | *BvDODAα1* | HQ656027.1 | Hatlestad *et al.*, 2012 | VIGS in *B. vulgaris* | Loss of pigmentation |
| *Mirabilis jalapa* | *MjDOD* | AB435372.1 | Sasaki *et al.*, 2009 | Recombinant expression in *E. coli* | Forms betalamic acid from *L*-DOPA |
| *Portulaca grandiflora* | *PgDODA* | AJ580598.1 | Christinet *et al.*, 2004 | Complementation of acyanic mutant | Restores pigmentation |

**Methods S1** Genome assembly

Sequencing of *Corrigiola litoralis*, *Limeum aethiopicum*, *Simmondsia chinensis* and *Spergula arvensis* was performed on a HiSeq X-Ten with one sample per lane. Processing of sequencing data, assembly via SOAPdenovo2 v2.04-r240 (Luo *et al.*, 2012), downstream processing, and gene prediction via AUGUSTUS v3.3 (Stanke *et al.*, 2008) was based on the previously described workflow (Pucker *et al.*, 2019). Assemblies of *C. litoralis* (ERS2294062), *L. aethiopicum* (ERZ869968), *S. chinensis* (ERZ497710), and *S. arvensis* (ERS2294061) are available at the EBI. AUGUSTUS v3.3 (Stanke *et al.*, 2008) was applied as previously described to predict protein-coding genes for these assemblies (Pucker *et al.*, 2019). Additionally, genome assemblies of *Lophocereus schottii* (GCA_002740545.1), *Pachycereus pringlei* (GCA_002740445.1), *Pereskia humboldtii* (GCA_002740485.1), *Stenocereus thurberi* (GCA_002740465.1), *Kewa caespitosa* (GCA_900322205), *Macarthuria australis* (GCA_900322265) and *Pharnaceum exiguum* (GCA_9003222385) were added to the downstream analysis.

**Methods S2** Identification of full complements of *DODA* genes from betalain-pigmented species for functional analysis

*Beta vulgaris*

Using BLAST searches with the protein sequence of the previously characterized *BvDODAα1* (Hatlestad *et al.*, 2012), the *B. vulgaris* RefBeet (v1.2.2) genome contains six full-length and one partial annotated predicted genes that have homology to *DODA* (Brockington *et al.*, 2015; Table **S2**). For this publication, the full-length *DODA* genes were isolated from *B. vulgaris* 'Bolivar' with one exception. *BvDODAα4* was amplified from cDNA from leaf tissue of *B. vulgaris* sugarbeet 'YTiBv' using the YTiBv-1.0 genome assembly (scaffold13748: 34334..33958, 34808..34555, 32278..32057; Table **S4**).

The partial *DODA* sequence (XM_010676273.2) was also investigated. The first exon of *BvDODAα6* has no protein homology to other *DODAs* and the second exon is half the expected size. BLAST searches were carried out upstream of the annotated regions, up to the next annotated gene, in order to search for regions of homology to *DODA* sequences. Some extra sequences were found but they were either very short, or in a different reading frame. Because this transcript does not encode a full-length DODA protein, they likely represent incomplete tandem duplications or pseudogenisations and thus were not investigated further for DODA-specific function.

*Carnegiea gigantea*

Four *DODA* genes were identified by performing a BLAST search using representative *DODA* genes from *B. vulgaris* in the *C. gigantea* genome assembly SGPv1.3 and its corresponding predicted genes (Table **S4**; PRJNA318822; Copetti *et al.*, 2017). The predicted transcript, Cgig1_02616, was found to be composed of coding sequences corresponding to two *DODA* genes, referred to as *CgDODAα2* and *CgDODAβ*. These were manually annotated by comparison with other *DODA* genes, and by comparison with RNAseq datasets (Copetti *et al.*, 2017). Cgig1_02617 is directly upstream from the Cgig1_02616 annotation. The predicted exons only have partial homology, and contain in-frame stop codons. By BLASTing the sequence between this transcript and Cgig1_02616, an extra region with homology to exon 3 of the *DODA* genes could be found, but this region contains two stop

codons and a frame-shift. There is also no RNAseq evidence for this gene, altogether indicating that this is likely a pseudogenisation arising after tandem duplication of *CgDODAα2*, and thus this gene was not investigated further.

*Mesembryanthemum crystallinum*

Amino acid sequences of the *B. vulgaris DODAs* were retrieved from the RefBeet v1.2 genome database (Dohm *et al.*, 2014). The amino acid sequences were mapped against the *M. crystallinum* genome annotation (W. C. Yim, unpublished) using TBLASTN from BLAST (Camacho *et al.*, 2009). The identified CDS sequences were manually curated and confirmed by RNAseq reads support.

**Methods S3** Phylogeny of Caryophyllales

To enable trait reconstruction across the order, we generated a comprehensive genus-level species tree for Caryophyllales using publicly-available sequence data compiled by PyPHLAWD (Smith & Walker, 2019). Briefly, we ran PyPHLAWD with default configurations for the whole of Caryophyllales on the GenBank 'pln' division (downloaded 26/11/2018), used find_good_clusters.py to select the most well-sampled markers, and sampled a single species per genus with the greatest sequence-wise coverage across these markers, ensuring that no infraspecific taxa were selected. Then we reran PyPHLAWD on the same database, restricting the list of retrieved sequences to the selected taxa. We carried forward the seven most well-sampled markers for phylogenetic reconstruction. These were the nucleotide marker ITS and the plastid markers *rbcL*, *matK*, *rps16*, *trnL-trnF*, *rpl16* and *atpB-rbcL*. Alignments were concatenated into a supermatrix and a maximum likelihood phylogeny was inferred using RAxML v8.2.12 (Stamatakis, 2014). We partitioned the alignment by gene and used the overall algorithm to conduct 100 Rapid Bootstrap replicates and thereafter a thorough maximum likelihood tree search from 10 bootstrap starting trees under the GTR+Γ model of sequence evolution. In order to reflect current understanding of the Caryophyllales phylogeny, we incorporated a relatively strong constraint following the MQSST topology inferred from phylogenomic data by Walker *et al.* (2018) and a placement of *Lophiocarpus* and *Corbichonia* following Thulin *et al.* (2016, 2018). However, we did not enforce the non-monophyly of the non-core recovered in the former as it was weakly supported. We rooted the phylogeny with the clade of non-core Caryophyllales. To scale the branches of our species tree to time, we used treePL (Smith & O'Meara, 2012), which uses a penalized likelihood framework (Sanderson, 2002) and is capable of handling large trees. To constrain our inference, we used the same fossil and secondary constraints as Smith *et al.* (2018), and sources therein, except that the root age was that from the UCLN clock analysis of Magallón *et al.* (2015; max 107.13 mya, min 102.5 mya). We conducted a priming analysis to determine the best starting values and used cross-validation to determine the optimal smoothing parameter.

**Methods S4** Pigment reconstruction

Pigment data at genus level resolution was used to reconstruct the evolution of betalain pigmentation on the time-calibrated genus-level phylogeny of Caryophyllales. We surveyed the literature for pigment data and determined the pigmentation status of 174 genera, classifying them as anthocyanin-pigmented, betalain-pigmented, or unknown (Table **S3**). We further assumed that all non-core Caryophyllales are anthocyanin pigmented. Where only one

species in a genus was tested, we assumed that its pigmentation status was generalizable. We reconstructed pigmentation as a binary trait using maximum likelihood (Pagel, 1994, 1999) and stochastic mapping (Nielsen, 2002; Huelsenbeck *et al.*, 2003) with the ape and phytools R packages, respectively (Revell, 2012; R Core Team, 2019; Paradis & Schliep, 2019). For both methods, we implemented two different forms of the Mk model (Pagel, 1994; Lewis, 2001): i) Symmetric, allowing equal rates (ER) of evolution from anthocyanin to betalain and *vice versa* and ii) Asymmetric (or All Rates Different; ARD), allowing a different rate of evolution from anthocyanin to betalain than from betalain to anthocyanin. Unknown taxa were given an equal likelihood of being in either state. In the maximum likelihood case, we compared model performance with AIC scores (Burnham & Anderson, 2002). For stochastic mapping, we enforced a strong prior that the root of the tree was anthocyanin-pigmented, although prior sensitivity analyses indicated that this was not the cause of our results.

**Methods S5** Gene tree of Caryophyllales *DODA* genes

We compiled a dataset of publicly available and early release genome and transcriptome assemblies. We collected 39 ingroup and outgroup genomes from public repositories and added the full complement of 297 ingroup transcriptomes from Walker *et al.* (2018). We supplemented these with 9 genomes newly assembled or reannotated for this study and 11 Aizoaceae transcriptomes from L. Zhao (unpublished). Full dataset details can be found in Table **S4**. To infer the gene tree of DODA in Caryophyllales, we employed the baited homologue approach following Lopez-Nieves *et al.* (2018). Scripts for this analysis were downloaded from https://bitbucket.org/yangya/adh_2016 and received minor modifications; all scripts can be found at https://github.com/NatJWalker-Hale/DODA. We used a bait set of previously analyzed *DODA* genes from the literature (Table **S5**). In order to include baits from Caryophyllaceae that were putative pseudogenes, we aligned the baits with MACSE v2.03 (Ranwez *et al.*, 2018) with defaults and replaced any codons containing inferred deletions with ambiguities (-), such that the inframe proteins had ambiguities at this site. First, we clustered similar amino acid sequences in transcriptomes using CD-HIT v4.7 (-n 5 -c 0.995) (Fu *et al.*, 2012). We conducted similarity searches using SWIPE v2.1.0 (Rognes, 2011), retaining the top 10 candidates.  We then conducted three rounds of automated iterative alignment inference and tree inference with MAFFT v7.409 (Katoh & Standley, 2013) and FastTree v2.1.10 (Price *et al.*, 2010), monophyletic masking and trimming, before a round of manual curation to produce the homologue set. Manual curation involved adding three baits which were inferred on long branches and erroneously removed during the pipeline, removing redundant sequences where another sequence from the same loci was longer, masking monophyletic transcriptome sequences to remove isoforms, removing sequences on long branches and removing sequences that had radically divergent phylogenetic placement indicative of error. Amino acid sequences of the final homologue set were aligned with MAFFT v7.409 (Katoh & Standley, 2013) (--genafpair --maxiterate 1000), and coding sequences were aligned to the amino acids with the function pxaa2cdn from the phyx package (Brown *et al.*, 2017). The dataset was cleaned of columns with 90% or more missing data using pxclsq from phyx (-p 0.1). Sequences containing over 50% gaps in the cleaned alignment were removed. The resulting dataset was realigned, coding sequences were realigned and cleaned (pxclsq -p 0.1), and RAxML v8.2.12 was used to conduct 200 Rapid Bootstrap replicates and thereafter a thorough maximum likelihood tree search from 10 different

bootstrap starting trees, under the GTR+Γ(4) model of sequence evolution. We also used RAxML to NNI-optimize the tree and generate support values from the SH-like Approximate Likelihood Ratio Test (SH-aLRT) (Guindon *et al.*, 2010). If a better tree was found during NNI optimization, we carried this tree forward and remapped bootstrap replicates to this topology. Finally, we extracted a strongly-supported clade of *DODAα* genes following Brockington *et al.* (2015), and realigned, re-inferred a maximum likelihood tree and generated support using the same approach as previously (Fig. **S1**).

**Methods S6** Ancestral Sequence Reconstruction

To create a sequence dataset computationally and numerically tractable for ancestral sequence reconstruction, we used custom python scripts (subsample_by_paralog.py; https://github.com/NatJWalker-Hale/DODA) to subsample the *DODAα* gene tree (Fig. **S1**), using a strategy designed to maintain within-paralogue diversity. To do this, we used the species overlap algorithm implemented in ete3 (Huerta-Cepas *et al.*, 2016) to break the tree into paralogues, before sampling within each paralogue such that every family had at least its longest sequence in the cleaned alignment or longest sequences proportional to its representation in that paralogue within the original dataset. We created a final dataset of 198 sequences, ensuring that a representative of all functionally characterized *DODAα* loci was included. We aligned and inferred an ML tree for this dataset using the same approach as above (Fig**. S13**). Marginal ancestral sequence reconstructions were conducted on this tree for codons and amino acids in IQ-TREE v1.6.10 (Nguyen *et al.*, 2015; Kalyaanamoorthy *et al.*, 2017) under the best-fitting substitution model (-m MFP -msub nuclear -mrate E,G,R -asr). We tested both the cleaned (pxclsq -p 0.1) and uncleaned alignment. However, because IQ-TREE does not conduct ancestral inference of gaps, we carried forward results from the cleaned alignment. Probabilities of ancestral states at each node for the sites identified by Bean *et al.* (2018) were summarised using logo plots created with the R package ggseqlogo (Wagih, 2017; R Core Team, 2019). Codon reconstructions were visualised as their translated amino acids by summing over the probabilities of redundant codons.

**References**

**Bean A, Sunnadeniya R, Akhavan N, Campbell A, Brown M, Lloyd A, Lloyd A**. **2018**. Gain-of-function mutations in beet DODA2 identify key residues for betalain pigment evolution. *New Phytologist* **219**: 287–296.

**Brockington SF, Yang Y, Gandía-Herrero F, Covshoff S, Hibberd JM, Sage RF, Wong GKS, Moore MJ, Smith SA**. **2015**. Lineage-specific gene radiations underlie the evolution of novel betalain pigmentation in Caryophyllales. *New Phytologist* **207**: 1170–1180.

**Brown JW, Walker JF, Smith SA**. **2017**. Phyx: phylogenetic tools for unix. *Bioinformatics* **33**: 1886–1888.

**Burnham KP, Anderson D**. **2002**. *Model Selection and Multimodel Inference: A Practical Information-Theoretic Approach*. New York: Springer-Verlag.

**Camacho C, Coulouris G, Avagyan V, Ma N, Papadopoulos J, Bealer K, Madden TL**. **2009**. BLAST+: architecture and applications. *BMC Bioinformatics* **10**: 421.

**Christinet L, Burdet FX, Zaiko M, Hinz U, Zrÿd J-P**. **2004**. Characterization and functional identification of a novel plant 4,5-extradiol dioxygenase involved in betalain pigment biosynthesis in *Portulaca grandiflora*. *Plant Physiology* **134**: 265–274.

**Chung H-H, Schwinn KE, Ngo HM, Lewis DH, Massey B, Calcott KE, Crowhurst R, Joyce DC, Gould KS, Davies KM, *et al.* 2015**. Characterisation of betalain biosynthesis in *Parakeelya* flowers identifies the key biosynthetic gene DOD as belonging to an expanded LigB gene family that is conserved in betalain-producing species. *Frontiers in Plant Science* **6**: 1–16.

**Copetti D, Búrquez A, Bustamante E, Charboneau JLM, Childs KL, Eguiarte LE, Lee S, Liu TL, McMahon MM, Whiteman NK, *et al.* 2017**. Extensive gene tree discordance and hemiplasy shaped the genomes of North American columnar cacti. *Proceedings of the National Academy of Sciences* **114**: 12003–12008.

**Cuénoud P, Savolainen V, Chatrou LW, Powell M, Grayer RJ, Chase MW. 2002.** Molecular phylogenetics of Caryophyllales based on nuclear 18S rDNA and plastid rbcL, atpB, and matK DNA sequences. *American Journal of Botany* **89**: 132–144.

**Clement JS, Mabry TJ, Wyler H, Dreiding AS. 1994.** Chemical Review and Evolutionary Significance of the Betalains. In: Behnke H-D, Mabry TJ, eds. Caryophyllales: Evolution and Systematics. Berlin, Heidelberg: Springer Berlin Heidelberg, 247–261.

**Dohm JC, Minoche AE, Holtgräwe D, Capella-Gutiérrez S, Zakrzewski F, Tafer H, Rupp O, Sörensen TR, Stracke R, Reinhardt R, *et al.* 2014**. The genome of the recently domesticated crop plant sugar beet (*Beta vulgaris*). *Nature* **505**: 546–9.

**Fu L, Niu B, Zhu Z, Wu S, Li W**. **2012**. CD-HIT: accelerated for clustering the next-generation sequencing data. *Bioinformatics* **28**: 3150–3152.

**Gandía-Herrero F, García-Carmona F**. **2012**. Characterization of recombinant *Beta vulgaris* 4,5-DOPA-extradiol-dioxygenase active in the biosynthesis of betalains. *Planta* **236**: 91–100.

**Guindon S, Dufayard JF, Lefort V, Anisimova M, Hordijk W, Gascuel O**. **2010**. New algorithms and methods

to estimate maximum-likelihood phylogenies: assessing the performance of PhyML 3.0. *Systematic Biology* **59**: 307–321.

**Hatlestad GJ, Sunnadeniya RM, Akhavan NA, Gonzalez A, Goldman IL, McGrath JM, Lloyd AM**. **2012**. The beet R locus encodes a new cytochrome P450 required for red betalain production. *Nature Genetics* **44**: 816–820.

**Huelsenbeck JP, Nielsen R, Bollback JP**. **2003**. Stochastic mapping of morphological characters. *Systematic Biology* **52**: 131–158.

**Huerta-Cepas J, Serra F, Bork P**. **2016**. ETE 3: reconstruction, analysis, and visualization of phylogenomic data. *Molecular Biology and Evolution* **33**: 1635–1638.

**Imamura T, Takagi H, Miyazato A, Ohki S, Mizukoshi H, Mori M**. **2018**. Isolation and characterization of the betalain biosynthesis gene involved in hypocotyl pigmentation of the allotetraploid *Chenopodium quinoa*. *Biochemical and Biophysical Research Communications* **496**: 280–286.

**Kalyaanamoorthy S, Minh BQ, Wong TKF, Von Haeseler A, Jermiin LS**. **2017**. ModelFinder: fast model selection for accurate phylogenetic estimates. *Nature Methods* **14**: 587–589.

**Katoh K, Standley DM**. **2013**. MAFFT multiple sequence alignment software version 7: improvements in performance and usability. *Molecular Biology and Evolution* **30**: 772–80.

**Lewis PO**. **2001**. A likelihood approach to estimating phylogeny from discrete morphological character data. *Systematic Biology* **50**: 913-925.

**Lopez-Nieves S, Yang Y, Timoneda A, Wang M, Feng T, Smith SA, Brockington SF, Maeda HA**. **2018**. Relaxation of tyrosine pathway regulation underlies the evolution of betalain pigmentation in Caryophyllales. *New Phytologist* **217**: 896–908.

**Luo R, Liu B, Xie Y, Li Z, Huang W, Yuan J, He G, Chen Y, Pan Q, Liu Y, *et al.*** **2012**. SOAPdenovo2: an empirically improved memory-efficient short-read de novo assembler. *GigaScience* **1**: 18.

**Magallón S, Gómez-Acevedo S, Sánchez-Reyes LL, Hernández-Hernández T**. **2015**. A metacalibrated time-tree documents the early rise of flowering plant phylogenetic diversity. *New Phytologist* **207**: 437–453.

**Nguyen LT, Schmidt HA, Von Haeseler A, Minh BQ**. **2015**. IQ-TREE: a fast and effective stochastic algorithm for estimating maximum-likelihood phylogenies. *Molecular Biology and Evolution* **32**: 268–274.

**Nielsen R**. **2002**. Mapping mutations on phylogenies. *Systematic Biology* **51**: 729–739.

**Pagel M**. **1994**. Detecting correlated evolution on phylogenies: a general method for the comparative analysis of discrete characters. *Proceedings of the Royal Society B* **255**: 37–45.

**Pagel M**. **1999**. The maximum likelihood approach to reconstructing ancestral character states of discrete characters on phylogenies. *Systematic Biology* **48**: 612–622.

**Paradis E, Schliep K**. **2019**. Ape 5.0: an environment for modern phylogenetics and evolutionary analyses in R. *Bioinformatics* **35**: 526–528.

**Price MN, Dehal PS, Arkin AP**. **2010**. FastTree 2 - approximately maximum-likelihood trees for large alignments. *PLoS ONE* **5**: e9490.

**Pucker B, Feng T, Brockington SF**. **2019**. Next generation sequencing to investigate genomic diversity in

Caryophyllales. *bioRxiv*. doi: https://doi.org/10.1101/646133

**R Core Team**. **2019**. R: a language and environment for statistical computing. Version 3.6.1. R Foundation for Statistical Computing, Vienna, Austria. URL http://www.R-project.org/

**Ranwez V, Douzery EJP, Cambon C, Chantret N, Delsuc F**. **2018**. MACSE v2: toolkit for the alignment of coding sequences accounting for frameshifts and stop codons. *Molecular Biology and Evolution* **35**: 2582–2584.

**Revell LJ**. **2012**. phytools: an R package for phylogenetic comparative biology (and other things). *Methods in Ecology and Evolution* **3**: 217–223.

**Rognes T**. **2011**. Faster Smith-Waterman database searches with inter-sequence SIMD parallelisation. *BMC Bioinformatics* **12**: 221.

**Sanderson MJ. 2002.** Estimating absolute rates of molecular evolution and divergence times: a penalized likelihood approach. *Molecular Biology and Evolution* **19**: 101–109.

**Sasaki N, Abe Y, Goda Y, Adachi T, Kasahara K, Ozeki Y**. **2009**. Detection of DOPA 4,5-dioxygenase (DOD) activity using recombinant protein prepared from *Escherichia coli* cells harboring cDNA encoding DOD from *Mirabilis jalapa*. *Plant and Cell Physiology* **50**: 1012–1016.

**Smith SA, Brown JW, Yang Y, Bruenn R, Drummond CP, Brockington SF, Walker JF, Last N, Douglas NA, Moore MJ**. **2018**. Disparity, diversity, and duplications in the Caryophyllales. *New Phytologist* **217**: 836–854.

**Smith SA, O'Meara BC**. **2012**. TreePL: divergence time estimation using penalized likelihood for large phylogenies. *Bioinformatics* **28**: 2689–2690.

**Smith SA, Walker JF**. **2019**. PyPHLAWD: a python tool for phylogenetic dataset construction. *Methods in Ecology and Evolution* **10**: 104–108.

**Stamatakis A**. **2014**. RAxML version 8: a tool for phylogenetic analysis and post-analysis of large phylogenies. *Bioinformatics* **30**: 1312–1313.

**Stanke M, Diekhans M, Baertsch R, Haussler D**. **2008**. Using native and syntenically mapped cDNA alignments to improve *de novo* gene finding. *Bioinformatics* **24**: 637–644.

**Thulin M, Larsson A, Edwards EJ, Moore AJ**. **2018**. Phylogeny and systematics of *Kewa* (Kewaceae) . *Systematic Botany* **43**: 689–700.

**Thulin M, Moore AJ, El-Seedi H, Larsson A, Christin PA, Edwards EJ**. **2016**. Phylogeny and generic delimitation in Molluginaceae, new pigment data in Caryophyllales, and the new family Corbichoniaceae. *Taxon* **65**: 775–793.

**Wagih O**. **2017**. Ggseqlogo: a versatile R package for drawing sequence logos. *Bioinformatics* **33**: 3645–3647.

**Walker JF, Yang Y, Feng T, Timoneda A, Mikenas J, Hutchinson V, Edwards C, Wang N, Ahluwalia S, Olivieri J, *et al.* 2018**. From cacti to carnivores: improved phylotranscriptomic sampling and hierarchical homology inference provides further insight to the evolution of Caryophyllales. *American Journal of Botany* **105**: 446–462.

***New Phytologist Supporting Information Figs S1, S3, S6 & S13–S15***

Article title: Evolution of L-DOPA 4,5-dioxygenase activity allows for recurrent specialisation to betalain pigmentation in Caryophyllales

Authors: Hester Sheehan*,Tao Feng*, Nathanael Walker-Hale*, Samuel Lopez-Nieves, Boas Pucker, Rui Guo, Won C. Yim, Roshani Badgami, Alfonso Timoneda, Lijun Zhao, Helene Tiley, Dario Copetti, Michael J. Sanderson, John C. Cushman, Michael J. Moore, Stephen A. Smith and Samuel F. Brockington

Article acceptance date: 22 July 2019

# Figure S1

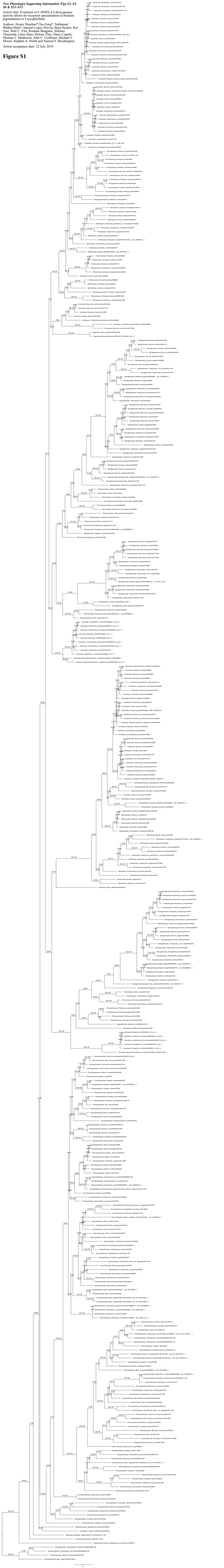

**Figure S3**

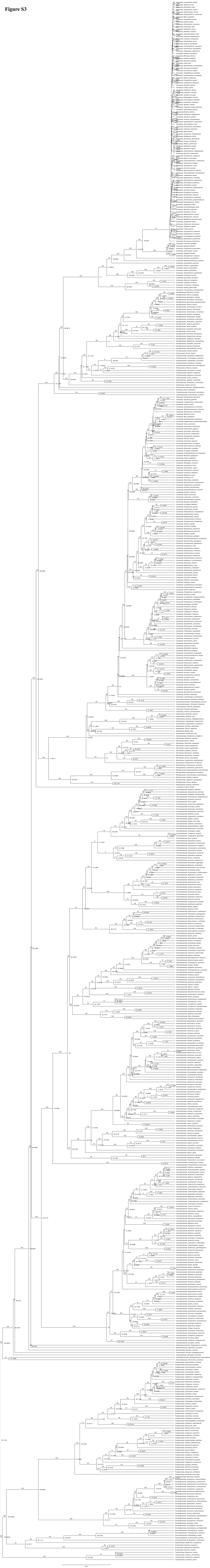

**Figure S6**

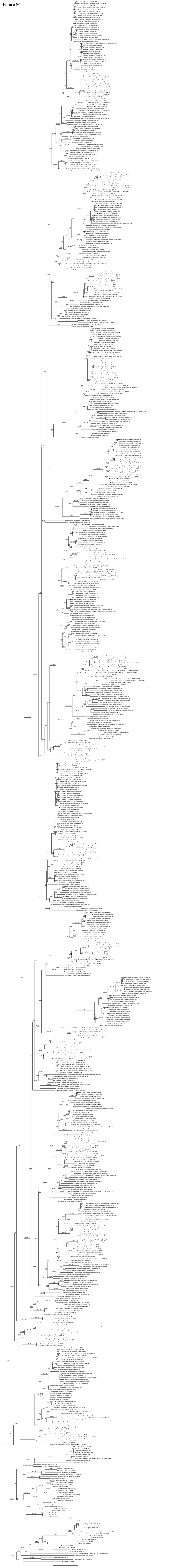

# Figure S13

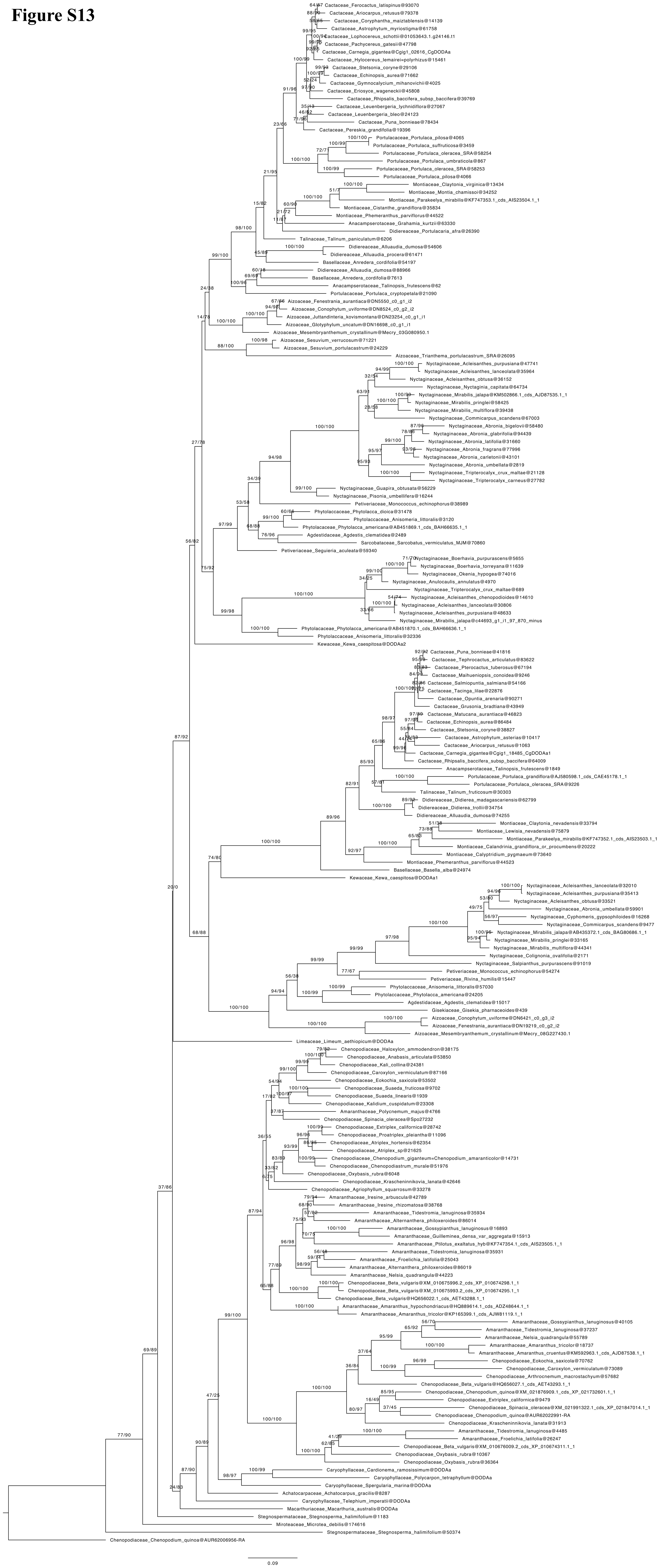

Cactaceae_Ferocactus_latispinus@93070
Cactaceae_Ariocarpus_retusus@79378
Cactaceae_Coryphantha_maiztablensis@14139
Cactaceae_Astrophytum_myriostigma@61758
Cactaceae_Lophocereus_schottii@01053643.1.g24146.t1
Cactaceae_Pachycereus_gatesii@47798
Cactaceae_Carnegia_gigantea@Cgig1_02616_CgDODAa
Cactaceae_Hylocereus_lemairei=polyrhizus@15461
Cactaceae_Stetsonia_coryne@29106
Cactaceae_Echinopsis_aurea@71662
Cactaceae_Gymnocalycium_mihanovichii@4025
Cactaceae_Eriosyce_wagenecki@45808
Cactaceae_Rhipsalis_baccifera_subsp_baccifera@39769
Cactaceae_Leuenbergeria_lychnidiflora@27067
Cactaceae_Leuenbergeria_bleo@24123
Cactaceae_Puna_bonnieae@78434
Cactaceae_Pereskia_grandifolia@19396
Portulacaceae_Portulaca_pilosa@4065
Portulacaceae_Portulaca_suffruticosa@3459
Portulacaceae_Portulaca_oleracea_SRA@58254
Portulacaceae_Portulaca_umbraticola@867
Portulacaceae_Portulaca_oleracea_SRA@58253
Portulacaceae_Portulaca_pilosa@4066
Montiaceae_Claytonia_virginica@13434
Montiaceae_Montia_chamissoi@34252
Montiaceae_Parakeelya_mirabilis@KF747353.1_cds_AIS23504.1_1
Montiaceae_Cistanthe_grandiflora@35834
Montiaceae_Phemeranthus_parviflorus@44522
Anacampserotaceae_Grahamia_kurtzii@63330
Didiereaceae_Portulacaria_afra@26390
Talinaceae_Talinum_paniculatum@6206
Didiereaceae_Alluaudia_dumosa@54606
Didiereaceae_Alluaudia_procera@61471
Basellaceae_Anredera_cordifolia@54197
Didiereaceae_Alluaudia_dumosa@88966
Basellaceae_Anredera_cordifolia@7613
Anacampserotaceae_Talinopsis_frutescens@62
Portulacaceae_Portulaca_cryptopetala@21090
Aizoaceae_Fenestrania_aurantiaca@DN5550_c0_g1_i2
Aizoaceae_Conophytum_uviforme@DN8524_c0_g2_i2
Aizoaceae_Juttadinteria_kovismontana@DN23254_c0_g1_i1
Aizoaceae_Glotyphylum_uncatum@DN16698_c0_g1_i1
Aizoaceae_Mesembryanthemum_crystallinum@Mecry_03G080950.1
Aizoaceae_Sesuvium_verrucosum@71221
Aizoaceae_Sesuvium_portulacastrum@24229
Aizoaceae_Trianthema_portulacastrum_SRA@26095
Nyctaginaceae_Acleisanthes_purpusiana@47741
Nyctaginaceae_Acleisanthes_lanceolata@35964
Nyctaginaceae_Acleisanthes_obtusa@36152
Nyctaginaceae_Nyctaginia_capitata@64734
Nyctaginaceae_Mirabilis_jalapa@KM502866.1_cds_AJD87535.1_1
Nyctaginaceae_Mirabilis_pringlei@58425
Nyctaginaceae_Mirabilis_multiflora@39438
Nyctaginaceae_Commicarpus_scandens@67003
Nyctaginaceae_Abronia_bigelovii@58480
Nyctaginaceae_Abronia_glabrifolia@94439
Nyctaginaceae_Abronia_latifolia@31660
Nyctaginaceae_Abronia_fragrans@77996
Nyctaginaceae_Abronia_carletonii@43101
Nyctaginaceae_Abronia_umbellata@2819
Nyctaginaceae_Tripterocalyx_crux_maltae@21128
Nyctaginaceae_Tripterocalyx_carneus@27782
Nyctaginaceae_Guapira_obtusata@56229
Nyctaginaceae_Pisonia_umbellifera@16244
Petiveriaceae_Monococcus_echinophorus@38989
Phytolaccaceae_Phytolacca_dioica@31478
Phytolaccaceae_Anisomeria_littoralis@3120
Phytolaccaceae_Phytolacca_americana@AB451869.1_cds_BAH66635.1_1
Agdestidaceae_Agdestis_clematidea@2489
Sarcobataceae_Sarcobatus_vermiculatus_MJM@70860
Petiveriaceae_Seguieria_aculeata@59340
Nyctaginaceae_Boerhavia_purpurascens@5655
Nyctaginaceae_Boerhavia_torreyana@11639
Nyctaginaceae_Okenia_hypogea@74016
Nyctaginaceae_Anulocaulis_annulatus@4970
Nyctaginaceae_Tripterocalyx_crux_maltae@689
Nyctaginaceae_Acleisanthes_chenopodioides@14610
Nyctaginaceae_Acleisanthes_lanceolata@30806
Nyctaginaceae_Acleisanthes_purpusiana@48633
Nyctaginaceae_Mirabilis_jalapa@c44693_g1_i1_97_870_minus
Phytolaccaceae_Phytolacca_americana@AB451870.1_cds_BAH66636.1_1
Phytolaccaceae_Anisomeria_littoralis@32336
Kewaceae_Kewa_caespitosa@DODAa2
Cactaceae_Puna_bonnieae@41816
Cactaceae_Tephrocactus_articulatus@83622
Cactaceae_Pterocactus_tuberosus@67194
Cactaceae_Maihueniopsis_conoidea@9246
Cactaceae_Salmiopuntia_salmiana@54166
Cactaceae_Tacinga_lilae@22876
Cactaceae_Opuntia_arenaria@90271
Cactaceae_Grusonia_bradtiana@43949
Cactaceae_Matucana_aurantiaca@46823
Cactaceae_Echinopsis_aurea@86484
Cactaceae_Stetsonia_coryne@38827
Cactaceae_Astrophytum_asterias@10417
Cactaceae_Ariocarpus_retusus@1063
Cactaceae_Carnegia_gigantea@Cgig1_18485_CgDODAa1
Cactaceae_Rhipsalis_baccifera_subsp_baccifera@64009
Anacampserotaceae_Talinopsis_frutescens@1849
Portulacaceae_Portulaca_grandiflora@AJ580598.1_cds_CAE45178.1_1
Portulacaceae_Portulaca_oleracea_SRA@9226
Talinaceae_Talinum_fruticosum@30303
Didiereaceae_Didierea_madagascariensis@62799
Didiereaceae_Didierea_trollii@34754
Didiereaceae_Alluaudia_dumosa@74255
Montiaceae_Claytonia_nevadensis@33794
Montiaceae_Lewisia_nevadensis@75879
Montiaceae_Parakeelya_mirabilis@KF747352.1_cds_AIS23503.1_1
Montiaceae_Calandrinia_grandiflora_or_procumbens@20222
Montiaceae_Calyptridium_pygmaeum@73640
Montiaceae_Phemeranthus_parviflorus@44523
Basellaceae_Basella_alba@24974
Kewaceae_Kewa_caespitosa@DODAa1
Nyctaginaceae_Acleisanthes_lanceolata@32010
Nyctaginaceae_Acleisanthes_purpusiana@35413
Nyctaginaceae_Acleisanthes_obtusa@33521
Nyctaginaceae_Abronia_umbellata@59901
Nyctaginaceae_Cyphomeris_gypsophiloides@16268
Nyctaginaceae_Commicarpus_scandens@9477
Nyctaginaceae_Mirabilis_jalapa@AB435372.1_cds_BAG80686.1_1
Nyctaginaceae_Mirabilis_pringlei@33165
Nyctaginaceae_Mirabilis_multiflora@44341
Nyctaginaceae_Colignonia_ovalifolia@2171
Nyctaginaceae_Salpianthus_purpurascens@91019
Petiveriaceae_Monococcus_echinophorus@54274
Petiveriaceae_Rivina_humilis@15447
Phytolaccaceae_Anisomeria_littoralis@57030
Phytolaccaceae_Phytolacca_americana@24205
Agdestidaceae_Agdestis_clematidea@15017
Gisekiaceae_Gisekia_pharnaceoides@439
Aizoaceae_Conophytum_uviforme@DN6421_c0_g3_i2
Aizoaceae_Fenestrania_aurantiaca@DN19219_c0_g2_i2
Aizoaceae_Mesembryanthemum_crystallinum@Mecry_08G227430.1
Limeaceae_Limeum_aethiopicum@DODAa
Chenopodiaceae_Haloxylon_ammodendron@38175
Chenopodiaceae_Anabasis_articulata@53850
Chenopodiaceae_Kali_collina@24381
Chenopodiaceae_Caroxylon_vermiculatum@87166
Chenopodiaceae_Eokochia_saxicola@53502
Chenopodiaceae_Suaeda_fruticosa@9702
Chenopodiaceae_Suaeda_linearis@1939
Chenopodiaceae_Kalidium_cuspidatum@23308
Amaranthaceae_Polycnemum_majus@4766
Chenopodiaceae_Spinacia_oleracea@Spo27232
Chenopodiaceae_Extriplex_californica@28742
Chenopodiaceae_Proatriplex_pleiantha@11096
Chenopodiaceae_Atriplex_hortensis@62354
Chenopodiaceae_Atriplex_sp@21625
Chenopodiaceae_Chenopodium_giganteum=Chenopodium_amaranticolor@14731
Chenopodiaceae_Chenopodiastrum_murale@51976
Chenopodiaceae_Oxybasis_rubra@6048
Chenopodiaceae_Krascheninnikovia_lanata@42646
Chenopodiaceae_Agriophyllum_squarrosum@33278
Amaranthaceae_Iresine_arbuscula@42789
Amaranthaceae_Iresine_rhizomatosa@38768
Amaranthaceae_Tidestromia_lanuginosa@35934
Amaranthaceae_Alternanthera_philoxeroides@86014
Amaranthaceae_Gossypianthus_lanuginosus@16893
Amaranthaceae_Guilleminea_densa_var_aggregata@15913
Amaranthaceae_Ptilotus_exaltatus_hyb@KF747354.1_cds_AIS23505.1_1
Amaranthaceae_Tidestromia_lanuginosa@35931
Amaranthaceae_Froelichia_latifolia@25043
Amaranthaceae_Alternanthera_philoxeroides@86019
Amaranthaceae_Nelsia_quadrangula@44223
Chenopodiaceae_Beta_vulgaris@XM_010675996.2_cds_XP_010674298.1_1
Chenopodiaceae_Beta_vulgaris@XM_010675993.2_cds_XP_010674295.1_1
Chenopodiaceae_Beta_vulgaris@HQ656022.1_cds_AET43288.1_1
Amaranthaceae_Amaranthus_hypochondriacus@HQ889614.1_cds_ADZ48644.1_1
Amaranthaceae_Amaranthus_tricolor@KP165399.1_cds_AJW81119.1_1
Amaranthaceae_Gossypianthus_lanuginosus@40105
Amaranthaceae_Tidestromia_lanuginosa@37237
Amaranthaceae_Nelsia_quadrangula@55789
Amaranthaceae_Amaranthus_tricolor@18737
Amaranthaceae_Amaranthus_cruentus@KM592963.1_cds_AJD87538.1_1
Chenopodiaceae_Eokochia_saxicola@70762
Chenopodiaceae_Caroxylon_vermiculatum@73089
Chenopodiaceae_Arthrocnemum_macrostachyum@57682
Chenopodiaceae_Beta_vulgaris@HQ656027.1_cds_AET43293.1_1
Chenopodiaceae_Chenopodium_quinoa@XM_021876909.1_cds_XP_021732601.1_1
Chenopodiaceae_Extriplex_californica@9479
Chenopodiaceae_Spinacia_oleracea@XM_021991322.1_cds_XP_021847014.1_1
Chenopodiaceae_Chenopodium_quinoa@AUR62022991-RA
Chenopodiaceae_Krascheninnikovia_lanata@31913
Amaranthaceae_Tidestromia_lanuginosa@4485
Amaranthaceae_Froelichia_latifolia@26247
Chenopodiaceae_Beta_vulgaris@XM_010676009.2_cds_XP_010674311.1_1
Chenopodiaceae_Oxybasis_rubra@10367
Chenopodiaceae_Oxybasis_rubra@36364
Caryophyllaceae_Cardionema_ramosissimum@DODAa
Caryophyllaceae_Polycarpon_tetraphyllum@DODAa
Caryophyllaceae_Spergularia_marina@DODAa
Achatocarpaceae_Achatocarpus_gracilis@8287
Caryophyllaceae_Telephium_imperati@DODAa
Macarthuriaceae_Macarthuria_australis@DODAa
Stegnospermataceae_Stegnosperma_halimifolium@1183
Miroteaceae_Microtea_debilis@174616
Stegnospermataceae_Stegnosperma_halimifolium@50374
Chenopodiaceae_Chenopodium_quinoa@AUR62006956-RA
0.09

# Figure S14

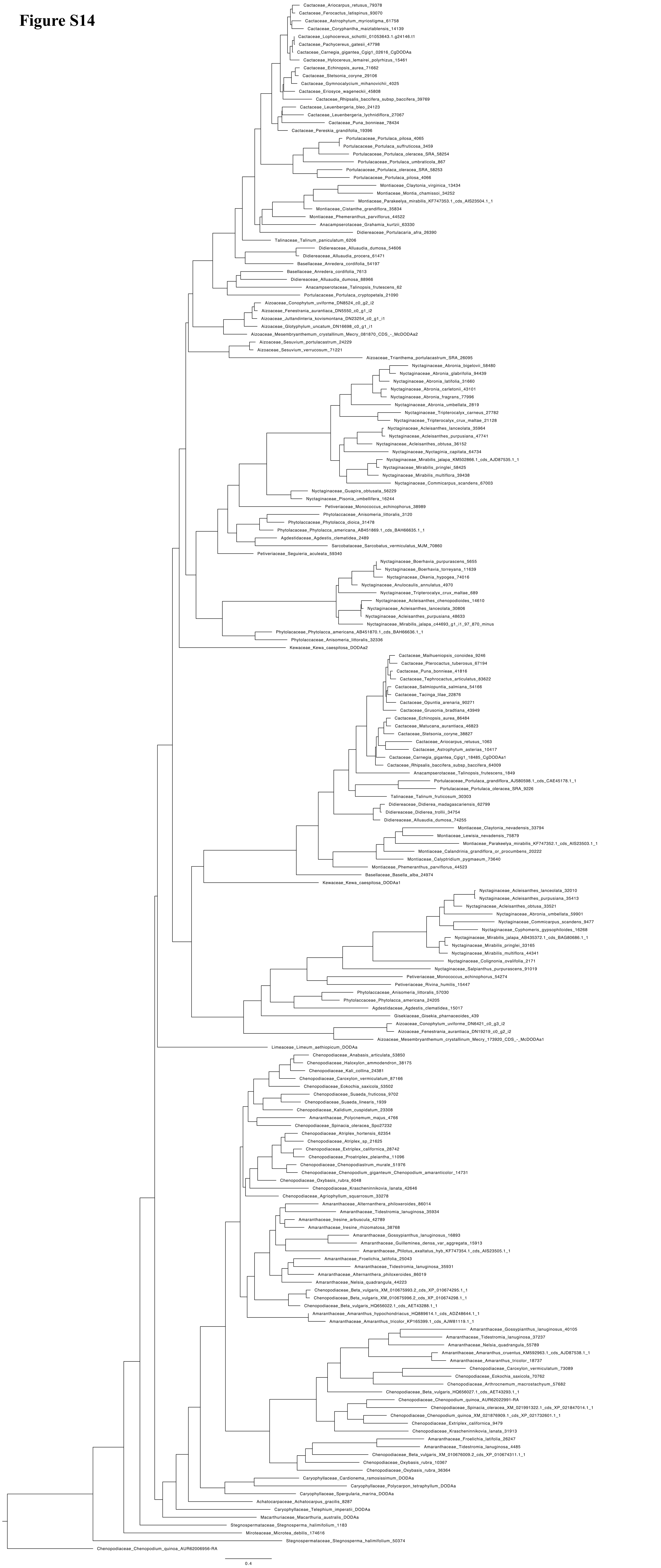

Cactaceae_Ariocarpus_retusus_79378
Cactaceae_Ferocactus_latispinus_93070
Cactaceae_Astrophytum_myriostigma_61758
Cactaceae_Coryphantha_maiztablensis_14139
Cactaceae_Lophocereus_schottii_01053643.1.g24146.t1
Cactaceae_Pachycereus_gatesii_47798
Cactaceae_Carnegia_gigantea_Cgig1_02616_CgDODAa
Cactaceae_Hylocereus_lemairei_polyrhizus_15461
Cactaceae_Echinopsis_aurea_71662
Cactaceae_Stetsonia_coryne_29106
Cactaceae_Gymnocalycium_mihanovichii_4025
Cactaceae_Eriosyce_wagenecki_45808
Cactaceae_Rhipsalis_baccifera_subsp_baccifera_39769
Cactaceae_Leuenbergeria_bleo_24123
Cactaceae_Leuenbergeria_lychnidiflora_27067
Cactaceae_Puna_bonnieae_78434
Cactaceae_Pereskia_grandifolia_19396
Portulacaceae_Portulaca_pilosa_4065
Portulacaceae_Portulaca_suffruticosa_3459
Portulacaceae_Portulaca_oleracea_SRA_58254
Portulacaceae_Portulaca_umbraticola_867
Portulacaceae_Portulaca_oleracea_SRA_58253
Portulacaceae_Portulaca_pilosa_4066
Montiaceae_Claytonia_virginica_13434
Montiaceae_Montia_chamissoi_34252
Montiaceae_Parakeelya_mirabilis_KF747353.1_cds_AIS23504.1_1
Montiaceae_Cistanthe_grandiflora_35834
Montiaceae_Phemeranthus_parviflorus_44522
Anacampserotaceae_Grahamia_kurtzii_63330
Didiereaceae_Portulacaria_afra_26390
Talinaceae_Talinum_paniculatum_6206
Didiereaceae_Alluaudia_dumosa_54606
Didiereaceae_Alluaudia_procera_61471
Basellaceae_Anredera_cordifolia_54197
Basellaceae_Anredera_cordifolia_7613
Didiereaceae_Alluaudia_dumosa_88966
Anacampserotaceae_Talinopsis_frutescens_62
Portulacaceae_Portulaca_cryptopetala_21090
Aizoaceae_Conophytum_uviforme_DN8524_c0_g2_i2
Aizoaceae_Fenestrania_aurantiaca_DN5550_c0_g1_i2
Aizoaceae_Juttadinteria_kovismontana_DN23254_c0_g1_i1
Aizoaceae_Glotyphylum_uncatum_DN16698_c0_g1_i1
Aizoaceae_Mesembryanthemum_crystallinum_Mecry_081870_CDS_-_McDODAa2
Aizoaceae_Sesuvium_portulacastrum_24229
Aizoaceae_Sesuvium_verrucosum_71221
Aizoaceae_Trianthema_portulacastrum_SRA_26095
Nyctaginaceae_Abronia_bigelovii_58480
Nyctaginaceae_Abronia_glabrifolia_94439
Nyctaginaceae_Abronia_latifolia_31660
Nyctaginaceae_Abronia_carletonii_43101
Nyctaginaceae_Abronia_fragrans_77996
Nyctaginaceae_Abronia_umbellata_2819
Nyctaginaceae_Tripterocalyx_carneus_27782
Nyctaginaceae_Tripterocalyx_crux_maltae_21128
Nyctaginaceae_Acleisanthes_lanceolata_35964
Nyctaginaceae_Acleisanthes_purpusiana_47741
Nyctaginaceae_Acleisanthes_obtusa_36152
Nyctaginaceae_Nyctaginia_capitata_64734
Nyctaginaceae_Mirabilis_jalapa_KM502866.1_cds_AJD87535.1_1
Nyctaginaceae_Mirabilis_pringlei_58425
Nyctaginaceae_Mirabilis_multiflora_39438
Nyctaginaceae_Commicarpus_scandens_67003
Nyctaginaceae_Guapira_obtusata_56229
Nyctaginaceae_Pisonia_umbellifera_16244
Petiveriaceae_Monococcus_echinophorus_38989
Phytolaccaceae_Anisomeria_littoralis_3120
Phytolaccaceae_Phytolacca_dioica_31478
Phytolaccaceae_Phytolacca_americana_AB451869.1_cds_BAH66635.1_1
Agdestidaceae_Agdestis_clematidea_2489
Sarcobataceae_Sarcobatus_vermiculatus_MJM_70860
Petiveriaceae_Seguieria_aculeata_59340
Nyctaginaceae_Boerhavia_purpurascens_5655
Nyctaginaceae_Boerhavia_torreyana_11639
Nyctaginaceae_Okenia_hypogea_74016
Nyctaginaceae_Anulocaulis_annulatus_4970
Nyctaginaceae_Tripterocalyx_crux_maltae_689
Nyctaginaceae_Acleisanthes_chenopodioides_14610
Nyctaginaceae_Acleisanthes_lanceolata_30806
Nyctaginaceae_Acleisanthes_purpusiana_48633
Nyctaginaceae_Mirabilis_jalapa_c44693_g1_i1_97_870_minus
Phytolacaceae_Phytolacca_americana_AB451870.1_cds_BAH66636.1_1
Phytolaccaceae_Anisomeria_littoralis_32336
Kewaceae_Kewa_caespitosa_DODAa2
Cactaceae_Maihueniopsis_conoidea_9246
Cactaceae_Pterocactus_tuberosus_67194
Cactaceae_Puna_bonnieae_41816
Cactaceae_Tephrocactus_articulatus_83622
Cactaceae_Salmiopuntia_salmiana_54166
Cactaceae_Tacinga_lilae_22876
Cactaceae_Opuntia_arenaria_90271
Cactaceae_Grusonia_bradtiana_43949
Cactaceae_Echinopsis_aurea_86484
Cactaceae_Matucana_aurantiaca_46823
Cactaceae_Stetsonia_coryne_38827
Cactaceae_Ariocarpus_retusus_1063
Cactaceae_Astrophytum_asterias_10417
Cactaceae_Carnegia_gigantea_Cgig1_18485_CgDODAa1
Cactaceae_Rhipsalis_baccifera_subsp_baccifera_64009
Anacampserotaceae_Talinopsis_frutescens_1849
Portulacaceae_Portulaca_grandiflora_AJ580598.1_cds_CAE45178.1_1
Portulacaceae_Portulaca_oleracea_SRA_9226
Talinaceae_Talinum_fruticosum_30303
Didiereaceae_Didierea_madagascariensis_62799
Didiereaceae_Didierea_trollii_34754
Didiereaceae_Alluaudia_dumosa_74255
Montiaceae_Claytonia_nevadensis_33794
Montiaceae_Lewisia_nevadensis_75879
Montiaceae_Parakeelya_mirabilis_KF747352.1_cds_AIS23503.1_1
Montiaceae_Calandrinia_grandiflora_or_procumbens_20222
Montiaceae_Calyptridium_pygmaeum_73640
Montiaceae_Phemeranthus_parviflorus_44523
Basellaceae_Basella_alba_24974
Kewaceae_Kewa_caespitosa_DODAa1
Nyctaginaceae_Acleisanthes_lanceolata_32010
Nyctaginaceae_Acleisanthes_purpusiana_35413
Nyctaginaceae_Acleisanthes_obtusa_33521
Nyctaginaceae_Abronia_umbellata_59901
Nyctaginaceae_Commicarpus_scandens_9477
Nyctaginaceae_Cyphomeris_gypsophiloides_16268
Nyctaginaceae_Mirabilis_jalapa_AB435372.1_cds_BAG80686.1_1
Nyctaginaceae_Mirabilis_pringlei_33165
Nyctaginaceae_Mirabilis_multiflora_44341
Nyctaginaceae_Colignonia_ovalifolia_2171
Nyctaginaceae_Salpianthus_purpurascens_91019
Petiveriaceae_Monococcus_echinophorus_54274
Petiveriaceae_Rivina_humilis_15447
Phytolaccaceae_Anisomeria_littoralis_57030
Phytolaccaceae_Phytolacca_americana_24205
Agdestidaceae_Agdestis_clematidea_15017
Gisekiaceae_Gisekia_pharnaceoides_439
Aizoaceae_Conophytum_uviforme_DN6421_c0_g3_i2
Aizoaceae_Fenestraria_aurantiaca_DN19219_c0_g2_i2
Aizoaceae_Mesembryanthemum_crystallinum_Mecry_173920_CDS_-_McDODAa1
Limeaceae_Limeum_aethiopicum_DODAa
Chenopodiaceae_Anabasis_articulata_53850
Chenopodiaceae_Haloxylon_ammodendron_38175
Chenopodiaceae_Kali_collina_24381
Chenopodiaceae_Caroxylon_vermiculatum_87166
Chenopodiaceae_Eokochia_saxicola_53502
Chenopodiaceae_Suaeda_fruticosa_9702
Chenopodiaceae_Suaeda_linearis_1939
Chenopodiaceae_Kalidium_cuspidatum_23308
Amaranthaceae_Polycnemum_majus_4766
Chenopodiaceae_Spinacia_oleracea_Spo27232
Chenopodiaceae_Atriplex_hortensis_62354
Chenopodiaceae_Atriplex_sp_21625
Chenopodiaceae_Extriplex_californica_28742
Chenopodiaceae_Proatriplex_pleiantha_11096
Chenopodiaceae_Chenopodiastrum_murale_51976
Chenopodiaceae_Chenopodium_giganteum_Chenopodium_amaranticolor_14731
Chenopodiaceae_Oxybasis_rubra_6048
Chenopodiaceae_Krascheninnikovia_lanata_42646
Chenopodiaceae_Agriophyllum_squarrosum_33278
Amaranthaceae_Alternanthera_philoxeroides_86014
Amaranthaceae_Tidestromia_lanuginosa_35934
Amaranthaceae_Iresine_arbuscula_42789
Amaranthaceae_Iresine_rhizomatosa_38768
Amaranthaceae_Gossypianthus_lanuginosus_16893
Amaranthaceae_Guilleminea_densa_var_aggregata_15913
Amaranthaceae_Ptilotus_exaltatus_hyb_KF747354.1_cds_AIS23505.1_1
Amaranthaceae_Froelichia_latifolia_25043
Amaranthaceae_Tidestromia_lanuginosa_35931
Amaranthaceae_Alternanthera_philoxeroides_86019
Amaranthaceae_Nelsia_quadrangula_44223
Chenopodiaceae_Beta_vulgaris_XM_010675993.2_cds_XP_010674295.1_1
Chenopodiaceae_Beta_vulgaris_XM_010675996.2_cds_XP_010674298.1_1
Chenopodiaceae_Beta_vulgaris_HQ656022.1_cds_AET43288.1_1
Amaranthaceae_Amaranthus_hypochondriacus_HQ889614.1_cds_ADZ48644.1_1
Amaranthaceae_Amaranthus_tricolor_KP165399.1_cds_AJW81119.1_1
Amaranthaceae_Gossypianthus_lanuginosus_40105
Amaranthaceae_Tidestromia_lanuginosa_37237
Amaranthaceae_Nelsia_quadrangula_55789
Amaranthaceae_Amaranthus_cruentus_KM592963.1_cds_AJD87538.1_1
Amaranthaceae_Amaranthus_tricolor_18737
Chenopodiaceae_Caroxylon_vermiculatum_73089
Chenopodiaceae_Eokochia_saxicola_70762
Chenopodiaceae_Arthrocnemum_macrostachyum_57682
Chenopodiaceae_Beta_vulgaris_HQ656027.1_cds_AET43293.1_1
Chenopodiaceae_Chenopodium_quinoa_AUR62022991-RA
Chenopodiaceae_Spinacia_oleracea_XM_021991322.1_cds_XP_021847014.1_1
Chenopodiaceae_Chenopodium_quinoa_XM_021876909.1_cds_XP_021732601.1_1
Chenopodiaceae_Extriplex_californica_9479
Chenopodiaceae_Krascheninnikovia_lanata_31913
Amaranthaceae_Froelichia_latifolia_26247
Amaranthaceae_Tidestromia_lanuginosa_4485
Chenopodiaceae_Beta_vulgaris_XM_010676009.2_cds_XP_010674311.1_1
Chenopodiaceae_Oxybasis_rubra_10367
Chenopodiaceae_Oxybasis_rubra_36364
Caryophyllaceae_Cardionema_ramosissimum_DODAa
Caryophyllaceae_Polycarpon_tetraphyllum_DODAa
Caryophyllaceae_Spergularia_marina_DODAa
Achatocarpaceae_Achatocarpus_gracilis_8287
Caryophyllaceae_Telephium_imperatii_DODAa
Macarthuriaceae_Macarthuria_australis_DODAa
Stegnospermataceae_Stegnosperma_halimifolium_1183
Microteaceae_Microtea_debilis_174616
Stegnospermataceae_Stegnosperma_halimifolium_50374
Chenopodiaceae_Chenopodium_quinoa_AUR62006956-RA
0.4

# Figure S15

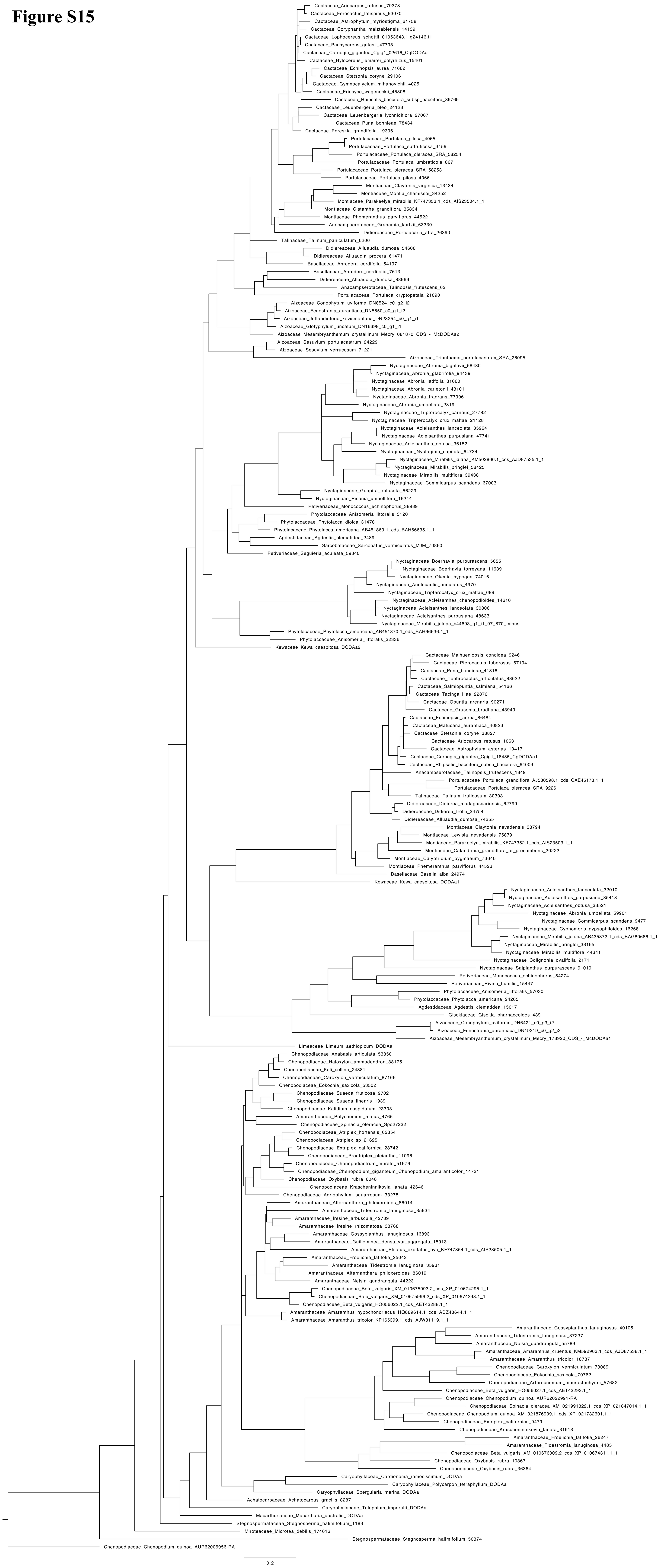

Cactaceae_Ariocarpus_retusus_79378
Cactaceae_Ferocactus_latispinus_93070
Cactaceae_Astrophytum_myriostigma_61758
Cactaceae_Coryphantha_maiztablensis_14139
Cactaceae_Lophocereus_schottii_01053643.1.g24146.t1
Cactaceae_Pachycereus_gatesii_47798
Cactaceae_Carnegia_gigantea_Cgig1_02616_CgDODAa
Cactaceae_Hylocereus_lemairei_polyrhizus_15461
Cactaceae_Echinopsis_aurea_71662
Cactaceae_Stetsonia_coryne_29106
Cactaceae_Gymnocalycium_mihanovichii_4025
Cactaceae_Eriosyce_wageneckii_45808
Cactaceae_Rhipsalis_baccifera_subsp_baccifera_39769
Cactaceae_Leuenbergeria_bleo_24123
Cactaceae_Leuenbergeria_lychnidiflora_27067
Cactaceae_Puna_bonnieae_78434
Cactaceae_Pereskia_grandifolia_19396
Portulacaceae_Portulaca_pilosa_4065
Portulacaceae_Portulaca_suffruticosa_3459
Portulacaceae_Portulaca_oleracea_SRA_58254
Portulacaceae_Portulaca_umbraticola_867
Portulacaceae_Portulaca_oleracea_SRA_58253
Portulacaceae_Portulaca_pilosa_4066
Montiaceae_Claytonia_virginica_13434
Montiaceae_Montia_chamissoi_34252
Montiaceae_Parakeelya_mirabilis_KF747353.1_cds_AIS23504.1_1
Montiaceae_Cistanthe_grandiflora_35834
Montiaceae_Phemeranthus_parviflorus_44522
Anacampserotaceae_Grahamia_kurtzii_63330
Didiereaceae_Portulacaria_afra_26390
Talinaceae_Talinum_paniculatum_6206
Didiereaceae_Alluaudia_dumosa_54606
Didiereaceae_Alluaudia_procera_61471
Basellaceae_Anredera_cordifolia_54197
Basellaceae_Anredera_cordifolia_7613
Didiereaceae_Alluaudia_dumosa_88966
Anacampserotaceae_Talinopsis_frutescens_62
Portulacaceae_Portulaca_cryptopetala_21090
Aizoaceae_Conophytum_uviforme_DN8524_c0_g2_i2
Aizoaceae_Fenestrania_aurantiaca_DN5550_c0_g1_i2
Aizoaceae_Juttadinteria_kovismontana_DN23254_c0_g1_i1
Aizoaceae_Glotyphylum_uncatum_DN16698_c0_g1_i1
Aizoaceae_Mesembryanthemum_crystallinum_Mecry_081870_CDS_-_McDODAa2
Aizoaceae_Sesuvium_portulacastrum_24229
Aizoaceae_Sesuvium_verrucosum_71221
Aizoaceae_Trianthema_portulacastrum_SRA_26095
Nyctaginaceae_Abronia_bigelovii_58480
Nyctaginaceae_Abronia_glabrifolia_94439
Nyctaginaceae_Abronia_latifolia_31660
Nyctaginaceae_Abronia_carletonii_43101
Nyctaginaceae_Abronia_fragrans_77996
Nyctaginaceae_Abronia_umbellata_2819
Nyctaginaceae_Tripterocalyx_carneus_27782
Nyctaginaceae_Tripterocalyx_crux_maltae_21128
Nyctaginaceae_Acleisanthes_lanceolata_35964
Nyctaginaceae_Acleisanthes_purpusiana_47741
Nyctaginaceae_Acleisanthes_obtusa_36152
Nyctaginaceae_Nyctaginia_capitata_64734
Nyctaginaceae_Mirabilis_jalapa_KM502866.1_cds_AJD87535.1_1
Nyctaginaceae_Mirabilis_pringlei_58425
Nyctaginaceae_Mirabilis_multiflora_39438
Nyctaginaceae_Commicarpus_scandens_67003
Nyctaginaceae_Guapira_obtusata_56229
Nyctaginaceae_Pisonia_umbellifera_16244
Petiveriaceae_Monococcus_echinophorus_38989
Phytolaccaceae_Anisomeria_littoralis_3120
Phytolaccaceae_Phytolacca_dioica_31478
Phytolaccaceae_Phytolacca_americana_AB451869.1_cds_BAH66635.1_1
Agdestidaceae_Agdestis_clematidea_2489
Sarcobataceae_Sarcobatus_vermiculatus_MJM_70860
Petiveriaceae_Seguieria_aculeata_59340
Nyctaginaceae_Boerhavia_purpurascens_5655
Nyctaginaceae_Boerhavia_torreyana_11639
Nyctaginaceae_Okenia_hypogea_74016
Nyctaginaceae_Anulocaulis_annulatus_4970
Nyctaginaceae_Tripterocalyx_crux_maltae_689
Nyctaginaceae_Acleisanthes_chenopodioides_14610
Nyctaginaceae_Acleisanthes_lanceolata_30806
Nyctaginaceae_Acleisanthes_purpusiana_48633
Nyctaginaceae_Mirabilis_jalapa_c44693_g1_i1_97_870_minus
Phytolacaceae_Phytolacca_americana_AB451870.1_cds_BAH66636.1_1
Phytolaccaceae_Anisomeria_littoralis_32336
Kewaceae_Kewa_caespitosa_DODAa2
Cactaceae_Maihueniopsis_conoidea_9246
Cactaceae_Pterocactus_tuberosus_67194
Cactaceae_Puna_bonnieae_41816
Cactaceae_Tephrocactus_articulatus_83622
Cactaceae_Salmiopuntia_salmiana_54166
Cactaceae_Tacinga_lilae_22876
Cactaceae_Opuntia_arenaria_90271
Cactaceae_Grusonia_bradtiana_43949
Cactaceae_Echinopsis_aurea_86484
Cactaceae_Matucana_aurantiaca_46823
Cactaceae_Stetsonia_coryne_38827
Cactaceae_Ariocarpus_retusus_1063
Cactaceae_Astrophytum_asterias_10417
Cactaceae_Carnegia_gigantea_Cgig1_18485_CgDODAa1
Cactaceae_Rhipsalis_baccifera_subsp_baccifera_64009
Anacampserotaceae_Talinopsis_frutescens_1849
Portulacaceae_Portulaca_grandiflora_AJ580598.1_cds_CAE45178.1_1
Portulacaceae_Portulaca_oleracea_SRA_9226
Talinaceae_Talinum_fruticosum_30303
Didiereaceae_Didierea_madagascariensis_62799
Didiereaceae_Didierea_trollii_34754
Didiereaceae_Alluaudia_dumosa_74255
Montiaceae_Claytonia_nevadensis_33794
Montiaceae_Lewisia_nevadensis_75879
Montiaceae_Parakeelya_mirabilis_KF747352.1_cds_AIS23503.1_1
Montiaceae_Calandrinia_grandiflora_or_procumbens_20222
Montiaceae_Calyptridium_pygmaeum_73640
Montiaceae_Phemeranthus_parviflorus_44523
Basellaceae_Basella_alba_24974
Kewaceae_Kewa_caespitosa_DODAa1
Nyctaginaceae_Acleisanthes_lanceolata_32010
Nyctaginaceae_Acleisanthes_purpusiana_35413
Nyctaginaceae_Acleisanthes_obtusa_33521
Nyctaginaceae_Abronia_umbellata_59901
Nyctaginaceae_Commicarpus_scandens_9477
Nyctaginaceae_Cyphomeris_gypsophiloides_16268
Nyctaginaceae_Mirabilis_jalapa_AB435372.1_cds_BAG80686.1_1
Nyctaginaceae_Mirabilis_pringlei_33165
Nyctaginaceae_Mirabilis_multiflora_44341
Nyctaginaceae_Colignonia_ovalifolia_2171
Nyctaginaceae_Salpianthus_purpurascens_91019
Petiveriaceae_Monococcus_echinophorus_54274
Petiveriaceae_Rivina_humilis_15447
Phytolaccaceae_Anisomeria_littoralis_57030
Phytolaccaceae_Phytolacca_americana_24205
Agdestidaceae_Agdestis_clematidea_15017
Gisekiaceae_Gisekia_pharnaceoides_439
Aizoaceae_Conophytum_uviforme_DN6421_c0_g3_i2
Aizoaceae_Fenestrania_aurantiaca_DN19219_c0_g2_i2
Aizoaceae_Mesembryanthemum_crystallinum_Mecry_173920_CDS_-_McDODAa1
Limeaceae_Limeum_aethiopicum_DODAa
Chenopodiaceae_Anabasis_articulata_53850
Chenopodiaceae_Haloxylon_ammodendron_38175
Chenopodiaceae_Kali_collina_24381
Chenopodiaceae_Caroxylon_vermiculatum_87166
Chenopodiaceae_Eokochia_saxicola_53502
Chenopodiaceae_Suaeda_fruticosa_9702
Chenopodiaceae_Suaeda_linearis_1939
Chenopodiaceae_Kalidium_cuspidatum_23308
Amaranthaceae_Polycnemum_majus_4766
Chenopodiaceae_Spinacia_oleracea_Spo27232
Chenopodiaceae_Atriplex_hortensis_62354
Chenopodiaceae_Atriplex_sp_21625
Chenopodiaceae_Extriplex_californica_28742
Chenopodiaceae_Proatriplex_pleiantha_11096
Chenopodiaceae_Chenopodiastrum_murale_51976
Chenopodiaceae_Chenopodium_giganteum_Chenopodium_amaranticolor_14731
Chenopodiaceae_Oxybasis_rubra_6048
Chenopodiaceae_Krascheninnikovia_lanata_42646
Chenopodiaceae_Agriophyllum_squarrosum_33278
Amaranthaceae_Alternanthera_philoxeroides_86014
Amaranthaceae_Tidestromia_lanuginosa_35934
Amaranthaceae_Iresine_arbuscula_42789
Amaranthaceae_Iresine_rhizomatosa_38768
Amaranthaceae_Gossypianthus_lanuginosus_16893
Amaranthaceae_Guilleminea_densa_var_aggregata_15913
Amaranthaceae_Ptilotus_exaltatus_hyb_KF747354.1_cds_AIS23505.1_1
Amaranthaceae_Froelichia_latifolia_25043
Amaranthaceae_Tidestromia_lanuginosa_35931
Amaranthaceae_Alternanthera_philoxeroides_86019
Amaranthaceae_Nelsia_quadrangula_44223
Chenopodiaceae_Beta_vulgaris_XM_010675993.2_cds_XP_010674295.1_1
Chenopodiaceae_Beta_vulgaris_XM_010675996.2_cds_XP_010674298.1_1
Chenopodiaceae_Beta_vulgaris_HQ656022.1_cds_AET43288.1_1
Amaranthaceae_Amaranthus_hypochondriacus_HQ889614.1_cds_ADZ48644.1_1
Amaranthaceae_Amaranthus_tricolor_KP165399.1_cds_AJW81119.1_1
Amaranthaceae_Gossypianthus_lanuginosus_40105
Amaranthaceae_Tidestromia_lanuginosa_37237
Amaranthaceae_Nelsia_quadrangula_55789
Amaranthaceae_Amaranthus_cruentus_KM592963.1_cds_AJD87538.1_1
Amaranthaceae_Amaranthus_tricolor_18737
Chenopodiaceae_Caroxylon_vermiculatum_73089
Chenopodiaceae_Eokochia_saxicola_70762
Chenopodiaceae_Arthrocnemum_macrostachyum_57682
Chenopodiaceae_Beta_vulgaris_HQ656027.1_cds_AET43293.1_1
Chenopodiaceae_Chenopodium_quinoa_AUR62022991-RA
Chenopodiaceae_Spinacia_oleracea_XM_021991322.1_cds_XP_021847014.1_1
Chenopodiaceae_Chenopodium_quinoa_XM_021876909.1_cds_XP_021732601.1_1
Chenopodiaceae_Extriplex_californica_9479
Chenopodiaceae_Krascheninnikovia_lanata_31913
Amaranthaceae_Froelichia_latifolia_26247
Amaranthaceae_Tidestromia_lanuginosa_4485
Chenopodiaceae_Beta_vulgaris_XM_010676009.2_cds_XP_010674311.1_1
Chenopodiaceae_Oxybasis_rubra_10367
Chenopodiaceae_Oxybasis_rubra_36364
Caryophyllaceae_Cardionema_ramosissimum_DODAa
Caryophyllaceae_Polycarpon_tetraphyllum_DODAa
Caryophyllaceae_Spergularia_marina_DODAa
Achatocarpaceae_Achatocarpus_gracilis_8287
Caryophyllaceae_Telephium_imperatii_DODAa
Macarthuriaceae_Macarthuria_australis_DODAa
Stegnospermataceae_Stegnosperma_halimifolium_1183
Miroteaceae_Microtea_debilis_174616
Stegnospermataceae_Stegnosperma_halimifolium_50374
Chenopodiaceae_Chenopodium_quinoa_AUR62006956-RA
0.2
